# Solvent-free synthesis of enantioenriched β-silyl nitroalkanes under organocatalytic conditions

**DOI:** 10.3762/bjoc.17.177

**Published:** 2021-10-27

**Authors:** Akhil K Dubey, Raghunath Chowdhury

**Affiliations:** 1 Bio-Organic Division, Bhabha Atomic Research Centre, Trombay, Mumbai 400085, India; 2 Homi Bhabha National Institute, Anushaktinagar, Mumbai-400094, India

**Keywords:** β-silyl α,β-unsaturated carbonyl compounds, β-silyl nitroalkanes, chiral organosilanes, organocatalysis, solvent-free synthesis

## Abstract

An enantioselective 1,4-conjugate addition of nitromethane to β-silyl α,β-unsaturated carbonyl compounds catalyzed by bifunctional squaramide catalysts has been developed. This methodology offers both enantiomers of β-silyl nitroalkanes in good to excellent yields (up to 92%) and enantioselectivities (up to 97.5% ee) under solvent-free conditions at room temperature. Control experiments reveal that the presence of a β-silyl group in the enones is crucial for high reactivity under the optimized reaction conditions.

## Introduction

**Scheme 1:**
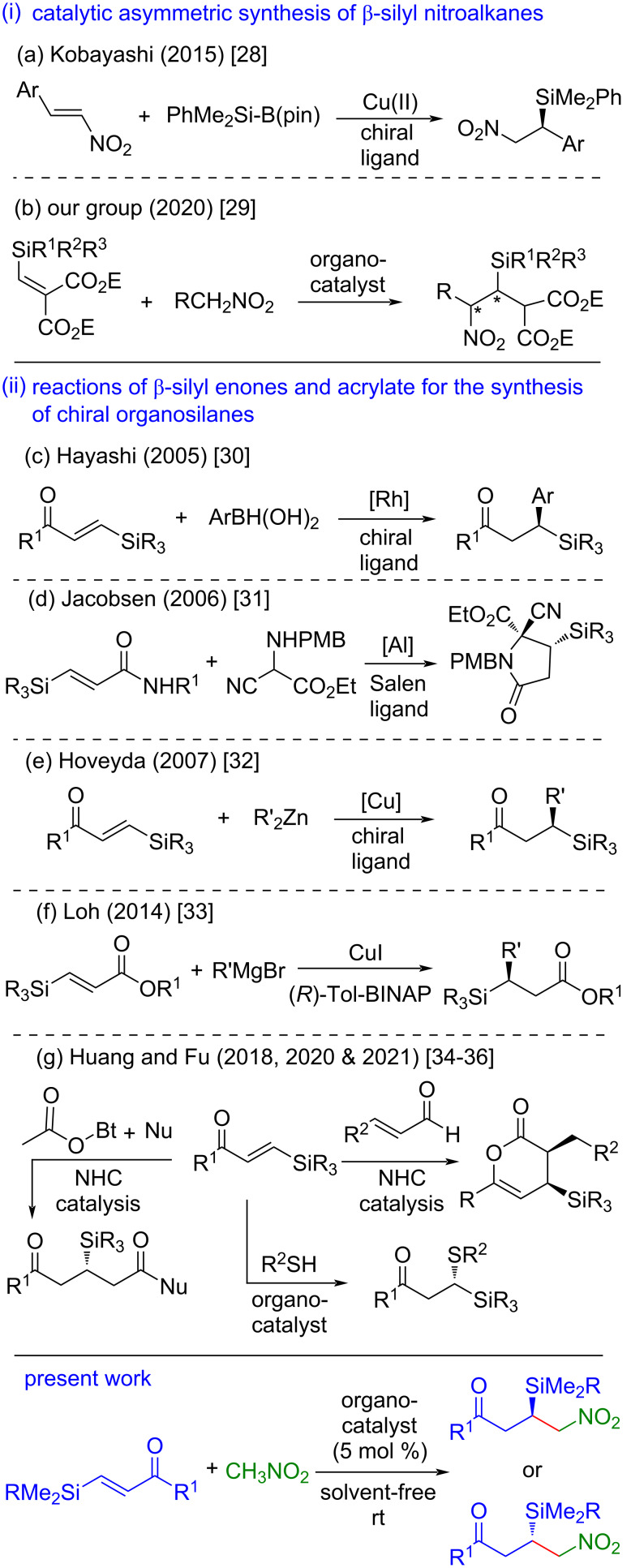
Selected methods for the synthesis of enantioenriched β-silyl nitroalkanes, synthesis of chiral organosilanes from β-silyl α,β-unsaturated carbonyl compounds, and the present work.

Enantioenriched organosilanes are attractive molecules in organic synthesis owing to their potential applications in stereoselective synthesis [1–2]. The unique sterical and electronical features of the C–Si bond can induce stereodifferentiation at the adjacent prostereogenic center in organic transformations [2]. In addition, the C–Si bond can be oxidized to a hydroxy group by Tamao–Fleming oxidation [3–4] or to an alkene unit via protodesilylation [5–6]. Many complex natural products, bioactive molecules, and drug molecules have been synthesized on exploitation of the above-mentioned properties of organosilanes [2,7–14]. A number of efficient catalytic enantioselective methods has been developed for the synthesis of chiral organosilanes [15–24]. Out of the chiral organosilanes, nitrosilanes are important synthetic targets as they are precursors of valuable β-aminosilanes [25–27]. Although there is huge success in the synthesis of enantioenriched organosilanes, catalytic routes to synthesize chiral β-nitrosilanes and in general nitrosilanes have not been well explored. Kobayashi and co-workers realized the synthesis of enantioenriched β-nitrosilanes through a Cu(II)–chiral bipyridine complex catalyzed enantioselective silyl transfer reaction to nitroalkenes using Suginome’s silylboron reagent (Scheme 1a) [28]. Recently, we have reported the synthesis of chiral β-nitrosilanes via an organocatalytic conjugate addition of nitromethane to β-silylmethylene malonates (Scheme 1b) [29]. As the catalytic enantioselective route is limited to accessible β-nitrosilanes, there is an urgent need to develop efficient catalytic protocols to deliver enantioenriched β-nitrosilanes from easily available starting materials.

Metal-catalyzed reaction of various nucleophiles to β-silyl α,β-unsaturated carbonyl compounds were documented as one of the straightforward and atom-economic approaches for the facile synthesis of chiral organosilanes (Scheme 1c–f) [30–33]. Recently, the aforementioned reaction under organocatalytic conditions has gained attention [34–36]. In this context, Huang, Fu and co-workers reported carbene-catalyzed enantioselective formal [4 + 2] annulation reactions of β-silyl enones with enals and with active acetic esters (Scheme 1g) for the preparation of chiral organosilanes [34–36]. Very recently, during the final stage of our work, the same group disclosed an organocatalyzed conjugate addition of thiols to β-silyl enones for the synthesis of chiral α-mercaptosilanes (Scheme 1g) [36].

As a part of our ongoing program for the development of asymmetric catalytic approaches for the synthesis of enantioenriched organosilanes [29,37–38], we present herein an organocatalyzed conjugate addition reaction of nitromethane to β-silyl enones to afford chiral β-silyl nitroalkanes (Scheme 1). Notably, the developed method was not only carried out under solvent-free conditions at room temperature but was found to be tolerant to moisture and air. Therefore, this method offers an attractive and robust option for the preparation of chiral β-silyl nitroalkanes. In sharp contrast to the aforesaid reaction, organocatalytic conjugate addition reactions of nitroalkanes to enones have been well studied [39–43]. To the best of our knowledge, organocatalyzed or metal-catalyzed enantioselective conjugate additions of nitroalkanes to β-silyl enones are not yet known.

## Results and Discussion

The optimization study began with the conjugate addition reaction between β-TMS enone **1a** and nitromethane (**2**) as the model reaction. An uncatalyzed background reaction was not observed while performing the model reaction in toluene as a solvent at 30 °C for 24 h. To our delight, when the same reaction was carried out in presence of 5 mol % catalyst **I** in toluene at 30 °C for 48 h, the desired product **3a** was obtained in 84% yield with 60% ee (Table 1, entry 1). Catalyst **II** was found to be unproductive as only 25% conversion of β-TMS enone **1a** was observed (Table 1, entry 2). Gratifyingly, catalyst **III** furnished product *ent*-**3a** in 85% yield (Table 1, entry 3) with excellent enantioselectivity (94% ee). Whereas catalyst **IV** gave *ent*-**3a** in 85% yield with slightly lower enantioselectivity (91% ee) as compared to catalyst **III** (Table 1, entry 4). Catalyst **V** also led to product **3a** in 66% yield and 78% ee (Table 1, entry 5). Catalyst **VI**, a pseudoenantiomer of catalyst **V** delivered *ent*-**3a** in 78% yield with 80% ee (Table 1, entry 6). The catalytic performance of the squaramide catalysts was also explored for the model reaction. Catalyst **VII** afforded the conjugate addition product **3a** in 78% yield with excellent enantiopurity of 97% ee (Table 1, entry 7). A solvent survey (see Supporting Information File 1 for details) revealed that toluene is the most suitable solvent. Next, we targeted to make the reaction more time economical under mild conditions. For this purpose, the reaction was performed at different concentrations of the reaction mixture (Table 1, entries 8–11). It was observed that time required for completion of the reaction decreased with an increase of concentration of the reaction mixture while the enantiopurity of the product **3a** remained unchanged (Table 1, entries 7–9). Next, the model reaction was performed using 10 equivalents of nitromethane (**2**) in the presence of 5 mol % catalyst **VII** under solvent-free conditions, and was complete within 24 h without affecting the enantioselectivity of product **3a** (Table 1, entry 10). Reducing the loading of nitromethane (**2**) to 5 equivalents, a slight drop in yield (82%) of product **3a** was observed whereas the enantioselectivity (97% ee) remained the same (Table 1, entry11). Upon further reduction in the loading of nitromethane (**2**) to 2.5 equivalents, the yield (82%), enantioselectivity (97% ee), and reaction time were not affected (Table 1, entry 12). Moreover, the reaction became sluggish when conducting the reaction with 2.5 mol % of the catalyst **VII** while keeping other parameters fixed (Table 1, entry 13). Performing the reaction with catalyst **VIII**, the pseudoenantiomeric catalyst of **VII**, furnished *ent*-**3a** in 80% yield and 94% ee (Table 1, entry 14). From the aforementioned studies, compromising slight lower yield of **3a**, we set up the optimization conditions as: For **3a**, **1a** (0.2 mmol), **2** (0.5 mmol), 5 mol % of catalyst **VII** at 30–32 °C (Table 1, entry 12) and for *ent*-**3a**, **1a** (0.2 mmol), **2** (0.5 mmol), 5 mol % of catalyst **VIII** at 30–32 °C (Table 1, entry 14).

**Table 1:** Catalysts screening and optimization of reaction conditions.^a^

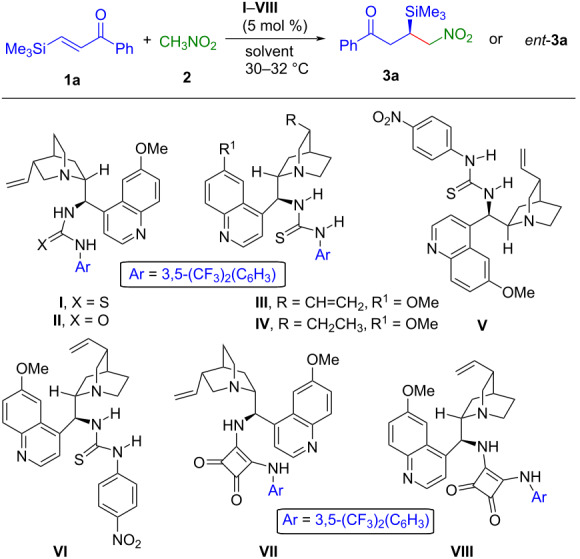						

Entry	Cat.	Solvent (mL)	**2a** (equiv)	Time (h)	Yield (%)^b^	ee (%)^c^

1	**I**	toluene (0.4)	10	48	84 (98)	60
2	**II**	toluene (0.4)	10	48	ND (25)	ND
3	**III**	toluene (0.4)	10	48	85 (>99)	−94^d^
4	**IV**	toluene (0.4)	10	48	85 (>99)	−91^d^
5	**V**	toluene (0.4)	10	48	66 (90)	78
6	**VI**	toluene (0.4)	10	48	78 (97)	−80^d^
7	**VII**	toluene (0.4)	10	48	78 (>99)	97
8	**VII**	toluene (0.2)	10	42	78 (>99)	97
9	**VII**	toluene (0.1)	10	24	80 (>99)	97
10	**VII**	–	10	24	83 (>99)	97
11	**VII**	–	5	24	82 (>99)	97
12	**VII**	–	2.5	24	82 (>99)	97
13^e^	**VII**	–	10	24	56 (85)	97
14	**VIII**	–	2.5	24	80 (>97)	−94^d^

^a^Reaction conditions: **1a** (0.2 mmol), **2** (0.5–2.0 mmol), catalyst (0.01 mmol, 5 mol %) in toluene or neat at 30–32 °C. ^b^Isolated yield after column chromatography, % of conversion of the starting material **1a** is given in parentheses, determined by ^1^H NMR analysis of the crude reaction mixture. ^c^Determined by HPLC using a chiralpak OD-H column. ^d^Opposite enantiomer. ^e^2.5 mol % of the catalyst **VII** was used.

**Scheme 2:**
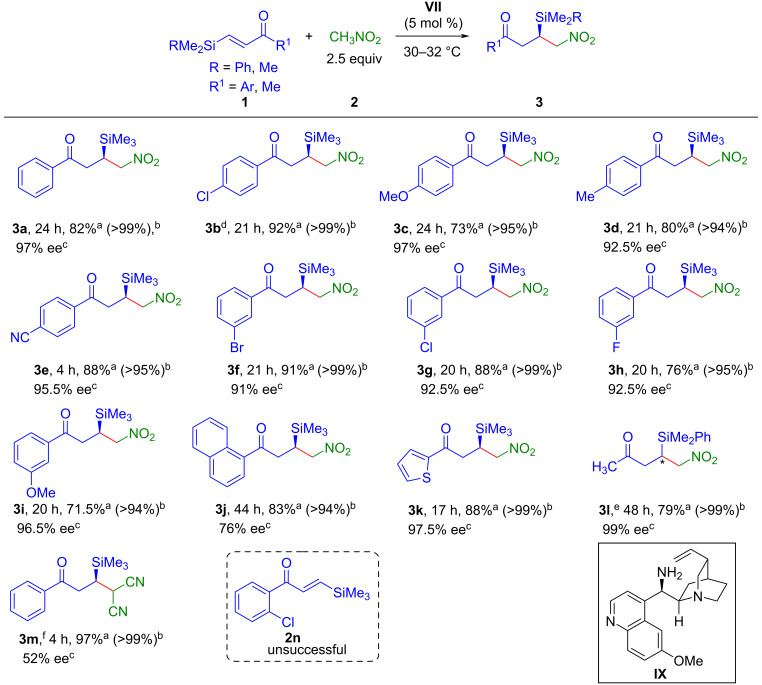
Scope of substrates. Reaction conditions: **1** (0.2 mmol), **2** (0.5 mmol), catalyst **VII** (0.01 mmol, 5 mol %) at 30 °C. ^a^Isolated yield of **3** after column chromatography. ^b^Conversion in % of the starting material **1** is given in parentheses, determined by ^1^H NMR analysis of the crude reaction mixture. ^c^Determined by HPLC using a chiral stationary phase. ^d^Enantiomers could not be separated by AD-H, OD-H, OJ-H, and AS-H columns. ^e^Reaction conditions for **3l**: **1l** (0.2 mmol), **2** (2 mmol), catalyst **IX** (0.04 mmol, 20 mol %), benzoic acid (0.08 mmol, 40 mol %) in 0.9 mL toluene as the solvent (see Supporting Information File 1). ^f^Malonitrile (0.6 mmol, 3 equiv) was used.

With the acceptable optimized reaction conditions in hand, we next investigated the generality and limitations of this enantioselective conjugate addition reaction. Under the optimized reaction conditions, the conjugate addition reaction of nitromethane (**2**) to a variety of β-silylenones **1** was carried out and the results are summarized in Scheme 2. β-Silylenones bearing electron-donating, electron-withdrawing groups and halogen substituents in the *meta* or *para* position of the phenyl ring reacted smoothly and furnished the desired products **3a–k** in good to excellent yields (71.5–92%) and enantioselectivities (76–97.5% ee). The β-silylenone with a strong electron-withdrawing group (cyano) attached to the phenyl ring, was found to be most reactive as the reaction completed within 4 h and afforded the product **3e** in good yield (88%) and enantioselectivity (95.5% ee). The β-silylenone with a naphthyl substituent also took part in the conjugate addition reaction and gave the corresponding product **3j** in good yield (83%) and enantioselectivity (76% ee). The reaction also tolerated a 2-thienyl-substituted β-silylenone and the desired product **3k** was obtained in good yield (88%) and enantioselectivity (97.5% ee). However, β-silylbutenone **1l** failed to participate in the conjugate addition reaction with nitromethane under the optimized reaction conditions. Pleasingly, using 9-amino-9-deoxyepihydroquinidine (**IX**)–benzoic acid as organocatalyst system (see Supporting Information File 1 for details) promoted the addition reaction and product **3l** was formed in good yield (79%) and excellent enantioselectivity (99% ee). The conjugate addition reaction between malononitrile and β-silylenone **1a** was also investigated using 5 mol % of catalyst **VII** under the optimized reaction conditions. To our delight, the reaction completed within 4 h and the desired product **3m** was isolated in excellent yield (97%) with moderate enantioselectivity (52% ee). β-Silylenone **2n** bearing a *o*-chloro substituent in the aromatic ring remained unreactive under the optimized reaction conditions probably due to steric hindrance.

**Scheme 3:**
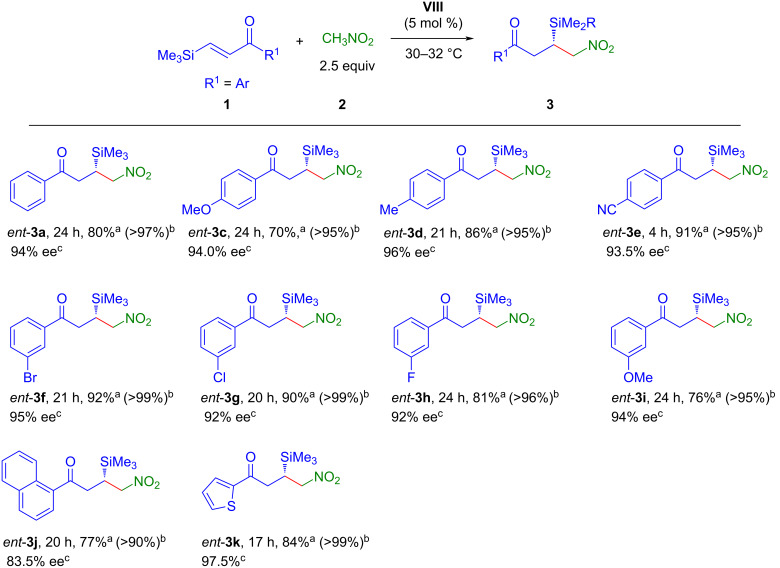
Synthesis of *ent*-**3**. Reaction conditions: **1** (0.2 mmol), **2** (0.5 mmol), catalyst **VIII** (0.01 mmol, 5 mol %) at 30 °C. ^a^Isolated yield of *ent*-**3** after column chromatography. ^b^Conversion in % of the starting material **1** is given in the parentheses, determined by ^1^H NMR analysis of the crude reaction mixture. ^c^Determined by HPLC using chiral stationary phase.

The facile synthesis of both enantiomers of the targeted compounds is of paramount importance since biological activities are dictated by the absolute configuration of the products. To our delight, catalyst **VIII**, the pseudoenantiomeric catalyst of **VII**, allowed to synthesize the enantiomeric products *ent*-**3** (Scheme 3) in high yields and enantioselectivities comparable to the corresponding enantiomers **3** under the optimized reaction conditions. The same set of β-silylenones was explored and an almost similar trend in reactivities, yields as well as enantioselectivities was observed.

**Scheme 4:**
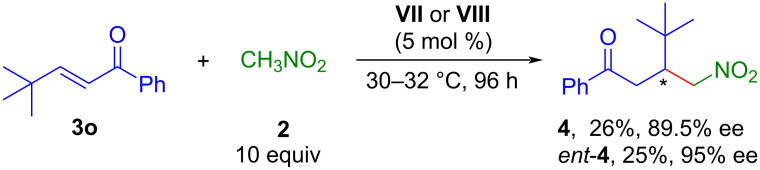
Organocatalytic 1,4-conjuagte addition of nitromethane (**2**) to enone **3o**.

To probe the role of the β-silyl group, the reaction of *tert*-butyl-substituted enone **3o** and nitromethane (**2**) was conducted under the standard reaction conditions using catalyst **VII** or **VIII**, affording only trace amounts of products **4** or *ent*-**4** even after stirring for 48 h [44]. When the same reaction was performed in the presence of 10 equivalents of nitromethane using catalyst **VII**, the product **4** was isolated in 26% yield and 89.5% ee after 96 h whereas the catalyst **VIII** led to *ent*-**4** in 25% yield and 95% ee (Scheme 4). This observation confirmed that the presence of the β-silyl group in the enones played a key role in the high reactivity under the optimized reaction conditions.

**Figure 1:**
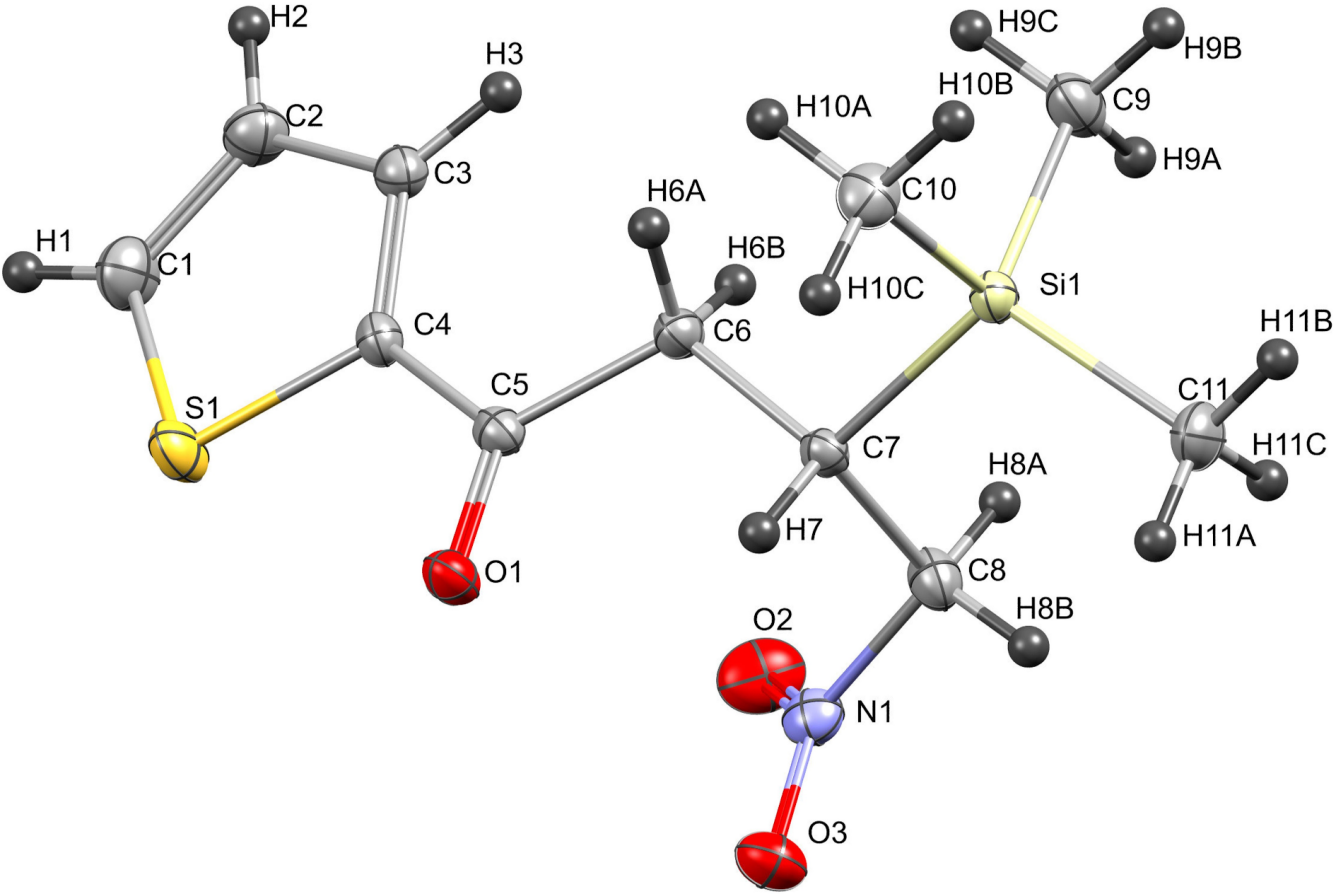
Single crystal X-ray structure of *ent*-**3k** (CCDC 2097263).

The stereochemistry of the silicon-substituted chiral center in compound *ent*-**3k** was found to adopt “(*S*)” configuration which was unambiguously established by single crystal X-ray diffraction analysis (Figure 1) [45].

**Scheme 5:**
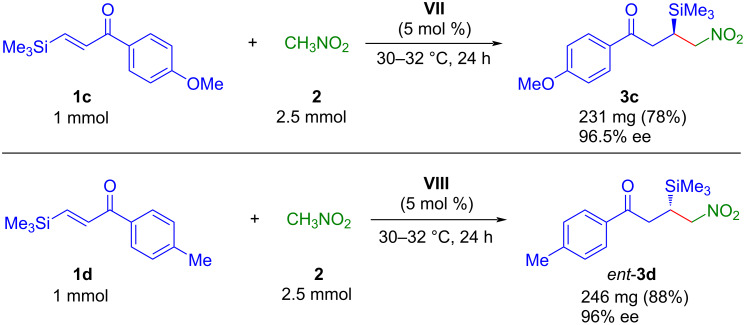
Preparative scale synthesis of **3c** and *ent*-**3d**.

To prove the scalability of this synthetic method, we examined the synthesis of **3c** and *ent*-**3d** in a 1 mmol scale (Scheme 5). The products **3c** and *ent*-**3d** were isolated even with better yields while the enantiomeric excess was unperturbed.

## Conclusion

In summary, we have outlined bifunctional squaramide-catalyzed 1,4-conjugate addition reaction of nitromethane to β-silyl α,β-unsaturated carbonyl compounds to access a series of chiral β-silyl nitroalkanes in high yields and good to excellent enantioselectivities at room temperature. The notable features of this reaction are access to both the (*R*) and (*S*) enantiomers of the products, solvent-free synthesis, mild reaction conditions, low catalyst loading, and use of only a small excess of nitromethane (2.5 equivalents with respect to limiting reagent).

## Supporting Information

File 1 Experimental data and copies of spectra.
