# Supplementary material for: Solvent-free synthesis of enantioenriched β-silyl nitroalkanes under organocatalytic conditions
Source: Beilstein J Org Chem. 2021 Oct 27;17:2642–9. doi: 10.3762/bjoc.17.177 (PMC8561140; doi:10.3762/bjoc.17.177)
Supplement: File 1 — Experimental data and copies of spectra. [file Beilstein_J_Org_Chem-17-2642-s001.pdf]

BEILSTEIN JOURNAL OF ORGANIC CHEMISTRY

Supporting Information

for

Solvent-free synthesis of enantioenriched β-silyl nitroalkanes under organocatalytic conditions

Akhil K. Dubey and Raghunath Chowdhury

Beilstein J. Org. Chem. **2021,** *17,* 2642–2649. doi:10.3762/bjoc.17.177

Experimental data and copies of spectra

License and Terms: This is a supporting information file under the terms of the Creative Commons Attribution License (https://creativecommons.org/licenses/by/4.0). Please note that the reuse, redistribution and reproduction in particular requires that the author(s) and source are credited and that individual graphics may be subject to special legal provisions.
The license is subject to the *Beilstein Journal of Organic Chemistry* terms and conditions: (https://www.beilstein-journals.org/bjoc/terms)

Table of contents

Description	Page
1. General experimental	S2
2. Preparation of catalysts and characterization data	S3
3. Preparation of substrates **1a**–**n**	S3
4. Solvent screening studies	S3
5. Optimization studies for the synthesis of **3l**	S4
6. References	S5
7. General procedure for the preparation of racemic samples	S6
8. General procedure for the preparation of **3a**–**k** and *ent*-**3a**–**k**	S6
9. Characterization data	S7-S17
10. HPLC traces	S18-S55
11. NMR spectra	S56-S94
12. HRMS spectra	S95
13. Single crystal X-ray diffraction analysis of *ent*-**3k**	S96-S105

1. General experimental

Solvent removal was performed with a rotary evaporator that was connected to a dry ice condenser. TLC (0.5 mm) was carried out using Merck TLC plates. Column chromatography was performed on silica gel (230–400 mesh). The ^{1}H and ^{13}C NMR spectroscopic data were recorded with 500 MHz (^{1}H NMR: 500 MHz, ^{13}C NMR: 125 MHz) Varian spectrometer, 600 MHz (^{1}H NMR: 600 MHz, ^{13}C NMR: 150 MHz) Varian spectrometers, or 300 MHz (^{1}H NMR), 800 MHz (^{13}C NMR: 200 MHz) Bruker spectrometers. The ^{1}H and ^{13}C chemical shifts are given in ppm (δ scale) and are measured relative to $CHCl_3$ (7.27 ppm) and $CDCl_3$ (77.0 ppm), respectively. High resolution mass spectra were recorded with an Agilent Advanced Bio

6545XT LC/Q-TOF (MODEL: G6549A, SERIAL NO: SG1917M001). The HRMS data were recorded in the Chemistry department of IIT Bombay as service basis. Enantiomeric excess (ee) values were determined by HPLC analysis with a JASCO (JASCOPU-2080) instrument fitted with a Daicel Chiralpak AD-H column, Daicel Chiralcel OD-H column and Daicel Chiralpak OJ-H with UV-2075 detector (λ fixed at 254 nm or 220 nm). Melting points (mp) were measured on a Büchi B-540 apparatus. ATR IR spectra were recorded using a Bruker tensor II. All the solvents were distilled prior to use. Nitromethane was purchased from commercial sources and used without any purification or drying. The single-crystal X-ray diffraction data of *ent*-**3k** were collected using a Rigaku Saturn 724+ /Dual Source (Cu and MoKα) Single Crystal X-ray diffractometer by using a MoKα radiation (λα = 0.71073 Å) at 100 K. The structures were solved by SHELXT and refined with SHElXL using the Olex2 program.

2. Preparation of catalysts I–XII

The catalysts **I**,[1] **II**,[2] **III**,[1] **IV**, [1] **V**,[3] **VI**,[4] **VII**,[5] **VIII**,[5] and **IX-XII**[1] were prepared following the precedent literature procedures. For characterization data, see our previous report. [3]

3. Preparation of β-silyl enones 1a–n

Enones **1a–k** and **1n** were synthesized according to previous reports. [5] **1l**[6] and **1o**[7] were prepared following the reported procedure.

4. Table S1: Solvent screening[a]

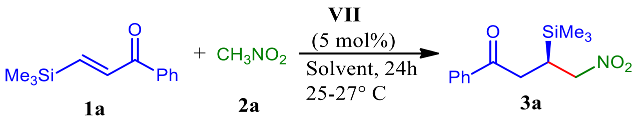

Entry	Solvent (mL)	**2a**(equiv)	[%] of Conv.[b] of **1a**	[%]ee[c] of **3a**
1	Mesitylene (0.2)	10	90	97.5
2	$C_6H_5CF_3$ (0.2)	10	90	96
3	MTBE (0.2)	10	70	97.5
4	THF (0.2)	10	70	97.5
5	CH_3CN (0.2)	10	75	96.5
6	DCM (0.2)	10	92	97.5

[a]Reaction conditions: **1a** (0.2 mmol), **2a** (2 mmol), catalyst (0.01 mmol, 5mol%) in solvent 0.2 mL. [b]% of conversion of the starting material **1a**, determined by ^{1}H NMR analysis of the crude reaction mixture. [c]Determined by HPLC using chiralpak OD-H column.

5. Table S2: Optimization studies for the synthesis of **3l**[a]

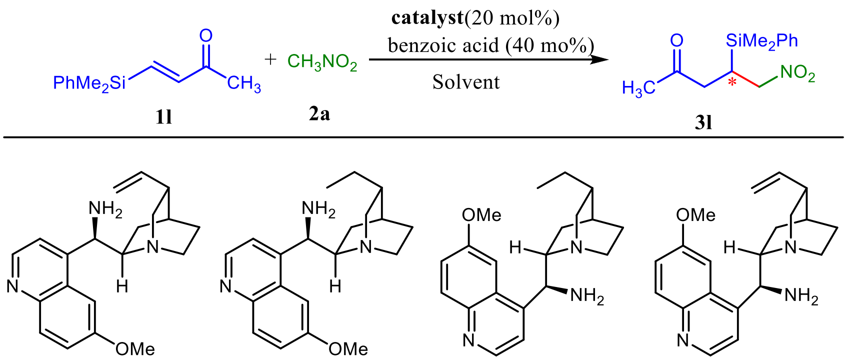

Entry	Cat.	Solvent (mL)	**2a**(equiv)	Temp (°C)	[%][b] yield of **3l**	[%]ee[c] of **3l**
1	**IX**	CH_3NO_2 (1.0)	NA	31	79	92
2	**X**	CH_3NO_2 (1.0)	NA	31	78	97
3	**XI**	CH_3NO_2 (1.0)	NA	31	79	-92
4	**X**	Toluene (0.9)	10	26	79	99
5	**X**	THF(0.9)	10	26	60	97
6	**X**	$C_6H_5CF_3$ (0.9)	10	26	68	98
7	**X**	H_2O (0.9)	10	26	72	97
8	**X**	No solvent	10	26	62	96
9	**XII**	No solvent	10	26	78	-96

[a]Reaction conditions: **1l** (0.2 mmol), **2a** (2 mmol), catalyst (20 mol%), benzoic acid (40 mol%). [b] Isolated yield after column chromatography. [c]Determined by HPLC using chiralpak OD-H column.

General procedure for the preparation of 3l

All the reactions were carried out in normal solvents and no special precautions were taken to exclude air and moisture from the reaction flask. In a 5 mL round-bottomed flask, benzoic acid (≈ 9.8 mg, 0.08 mmol, ≈ 40 mol %) was added to the catalyst (13 mg, 0.04 mmol, 20 mol %) followed by the solvent. The resulting mixture was stirred for 5 min. After that, nitromethane (105 µL, 2 mmol, 10 equiv) was added and the reaction mixture was stirred for 2 d. The reaction mixture was directly subjected to column chromatography. In case of water as a solvent, the reaction mixture was extracted with dichloromethane (3 × 10 mL) and the combined extract was washed with brine, dried ($MgSO_4$), and evaporated. The residue was purified by column chromatography on silica gel (5% petroleum ether/EtOAc to 10% petroleum ether/EtOAc) to afford the corresponding products **3l** as colourless liquid.

6. References:

1. B. Vakulya, S. Varga, A. Csámpai, T. Soós, *Org. Lett.* **2005**, *7*, 1967.
2. K. Greenaway, P. Dambruoso, A. F. Andrew, J. Hazelwood, F. Sladojevich, D. J. Dixon, *Synthesis*, **2011**, 1880.
3. R. Chowdhury, A. Dubey and S. K. Ghosh, *Eur. J. org. Chem.* **2020**, 2962.
4. K. Bera, I.I.N. Namboothiri, *Org. Lett.* **2012**, *14*, 980.
5. Y. Zhang, J. Huang, Y. Guo, L. Li, Z. Fu, W. Huang, *Angew. Chem. Int. Ed.* **2018**, *57*, 4594.
6. I. Fleming, T. W. Newton, V. Sabin, F. Zammattio, *Tetrahedron*, **1992**, *48*, 7793-7802.
7. J. Humbrías-Martín, M. C. Pérez-Aguilar, R. Mas-Ballesté, A.D. Litta, A. Lattanzi, G. D. Sala, J. A. Fernández-Salas, J. Alemán, *Adv. Synth. Catal.***2019**, *361*, 4790.

7. General procedure for the preparation of *racemic* product *rac*-3a-3c, *rac*-3e, *rac*-3g, *rac*-3i-3k

In an oven-dried 5 mL round-bottomed flask, β-silylenone **1** was taken in 0.4 mL of toluene. Then, nitromethane (105 µL, 2 mmol, 10 equiv) was added followed by DBU (20 mol %). The resulting reaction mixture was stirred for 24 h at 30–32 °C. The reaction mixture was directly subjected to column chromatography on silica gel (petroleum ether/EtOAc) to afford the corresponding racemic product.

(It should be noted that the yields and reaction time were not optimized for the above reactions). Racemic samples (for ***rac*-3d**, ***rac*-3f**, ***rac*-3h** and ***rac*-3l)** were prepared by mixing both enantiomers of the respective product.

8. General procedure for the preparation of 3a–k and *ent*-**3a**–**k**

β-Silylenone **1** (0.2 mmol, 1 equiv) and catalyst **VII** (6.3 mg, 0.01mmol, 5 mol %) or catalyst **VIII** (6.3 mg, 0.01mmol, 5 mol %) were taken in a screw cap 2 mL glass vial equipped with a magnetic stirring bar. Nitromethane (27 µl, ≈ 0.5 mmol, 2.5 equiv) was added and the reaction mixture stirred for 4–24 h. Once the β-silylenone **1** was consumed (monitored by TLC/^{1}H NMR), the reaction mixture was directly subjected to column chromatography on silica gel (petroleum ether/EtOAc) to afford the product **3a**–**k** and *ent*-**3a**–**k**.

9. Characterization data

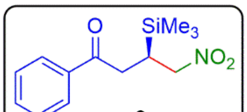

3a

(*R*)-4-Nitro-1-phenyl-3-(trimethylsilyl)butan-1-one (**3a**)

The product was isolated as a light-yellow liquid, yield = 43.5 (82%). **^{1}H NMR (500 MHz, CDCl$_3$)**: δ 7.94 (d, *J* = 7.3 Hz, 2 H), 7.58 (t, *J* = 7.4 Hz, 1 H), 7.48 (t, *J* = 7.7 Hz, 2 H), 4.54 (d, *J* = 7.0 Hz, 2 H), 3.21 (dd, *J* = 18.4, 6.4 Hz, 1 H), 3.15 (dd, *J* = 18.4, 5.4 Hz, 1 H), 2.25 (pent, *J* = 6.5 Hz 1 H), 0.11 (s, 9 H); 1**3C{1H} NMR (125 MHz, CDCl$_3$)**: δ 198.3, 136.6, 133.3, 128.7 (2 C), 127.9 (2 C), 77.4, 36.4, 21.6, -2.47 (3 C); **IR** (ATR): 2954, 2899, 1685, 1546, 1448, 1374, 1251, 1224, 993, 837 cm^{-1}; **HRMS (ESI)** calcd for $C_{13}H_{19}NNaO_3Si$ [M + Na]$^+$: 288.1023, found: 288.1022; **HPLC**: The ee was determined by HPLC using a Daicel Chiralcel OD-H [*n*-hexane/*i*-PrOH (97/3)]; flow rate 1.0 mL/min; λ = 220 nm; τ_{major} = 7.39 min, τ_{minor} = 9.03 min; $[\alpha]_D^{24}$ = -17.5 (*c* 1.5, $CHCl_3$, ee = 97%).

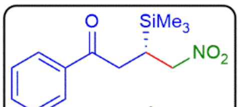

ent-3a

(*S*)-4-Nitro-1-phenyl-3-(trimethylsilyl)butan-1-one (***ent*-3a**)

The product was isolated as a light-yellow liquid, yield = 42.5 mg (80%). **HPLC**: The ee was determined by HPLC using a Daicel Chiralcel OD-H [*n*-hexane/*i*-PrOH (97/3)]; flow rate 1.0 mL/min; λ = 220 nm; τ_{minorr} = 8.23 min, τ_{major} = 9.48 min; $[\alpha]_D^{24}$ = +16.0 (*c* 1.96, $CHCl_3$, ee = 94%).

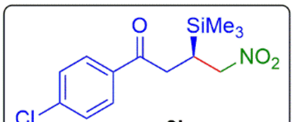

3b

(*R*)-1-(4-Chlorophenyl)-4-nitro-3-(trimethylsilyl)butan-1-one (**3b**)

The product was isolated as a light-yellow liquid, yield = 55.2 (92%). **^{1}H NMR (500 MHz, CDCl$_3$)**: δ 7.88 (d, J = 8.5 Hz, 2 H), 7.44 (d, J = 8.6 Hz, 2 H), 4.54-4.49 (m, 2 H), 3.18 (dd, J = 18.3, 6.4 Hz, 1 H), 3.10 (dd, J = 18.3, 5.4 Hz, 1 H), 2.26-2.21 (m, 1 H), 0.11 (s, 9 H); 1**3C{1H} NMR (125 MHz, CDCl$_3$)**: δ 197.1, 139.8, 134.9, 129.4 (2 C), 129.0 (2 C), 77.3, 36.4, 21.6, -2.50 (3 C); **IR** (ATR): 2954, 2899, 1685, 1547, 1374, 1251, 1221, 992, 837 cm^{-1}; **HRMS (ESI)** calcd for $C_{13}H_{18}ClNNaO_3Si$ [M + Na]$^+$: 322.0635, found: 322.0634; $[\alpha]_D^{24}$ = -14.9 (c 1.55, $CHCl_3$); **HPLC**: enantiomers could not be separated by AD-H, OD-H, OJ-H and AS-H columns.

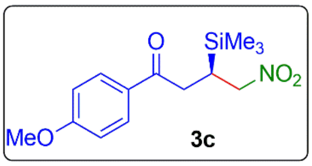

(*R*)-1-(4-Methoxyphenyl)-4-nitro-3-(trimethylsilyl)butan-1-one (3c)

The product was isolated as a colourless liquid, yield = 43.2 mg (73%). **^{1}H NMR (500 MHz, CDCl$_3$)**: δ 7.92 (d, J = 8.8 Hz, 2 H), 6.93 (d, J = 8.9 Hz, 2 H), 4.53 (d, J = 6.8 Hz, 2 H), 3.87 (s, 3 H), 3.15 (dd, J = 18.0, 6.4 Hz, 1 H), 3.08 (dd, J = 18.0, 5.5 Hz, 1 H), 2.22 (*pent*, J = 6.5 Hz, 1 H), 0.10 (s, 9 H); 1**3C{1H} NMR (125 MHz, CDCl$_3$)**: δ 196.8, 163.6, 130.2 (2 C), 129.6, 113.8 (2 C), 77.4, 55.5, 35.9, 21.7, -2.46 (3 C); **IR** (ATR): 3020, 2956, 2902, 1600, 1548, 1375, 1252, 1170, 835, 747 cm^{-1}; **HRMS (ESI)** calcd for $C_{14}H_{22}NO_4Si$ [M + H]$^+$: 296.1309, found: 296.1309; **HPLC**: The ee was determined by HPLC using a Daicel Chiralpak AD-H [*n*-hexane /*i*-PrOH (97/3)]; flow rate 1.0 mL/min; λ = 220 nm; τ_{minor} = 13.40 min, τ_{major}= 14.88 min; $[\alpha]_D^{24}$ = -16.5 (c 2.23, $CHCl_3$, ee = 97%).

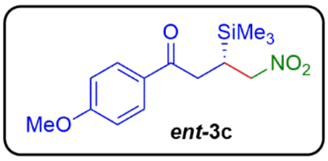

(*S*)-1-(4-Methoxyphenyl)-4-nitro-3-(trimethylsilyl)butan-1-one (*ent*-3c)

The product was isolated as a colorless liquid, yield = 41.5 mg (70%). **HPLC**: The ee was determined by HPLC using a Daicel Chiralpak AD-H [*n*-hexane/*i*-PrOH (97/3)]; flow rate 1.0

mL/min; λ = 220 nm; τ_{major} = 13.55 min, τ_{minor}= 15.07 min; $[\alpha]_D^{24}$ = +18.2 (*c* 2.11, $CHCl_3$, ee = 94%).

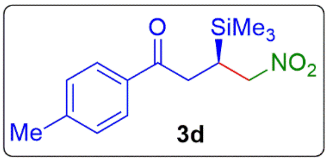

(*R*)-4-Nitro-1-(*p*-tolyl)-3-(trimethylsilyl)butan-1-one (**3d**)

The product was isolated as slightly yellow liquid, yield = 44.8 mg (80%). **^{1}H NMR (300 MHz, $CDCl_3$)**: 7.85 (d, *J* = 8.9 Hz, 2 H), 7.29 (d, *J* = 8.0 Hz, 2 H), 4.54 (d, *J* = 7.0 Hz, 2 H), 3.20 (dd, *J* = 18.3, 6.1Hz, 1 H), 3.12 (dd, *J* = 18.2, 5.4 Hz, 1 H), 2.43 (s, 3 H), 2.24 (*pent*, *J* = 6.4 Hz, 1 H), 0.12 (s, 9 H); 1**3C{1H} NMR (200 MHz, $CDCl_3$)**: *δ* 198.0, 144.1, 134.1, 129.3 (2 C), 128.1 (2 C), 77.4, 36.2, 21.7, 21.6, -2.5 (3 C); **IR** (ATR): 2954, 1680, 1547, 1375, 1251, 839, 750 cm^{-1}; **HRMS (ESI)** calcd for $C_{14}H_{21}NNaO_3Si$ $[M + Na]^+$: 302.1183, found: 302.1186; **HPLC**: The ee was determined by HPLC using a Daicel Chiralpak AD-H [*n*-hexane/*i*-PrOH (97/3)]; flow rate 0.5 mL/min; λ = 220 nm; τ_{minor} = 14.10 min, τ_{major}= 15.50 min; $[\alpha]_D^{24}$ = -19.0 (*c* 2.16, $CHCl_3$, ee = 92.5%).

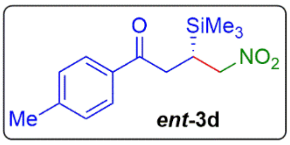

(*S*)-4-Nitro-1-(*p*-tolyl)-3-(trimethylsilyl)butan-1-one (***ent*-3d**)

The product was isolated as slightly yellow liquid, yield = 48.1 mg (86%). **HPLC**: The ee was determined by HPLC using a Daicel Chiralpak AD-H [*n*-hexane/*i*-PrOH (97/3)]; flow rate 0.5 mL/min; λ = 220 nm; τ_{major} = 13.63 min, τ_{minor} = 15.08 min; $[\alpha]_D^{24}$ = +15.1 (*c* 2.01, $CHCl_3$, ee = 96%).

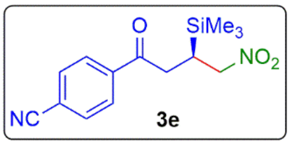

(*R*)-4-(4-Nitro-3-(trimethylsilyl)butanoyl)benzonitrile (3e)

The product was isolated as a light-yellow liquid, yield = 51mg (88%).**^{1}H NMR (500 MHz, CDCl$_3$)**: δ 8.02 (d, *J* = 8.4 Hz, 2 H), 7.77 (d, *J* = 8.4 Hz, 2 H), 4.55-4.47 (m, 2 H), 3.22 (dd, *J* = 18.6, 6.5 Hz, 1 H), 3.12 (dd, *J* = 18.5, 5.3 Hz, 1 H), 2.27-2.22 (m, 1 H), 0.11 (s, 9 H); 1**3C{1H} NMR (125 MHz, CDCl$_3$)**: δ 197.1, 139.5, 132.5 (2 C), 128.4 (2 C), 117.8, 116.5, 77.2, 36.8, 21.5, -2.59 (3 C); **IR** (ATR): 2951, 2919, 2228, 1689, 1552, 1373, 1327, 1249, 1059, 998, 829 cm^{-1}; **HRMS (ESI)** calcd for $C_{14}H_{18}N_2NaO_3Si$ [M + Na]$^+$: 313.0975, found: 313.0975; **HPLC**: The ee was determined by HPLC using a Daicel Chiralcel OD-H [*n*-hexane/*i*-PrOH (95/5)]; flow rate 1.0 mL/min; λ = 220 nm; τ_{minor} = 28.52 min, τ_{major}= 30.58 min; $[\alpha]_D^{22}$ = -16.4 (*c* 1.33, CHCl$_3$, ee = 95.5%).

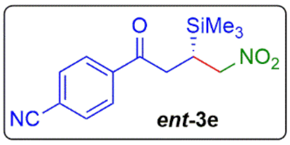

(*S*)-4-(4-Nitro-3-(trimethylsilyl)butanoyl)benzonitrile (*ent*-3e)

The product was isolated as a light-yellow liquid, yield = 53mg (91%). **HPLC**: The ee was determined by HPLC using a Daicel Chiralcel OD-H [*n*-hexane/*i*-PrOH (97/5)]; flow rate 1.0 mL/min; λ = 220 nm; τ_{major} = 30.47 min, τ_{minor}= 34.78 min; $[\alpha]_D^{22}$ = +17.5 (*c* 2.30, CHCl$_3$, ee = 93.5%).

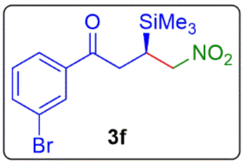

(*R*)-1-(3-Bromophenyl)-4-nitro-3-(trimethylsilyl)butan-1-one (3f)

The product was isolated as a light-yellow liquid, yield = 62.7mg (91%). **^{1}H NMR (500 MHz, CDCl$_3$)**: δ 8.06 (s, 1 H), 7.86 (d, *J* = 7.8 Hz, 1 H), 7.70 (d, *J* = 7.9 Hz, 1 H), 7.36 (t, *J* = 7.9 Hz, 1 H), 4.55-4.48 (m, 2 H), 3.18 (dd, *J* = 18.5, 6.3 Hz, 1 H), 3.11 (dd, *J* = 18.5, 5.3 Hz, 1 H), 2.26-2.21 (m, 1 H), 0.11 (s, 9 H); 1**3C{1H} NMR (125 MHz, CDCl$_3$)**: δ 197.0, 138.3, 136.2,

131.0, 130.3, 126.5, 123.0, 77.3, 36.6, 21.6, -2.5 (3 C); **IR** (ATR): 2954, 2899, 1688, 1547, 1416, 1250, 1213, 838, 775 cm^{-1}; **HRMS (ESI)** calcd for $C_{13}H_{18}BrNNaO_3Si$ [M + Na]$^+$: 368.0107, found: 368.0106; **HPLC**: The ee was determined by HPLC using a Daicel Chiralcel OD-H [*n*-hexane/*i*-PrOH (97/3)]; flow rate 1.0 mL/min; λ = 220 nm; τ_{major} = 9.25 min, τ_{minor}= 10.32 min; $[\alpha]_D^{22}$ = -15.1 (*c* 1.42, $CHCl_3$, ee = 91%).

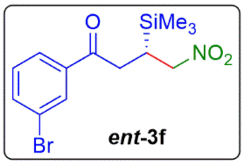

(*S*)-1-(3-Bromophenyl)-4-nitro-3-(trimethylsilyl)butan-1-one (***ent*-3f**)

The product was isolated as a colorless liquid, yield = 63.4 mg (92%). **HPLC**: The ee was determined by HPLC using a Daicel Chiralcel OD-H [*n*-hexane/*i*-PrOH (97/3)]; flow rate 1.0 mL/min; λ = 220 nm; τ_{minor} = 9.32 min, τ_{major}= 10.38 min; $[\alpha]_D^{22}$ = +10.2 (*c* 1.46, $CHCl_3$, ee = 95%).

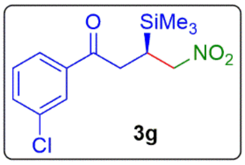

(*R*)-1-(3-Chloroophenyl)-4-nitro-3-(trimethylsilyl)butan-1-one (**3g**)

The product was isolated as a liquid, yield = 52.8 mg (88%). **^{1}H NMR (500 MHz, $CDCl_3$)**: *δ* 7.90 (s, 1 H), 7.82 (d, *J* = 7.8 Hz, 1 H), 7.55 (d, *J* = 8.0 Hz, 1 H), 7.42 (t, *J* = 7.9 Hz, 1 H), 4.55-4.48 (m, 2 H), 3.18 (dd, *J* = 18.5, 6.4 Hz, 1 H), 3.11 (dd, *J* = 18.5, 5.4 Hz, 1 H), 2.26-2.21 (m, 1 H), 0.11 (s, 9 H); 1**3C{1H} NMR (125 MHz, $CDCl_3$)**: *δ* 197.1, 138.1, 135.0, 133.3, 130.0, 128.1, 126.1, 77.3, 36.6, 21.6, -2.48 (3 C); **IR** (ATR): 3024, 2955, 1689, 1547, 1420, 1374, 1252, 1216, 998, 839, 752 cm^{-1}; **HRMS (ESI)** calcd for $C_{13}H_{18}ClNNaO_3Si$ [M + Na]$^+$: 322.0634, found: 322.0634; **HPLC**: The ee was determined by HPLC using a Daicel Chiralcel OD-H [*n*-hexane

/*i*-PrOH (97/3)]; flow rate 0.5 mL/min; λ = 220 nm; τ_{major} = 17.58 min, τ_{minor}= 19.68 min; $[\alpha]_D^{23}$ = -12.6 (*c* 1.86, $CHCl_3$, ee = 92.5%).

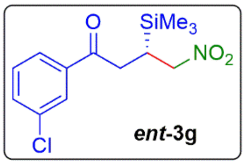

(*S*)-1-(3-Chloroophenyl)-4-nitro-3-(trimethylsilyl)butan-1-one (***ent*-3g**)

The product was isolated as a liquid, yield = 54 mg (90%). **HPLC**: The ee was determined by HPLC using a Daicel Chiralcel OD-H [*n*-hexane/*i*-PrOH (97/3)]; flow rate 0.5 mL/min; λ = 220 nm; τ_{minor} = 17.27 min, τ_{major} = 19.17 min; $[\alpha]_D^{23}$ = +12.9 (*c* 1.59, $CHCl_3$, ee = 92 %).

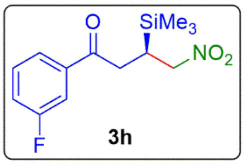

(*R*)-1-(3-Fluorophenyl)-4-nitro-3-(trimethylsilyl)butan-1-one (**3h**)

The product was isolated as a colorless liquid, yield = 43.2 mg (76%). **^{1}H NMR (600 MHz, $CDCl_3$)**: *δ* 7.72 (d, *J* = 7.7 Hz, 1 H), 7.64-7.60 (m, 1 H), 7.49-7.42 (m, 1 H), 7.31-7.25 (m, 1 H), 4.58-4.47 (m, 2 H), 3.20 (dd, *J* = 18.5, 6.3 Hz, 1 H), 3.10 (dd, *J* = 18.5, 5.5 Hz, 1 H), 2.28-2.19 (m, 1 H), 0.11 (s, 9 H); **13C{1H} NMR (200 MHz, $CDCl_3$)**: *δ* 197.1, 162.8 (d, J_{C-F} = 47.0 Hz), 138.6, 130.4 (d, J_{C-F} = 7.0 Hz), 123.7, 120.3 (d, J_{C-F} = 21.3 Hz), 114.7 (d, J_{C-F} = 22.2 Hz), 77.3, 36.6, 21.6, -2.5 (3 C); **IR** (ATR): 2956, 1689, 1548, 1251, 839, 748 cm^{-1}; **HRMS (ESI)** calcd for $C_{13}H_{18}FNNaO_3Si$ $[M + Na]^+$: 306.0929, found: 306.0929; **HPLC**: The ee was determined by HPLC using a Daicel Chiralcel OD-H [*n*-hexane/*i*-PrOH (97/3)]; flow rate 0.5 mL/min; λ = 220 nm; τ_{major} = 14.89 min, τ_{minor} = 15.87 min; $[\alpha]_D^{25}$ = -17.43 (*c* 2.35, $CHCl_3$, ee = 92.5%).

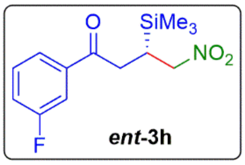

(*S*)-1-(3-Fluorophenyl)-4-nitro-3-(trimethylsilyl)butan-1-one (***ent*-3h**)

The product was isolated as a colourless liquid, yield = 46 mg (81%). **HPLC**: The ee was determined by HPLC using a Daicel Chiralcel OD-H [*n*-hexane/*i*-PrOH (97/3)]; flow rate 0.5 mL/min; λ = 220 nm; τ_{minor}= 14.88 min, τ_{major} = 15.81 min; $[\alpha]_D^{26}$ = -14.54 (*c* 1.64, $CHCl_3$, ee = 92%).

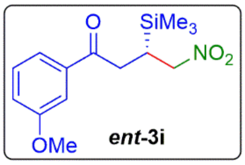

(*R*)-1-(3-Methoxyphenyl)-4-nitro-3-(trimethylsilyl)butan-1-one (**3i**)

The product was isolated as light-yellow liquid, yield = 42.3 mg (71.5%). **^{1}H NMR (300 MHz, $CDCl_3$)**: *δ* 7.54-7.48 (m, 2 H), 7.39 (t, *J* = 7.9 Hz, 1 H), 7.14 (dd, *J* = 8.0, 2.0 Hz, 1 H), 4.54 (*J* = 8.0 Hz, 2 H), 3.87 (s, 3 H), 3.21 (dd, *J* = 18.3, 6.2 Hz, 1 H), 3.14 (dd, *J* = 18.3, 5.5 Hz, 1 H), 2.25 (*pent*, *J* = 6.5 Hz, 1 H), 0.12 (s, 9 H); 1**3C{1H} NMR (200 MHz, $CDCl_3$)**: *δ* 198.2, 159.9, 137.9, 129.7, 120.6, 119.8, 112.3, 77.4, 55.5, 36.5, 21.7, -2.5 (3 C); **IR** (ATR): 2956, 1684, 1548, 1256, 839, 748 cm^{-1}; **HRMS (ESI)** calcd for $C_{14}H_{22}NO_4Si$ $[M + H]^+$: 296.1310, found: 296.1310; The ee was determined by HPLC using a Daicel Chiralcel OD-H [*n*-hexane/*i*-PrOH (95/5)]; flow rate 0.5 mL/min; λ = 220 nm; τ_{major} = 17.45 min, τ_{minor} = 26.4 min; $[\alpha]_D^{23}$ = -14.2 (*c* 2.71, $CHCl_3$, ee = 96.5%).

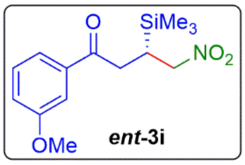

(*S*)-1-(3-Methoxyphenyl)-4-nitro-3-(trimethylsilyl)butan-1-one (***ent*-3i**)

The product was isolated as light-yellow liquid, yield = 44.8 mg (76%). **HPLC**: The ee was determined by HPLC using a Daicel Chiralcel OD-H [*n*-hexane/*i*-PrOH (95/5)]; flow rate 0.5 mL/min; λ = 220 nm; = τ_{minor} 17.90 min, τ_{major} = 27.32 min; $[\alpha]_D^{26}$ = +14.9 (*c* 2.32, $CHCl_3$, ee = 94%).

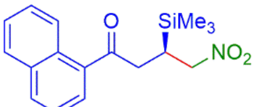

3j

(*R*)-1-(Naphthalen-1-yl)-4-nitro-3-(trimethylsilyl)butan-1-one (**3j**)

The product was isolated as a liquid, yield = 52.4 mg (83%). **^{1}H NMR (500 MHz, CDCl$_3$)**: δ 8.55 (d, *J* = 8.6 Hz, 1 H), 8.00 (d, *J* = 8.2 Hz, 1 H), 7.88 (d, *J* = 7.4 Hz, 2 H), 7.61(td, *J* = 7.1, 1.1 Hz, 1 H), 7.56-7.49 (m, 2 H), 4.62-4.56 (m, 2 H), 3.31 (dd, *J* = 18.6, 6.5 Hz, 1 H), 3.23 (dd, *J* = 18.6, 5.2 Hz, 1 H), 2.35-2.30 (m, 1 H), 0.16 (s, 9 H); 1**3C{1H} NMR (125 MHz, CDCl$_3$)**: δ 202.3, 135.5, 134.0, 132.8, 130.0, 128.4, 128.0, 127.5, 126.5, 125.6, 124.3, 77.4, 39.8, 22.0, -2.46 (3 C); **IR** (ATR): 3020, 2954, 2897, 1681, 1546, 1374, 1251, 1176, 839, 751cm^{-1}; **HRMS (ESI)** calcd for $C_{17}H_{22}NO_3Si$ $[M + H]^+$: 316.1360, found: 316.1360; **HPLC**: The ee was determined by HPLC using a Daicel Chiralpak AD-H [*n*-hexane /*i*-PrOH (97/3)]; flow rate 1.0 mL/min; λ = 220 nm; τ_{major} = 8.53 min, τ_{minor}= 10.05 min; $[\alpha]_D^{23}$ = -18.5 (*c* 1.64, CHCl$_3$, ee = 76%).

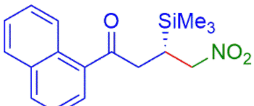

***ent*-3j**

(*S*)-1-(Naphthalen-1-yl)-4-nitro-3-(trimethylsilyl)butan-1-one (***ent*-3j**)

The product was isolated as a colourless liquid, yield = 48.6 mg (77%). **HPLC**: Daicel Chiralpak AD-H [*n*-hexane /*i*-PrOH (97/3)]; flow rate 1.0 mL/min; λ = 220 nm; τ_{minor} = 8.40 min, τ_{major} = 9.84 min; $[\alpha]_D^{23}$ = +16.5 (*c* 2.00, CHCl$_3$, ee = 83.5 %).

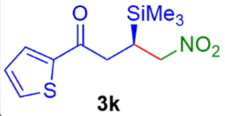

3k

(*R*)-4-nitro-1-(thiophen-2-yl)-3-(trimethylsilyl)butan-1-one (**3k**)

The product was isolated as a light-yellow liquid. yield = 47.8 mg (88%). **^{1}H NMR (500 MHz, $CDCl_3$)**: *δ* 7.72 (d, *J* = 3.8 Hz, 1 H), 7.65 (d, *J* = 4.9 Hz, 1 H), 7.14 (t, *J* = 4.0 Hz, 1 H), 4.56-4.51 (m, 2 H), 3.15 (dd, *J* = 17.8, 6.5 Hz, 1 H), 3.09 (dd, *J* = 17.7, 5.5 Hz, 1 H), 2.22-2.17 (m, 1 H), 0.12 (s, 9 H); 1**3C{1H} NMR (125 MHz, $CDCl_3$)**: *δ* 191.2, 143.6, 133.9, 132.0, 128.2, 77.2, 37.0, 22.0, -2.5 (3 C); **IR** (ATR): 3022, 2955, 1662, 1548, 1415, 1374, 1252, 1215, 839, 747 cm^{-1}; **HRMS (ESI)** calcd for $C_{11}H_{18}NO_3SSi$ $[M + H]^+$: 272.0700, found: 272.0700; **HPLC**: The ee was determined by HPLC using a Daicel Chiralcel OJ-H [*n*-hexane/*i*-PrOH (97/3)]; flow rate 1.0 mL/min; λ = 254 nm; τ_{minor} = 34.90 min, τ_{major}= 38.53 min; $[\alpha]_D^{23}$ = -16.0 (*c* 1.41, $CHCl_3$, ee = 97.5%).

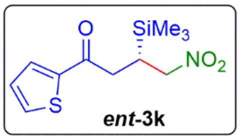

(*S*)-4-Nitro-1-(thiophen-2-yl)-3-(trimethylsilyl)butan-1-one (***ent*-3k**)

The product was isolated as a liquid which solidify on standing in refrigerator, yield = 45.6 mg (84%). Mp. 58-59 °C (petroleum ether). **HPLC**: The ee was determined by HPLC using a Daicel Chiralcel OJ-H [*n*-hexane/*i*-PrOH (97/3)] flow rate 1.0 mL/min; λ = 254 nm; τ_{major} = 34.62 min, τ_{minor}= 42.2 min; $[\alpha]_D^{23}$ = +17.32 (*c* 2.25, $CHCl_3$, ee = 97.5%).

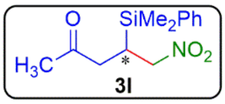

Asymmetric synthesis of 3l

In a 5 mL round-bottomed flask, benzoic acid (≈ 9.8 mg, 0.08 mmol, ≈ 40 mol %) was added to the catalyst **IX** (13 mg, 0.04 mmol, 20 mol %) followed by the solvent. The resulting mixture was stirred for 5 min. After that, nitromethane (105 µL, 2 mmol, 10 equiv) was added and the reaction mixture was stirred for 2 d. The reaction mixture was directly subjected to column chromatography on silica gel (5% petroleum ether/EtOAc to 10% petroleum ether/EtOAc) to

afford the corresponding products **3l** as colourless liquid (42 mg, 79% yield). **^{1}H NMR (500 MHz, CDCl$_3$)**: δ 7.49 (dd, J = 7.3, 1.5 Hz, 2 H), 7.42-7.37 (m, 3 H), 4.42 (dd, J = 12.3, 4.0 Hz, 1 H), 4.35 (dd, J = 12.3, 10.4 Hz, 1H), 2.58 (d, J = 5.9 Hz, 1 H), 2.33-2.26 (m, 1H), 2.07 (s, 3 H), 0.39 (s, 3 H), 0.37 (s, 3 H); **13C{1H} NMR (125 MHz, CDCl$_3$)**: δ 206.6, 135.4, 133.8 (2 C), 129.8, 128.2 (2 C), 77.1, 41.2, 29.9, 21.2, -4.31, -4.32; **IR** (ATR): 3024, 2957, 1714, 1547, 1370, 1255, 1171, 1112, 814, 770, 737 cm^{-1}; **HRMS (ESI)** calcd for $C_{13}H_{19}NNaO_3Si$ [M + Na]$^+$: 288.1023, found: 288.1023; The ee was determined by HPLC using a Daicel Chiralcel OJ-H [*n*-hexane/*i*-PrOH (90/10)]; flow rate 1.0 mL/min; λ = 220 nm; τ_{minor} = 20.98 min, τ_{major}= 22.55 min; $[\alpha]_D^{25}$ = +25.5 (*c* 1.15, CHCl$_3$, ee = 99%).

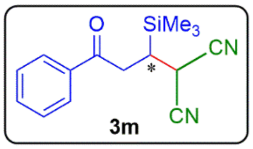

3m

Asymmetric synthesis of 3m

β-Silylenone **1a** (0.2 mmol, 1 equiv) and catalyst **VII** (6.3 mg, 0.01mmol, 5 mol %) were taken in a screw cap 2 mL glass vial equipped with a magnetic stirring bar. Malononitrile (33 µL, ≈ 0.6 mmol, 3 equiv) was added and the reaction mixture stirred for 4 h at 30 °C. Once the β-silylenone **1** was consumed (monitored by TLC/^{1}HNMR), the reaction mixture was directly subjected to column chromatography on silica gel (petroleum ether/EtOAc) to afford the product **3m** (52.5, 97% yield) as colourless liquid. **^{1}H NMR (600 MHz, CDCl$_3$)**: δ 7.98 (dd, J = 7.74, 1.15 Hz, 2 H), 7.63 (t, J = 7. 4 Hz, 1 H), 7.52-7.49 (m, 2 H), 4.31 (d, J = 6.3 Hz, 1 H), 3.35 (dd, J = 18.8, 5.5 Hz, 1 H), 3.30 (dd, J = 16.6, 6.8 Hz, 1 H), 2.11-2.07 (m, 1 H), 0.26 (s, 9 H); 1**3C{1H} NMR (200 MHz, CDCl$_3$)**: δ 197.7, 135.8, 133.9, 128.8 (2 C), 128.0 (2 C), 113.2, 113.0, 36.6, 23.5, 23.4, -1.8 (3 C); **IR** (ATR): **HRMS (ESI)** calcd for $C_{15}H_{18}N_2NaOSi$ [M + Na]$^+$: 293.1078, found: 293.1077; **HPLC**: The ee was determined by HPLC using a Daicel

Chiralcel OD-H [*n*-hexane/*i*-PrOH (95/5)]; flow rate 1.0 mL/min; λ = 220 nm; τ_{major} = 13.40 min, τ_{minor} = 16.47 min; $[\alpha]_D^{23}$ = -19.1 (*c* 1.61, $CHCl_3$, ee = 52%).

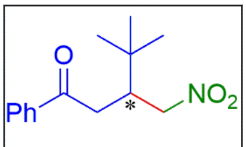

Chiral **4**

Asymmetric synthesis of 4

(*E*)-4,4-Dimethyl-1-phenylpent-2en-1-one (**1o**, 0.2 mmol, 1 equiv) and catalyst **VII** (6 mg, 0.01 mmol, 5 mol %) or catalyst **VIII** (6.3 mg, 0.01 mmol, 5 mol %) were taken in a screw cap 4 mL glass vial equipped with a magnetic stirring bar. Nitromethane (108 µl, ~2 mmol, 10 equiv) was added and the reaction mixture stirred for 96 h at 30–32 °C. The reaction mixture was directly subjected to column chromatography on silica gel (petroleum ether/EtOAc) to afford the product **4** (13 mg, 26% yield) or ***ent*-4 (**12.5 mg, 25% yield). **^{1}H NMR (600 MHz, $CDCl_3$)**: *δ* 7.96 (d, *J* = 7.74 Hz, 2 H), 7.57 (t, *J* = 7.32 Hz, 1 H), 7.47 (d, *J* = 7.6 Hz, 2 H), 4.59 (dd, *J* = 12.6, 4.5 Hz, 1 H), 4.37 (dd, *J* = 12.6, 7.4 Hz, 1 H), 3.23 (dd, *J* = 17.7, 4.1 Hz, 1 H), 3.05 (dd, *J* = 17.6, 7.4 Hz, 1 H), 3.00-2.96 (m, 1 H), 0.99 (s, 9 H); 1**3C{1H} NMR (200 MHz, $CDCl_3$)**: *δ* 198.1, 136.6, 133.3, 128.6 (2 C), 128.0 (2 C), 77.2, 41.9, 37.3, 33.2, 27.4 (3 C); **IR** (ATR): 2963, 1685, 1548, 1375, 1216, 1181, cm^{-1}; **HRMS (ESI)** calcd for $C_{14}H_{19}NO_3$ $[M + H]^+$: 250.1435, found: 250.1434; **HPLC**: The ee was determined by HPLC using a Daicel Chiralcel OD-H [*n*-hexane/*i*-PrOH (97/3)]; flow rate 0.5 mL/min; λ = 220 nm; τ_{major} = 20.92 min, τ_{minor} = 22.77 min; $[\alpha]_D^{23}$ = -16.7 (*c* 2.36, $CHCl_3$, ee = 89.5%).

***ent*-4**: The ee was determined by HPLC using a Daicel Chiralcel OD-H [*n*-hexane/*i*-PrOH (97/3)]; flow rate 0.5 mL/min; λ = 220 nm; τ_{minor} = 21.32 min, τ_{major} = 23.03 min; $[\alpha]_D^{23}$ = +14.6 (*c* 3.1, $CHCl_3$, ee = 95%).

10. HPLC traces

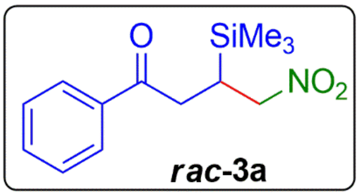

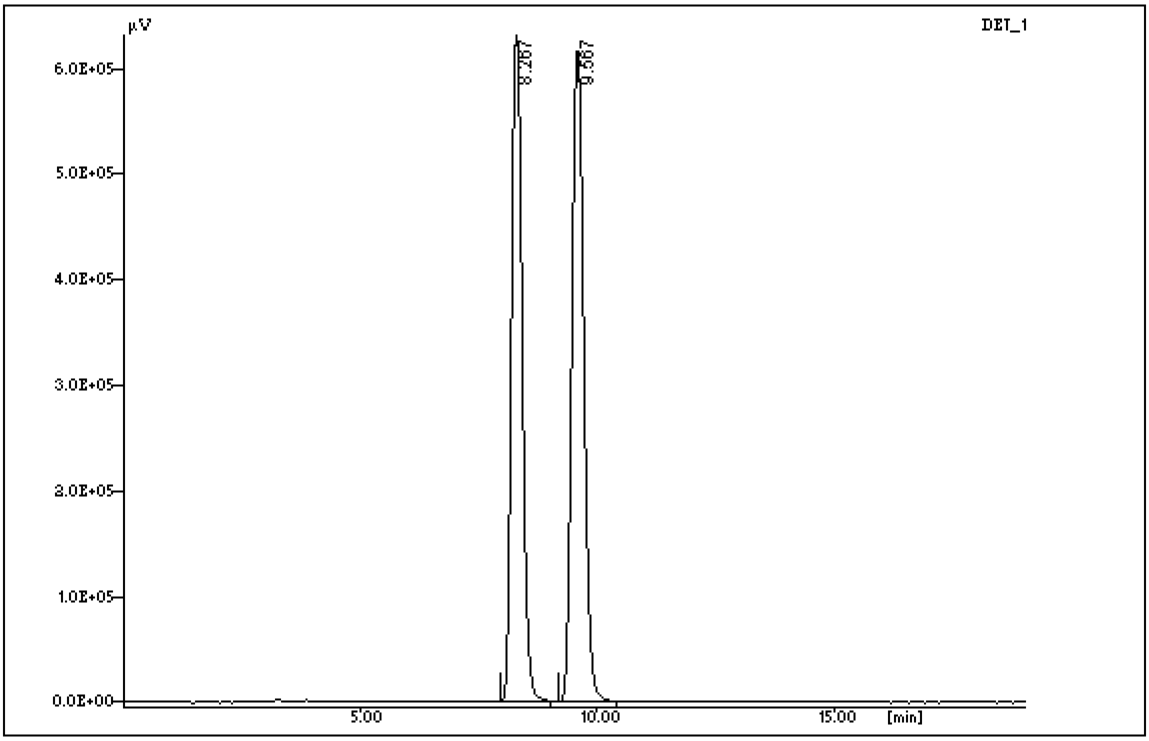

File name : RC-1395-OD-H025.CH1

Control Method : RC220

#	RT	%Area	Area [μV·Sec]	Height[μV]
1	8.26	47.1012	8477351.7297	631522
2	9.56	52.8988	9520803.5000	616163

Total Area of Peak = 17998155.2300 [μV·Sec]

Total Area of Signal = 18128851.0000

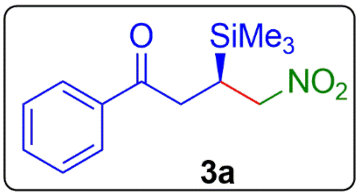

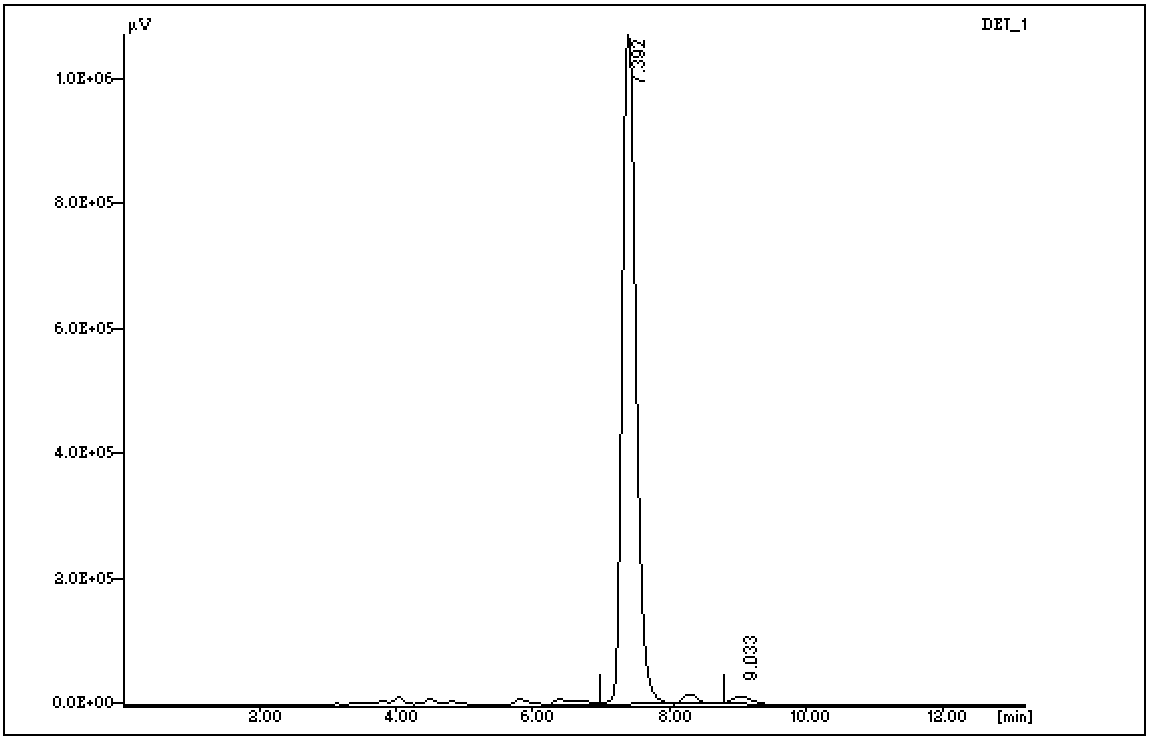

File name : AKD-346-OD-H357.CH1

Control Method :RC220

#	RT	%Area	Area [µV·Sec]	Height[µV]
1	7.39	98.5814	13151631.4120	1069502
2	9.03	1.4186	189252.0000	10337

Total Area of Peak = 13340883.4120 [µV·Sec]

Total Area of Signal = 13496146.5000

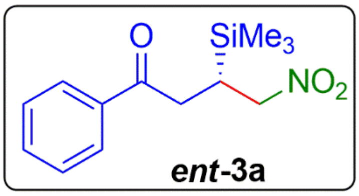

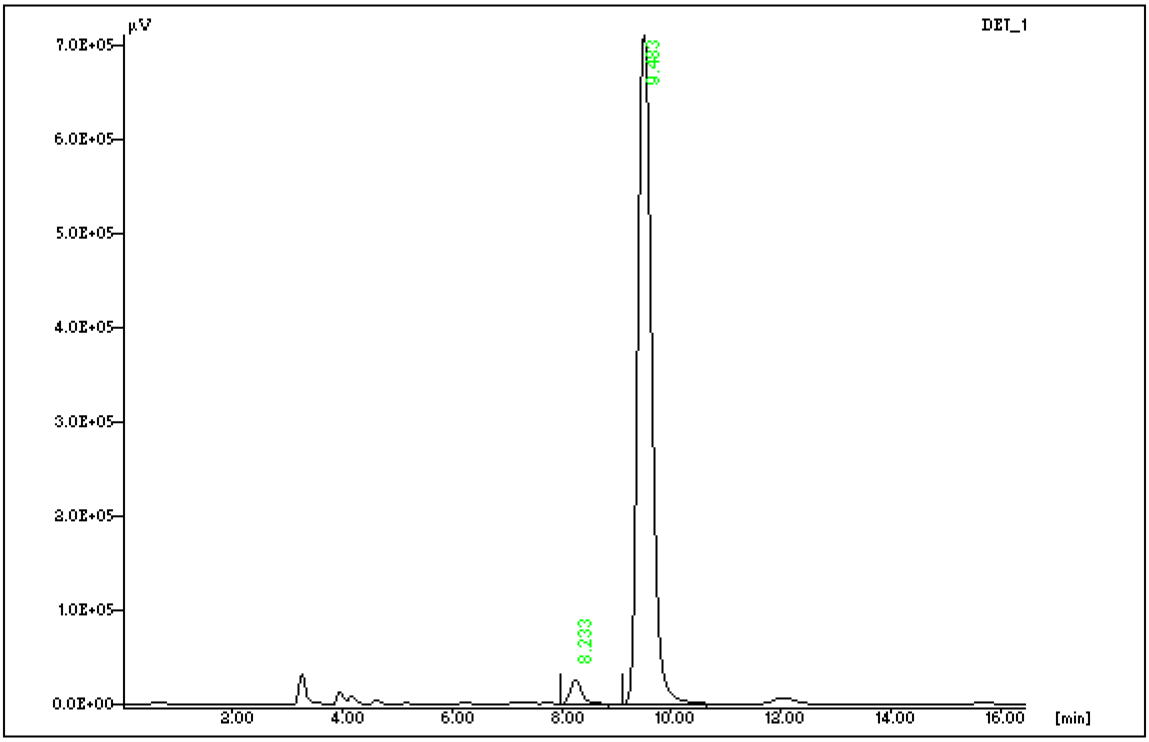

File name : AKD-348-OD-H374.CH1

Control Method :RC220

#	RT	%Area	Area [μV·Sec]	Height[μV]
1	8.23	2.9821	338781.0000	24606
2	9.48	97.0179	11021621.2170	710969

Total Area of Peak = 11360402.2170 [μV·Sec]

Total Area of Signal = 11668120.5000

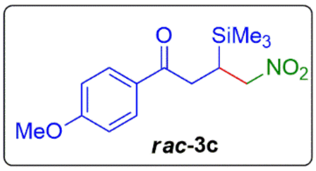

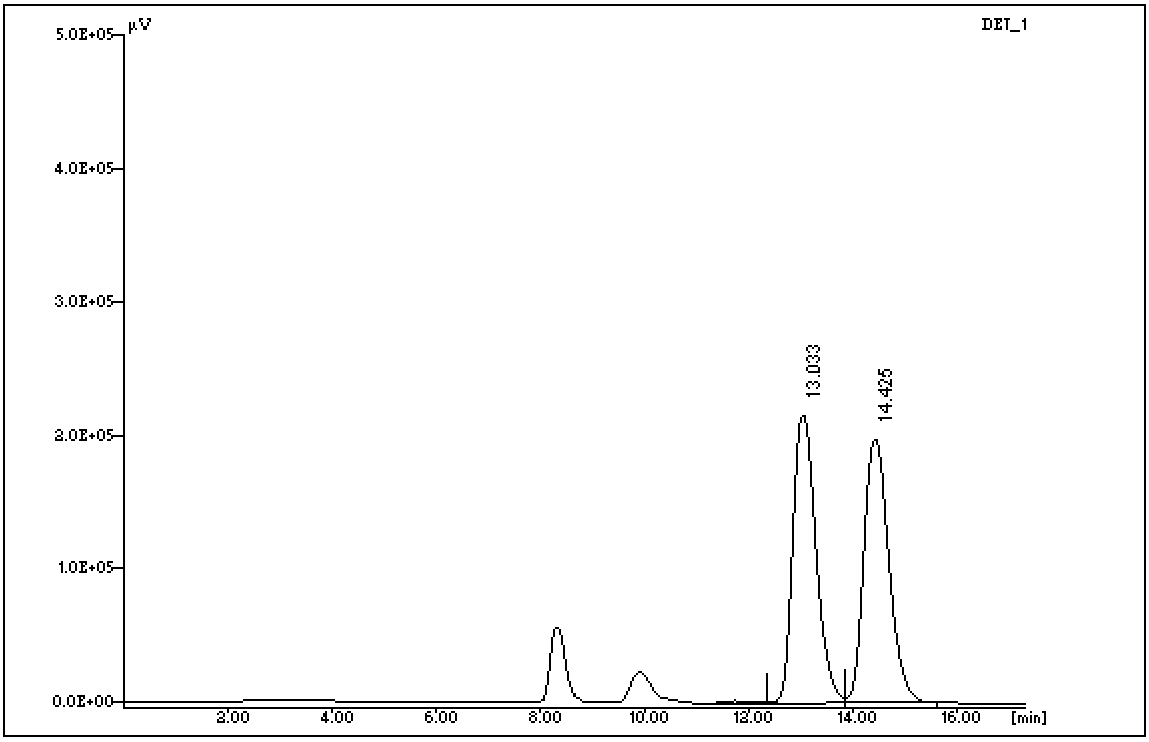

File name : AKD-360-OD-H526.CH1

Control Method :RC220

#	RT	%Area	Area [µV·Sec]	Height[µV]
1	13.03	50.1597	6234070.9514	215874
2	14.45	49.8403	6194380.5778	197267

Total Area of Peak = 12428451.5290 [µV·Sec]

Total Area of Signal = 13299597.0000

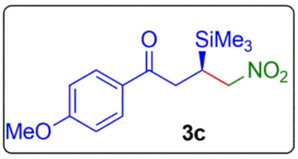

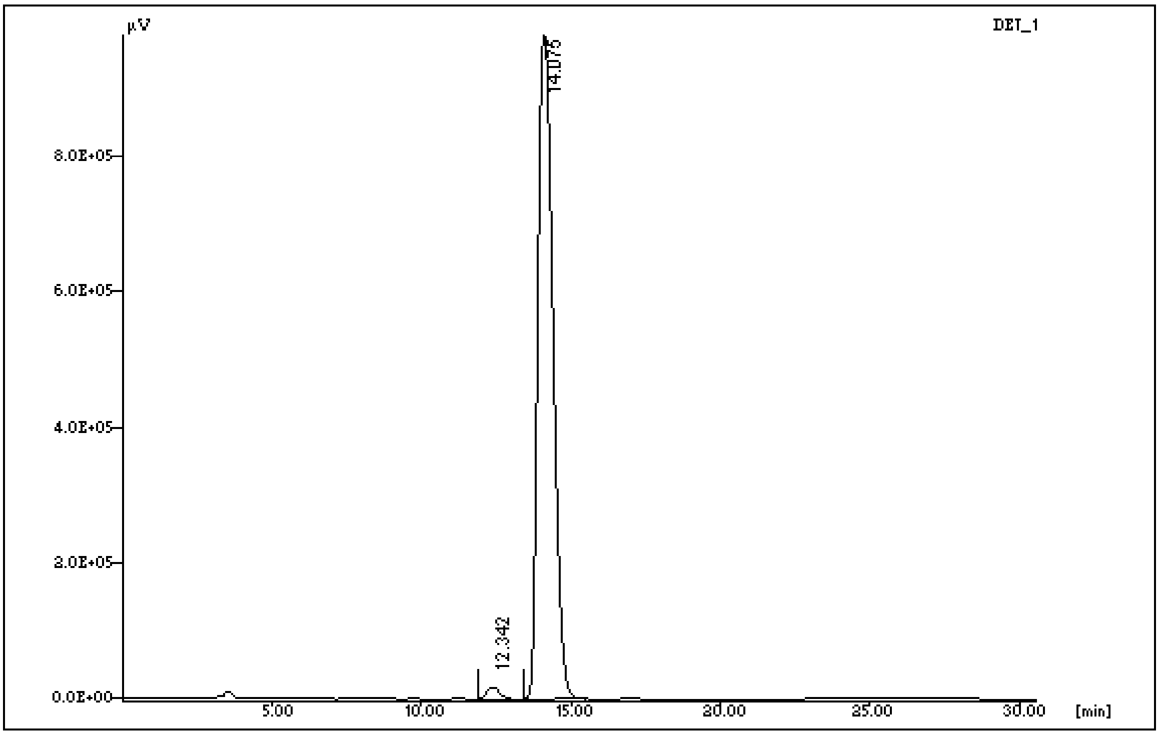

File name : AKD-369R-AD-H116.CH1

Control Method :RC-1-220

#	RT	%Area	Area [µV·Sec]	Height[µV]
1	12.34	1.4157	435037.0000	17136
2	14.07	98.5843	30293575.5000	978586

Total Area of Peak = 30728612.5000 [µV·Sec]

Total Area of Signal = 29863989.5000

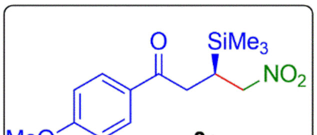

3c

1 mmol scale reaction

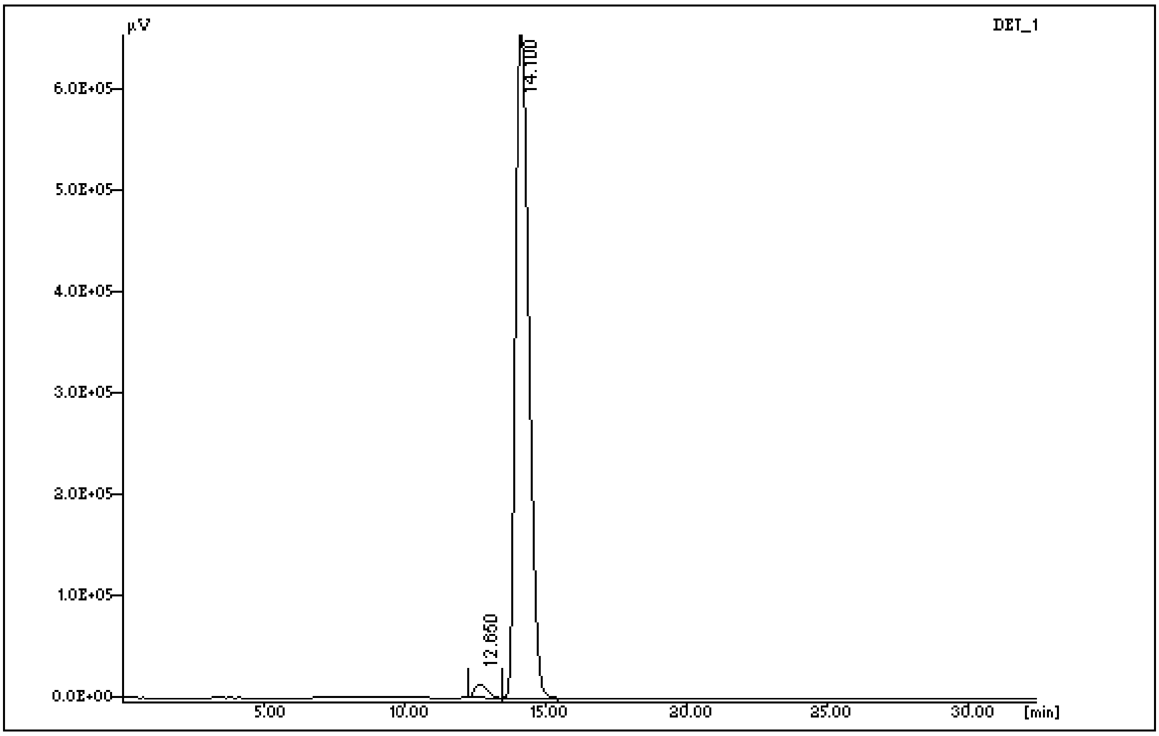

File name : AKD-369R-1 MMOL-AD-H114.CH1

Control Method :RC-1-220

#	RT	%Area	Area [µV·Sec]	Height[µV]
1	12.65	1.7030	338336.1154	13185
2	14.10	98.2970	19528591.5000	653245

Total Area of Peak = 19866927.6150 [µV·Sec]

Total Area of Signal = 18878901.5000

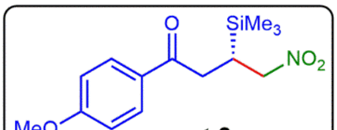

***ent*-3c**

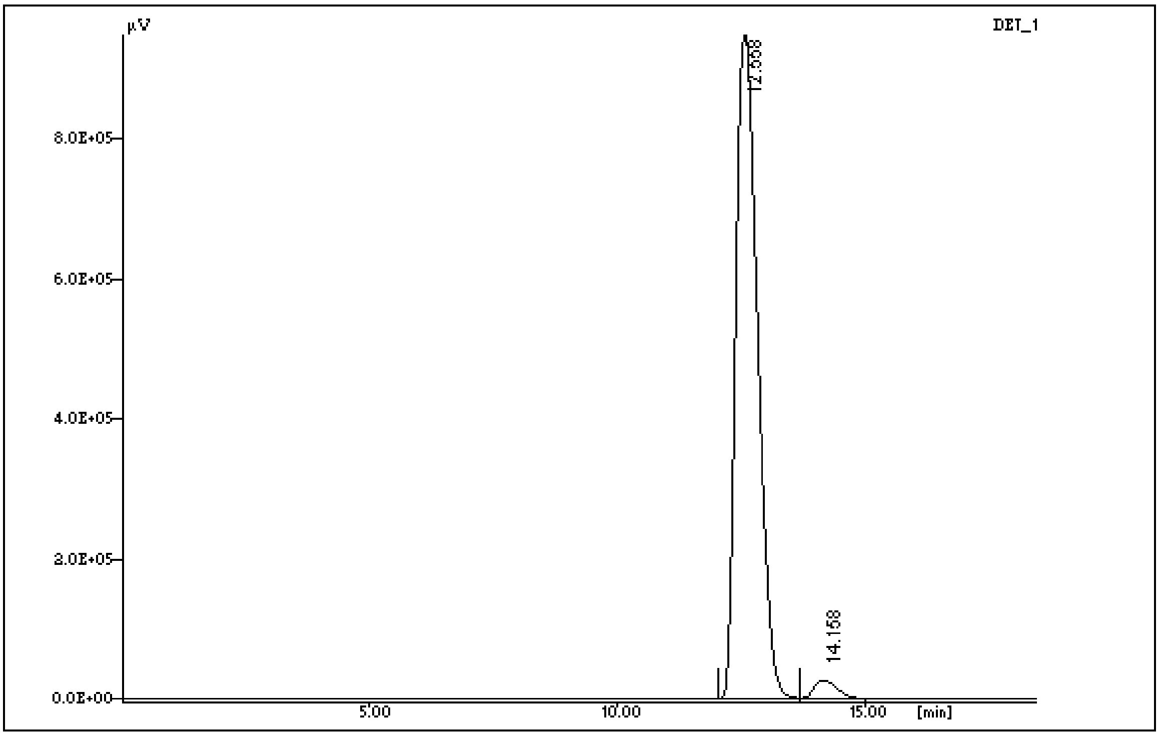

File name : AKD-370R-AD-H123.CH1

Control Method :RC220

#	RT	%Area	Area [µV·Sec]	Height[µV]
1	12.56	97.2050	26807982.0000	946703
2	14.15	2.7950	770827.0000	26134

Total Area of Peak = 27578809.0000 [µV·Sec]

Total Area of Signal = 28193534.0000

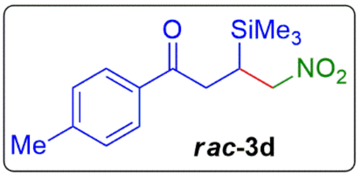

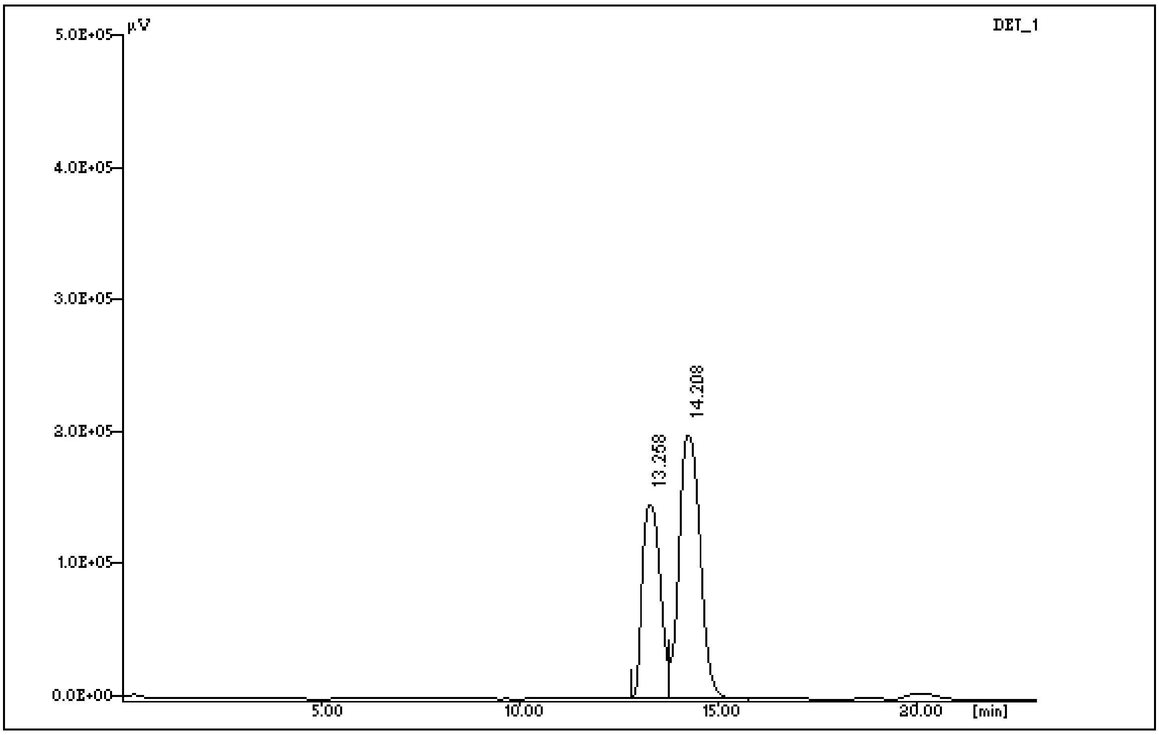

File name : AKD-389-Me-AD-H688.CH1

Control Method :RC-2-220

#	RT	%Area	Area [µV·Sec]	Height[µV]
1	13.28	38.5720	4236388.1532	146448
2	14.28	61.4280	6746672.3468	198858

Total Area of Peak = 10983060.5000 [µV·Sec]

Total Area of Signal = 7838195.5000

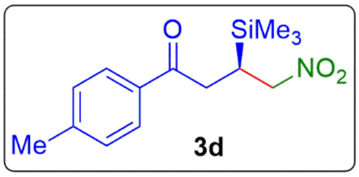

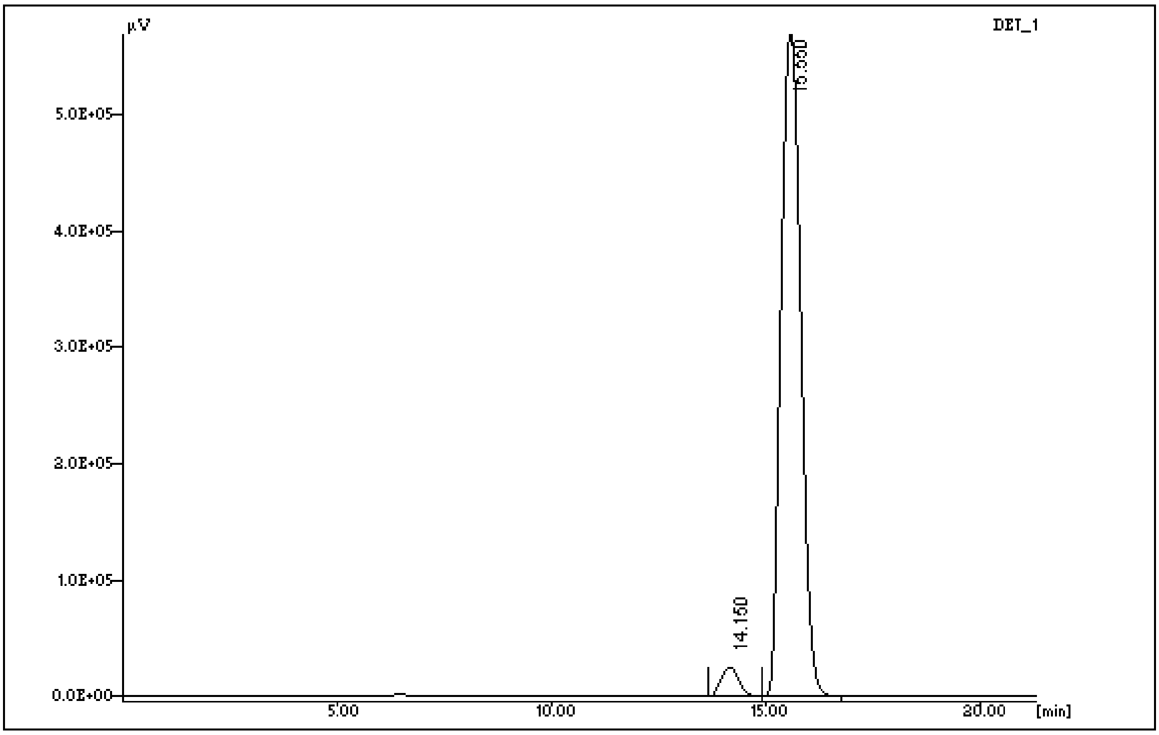

File name : AKD-399-Me-AD-H696.CH1

Control Method :RC-2-220

#	RT	%Area	Area [µV·Sec]	Height[µV]
1	14.10	3.6685	636011.7500	24081
2	15.50	96.3315	16700935.0500	568865

Total Area of Peak = 17336946.8000 [µV·Sec]

Total Area of Signal = 17111083.0000

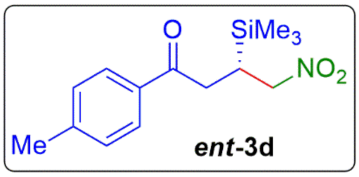

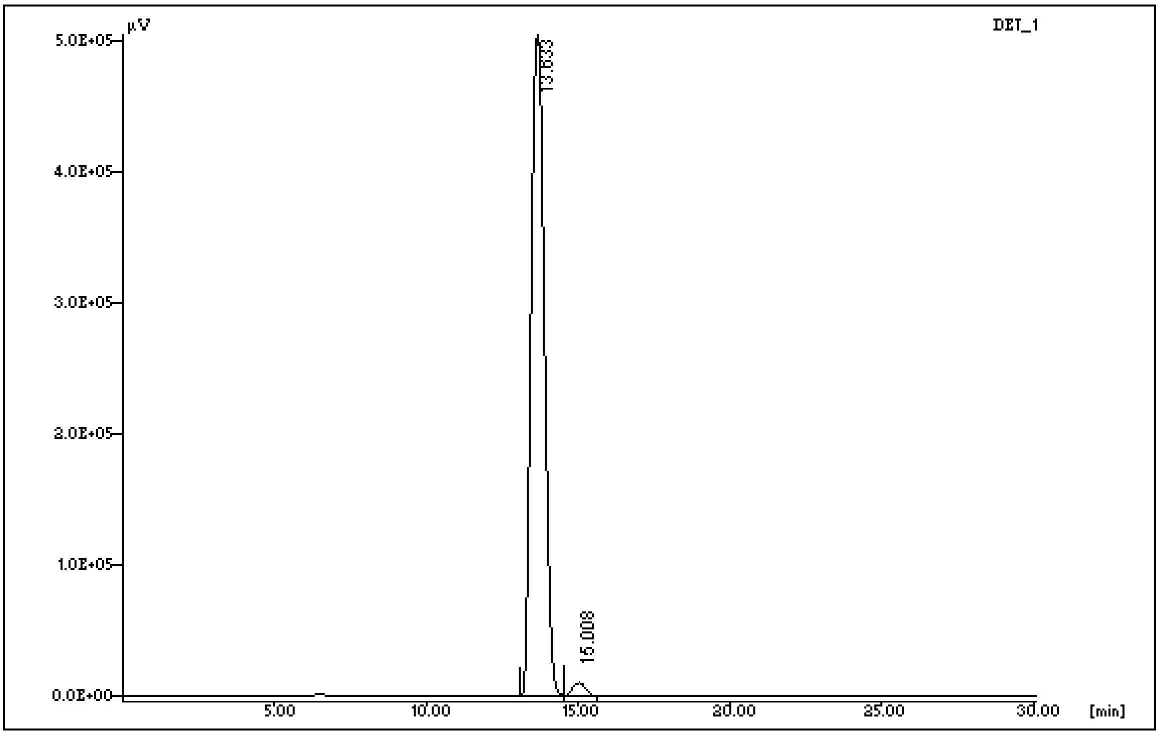

File name : AKD-398-Me-AD-H694.CH1

Control Method :RC-2-220

#	RT	%Area	Area [µV·Sec]	Height[µV]
1	13.63	97.9114	13286956.9710	505307
2	15.08	2.0886	283438.0291	10018

Total Area of Peak = 13570395.0000 [µV·Sec]

Total Area of Signal = 12402820.5000

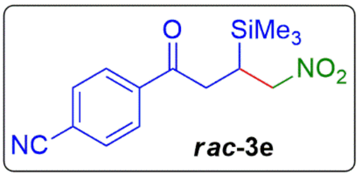

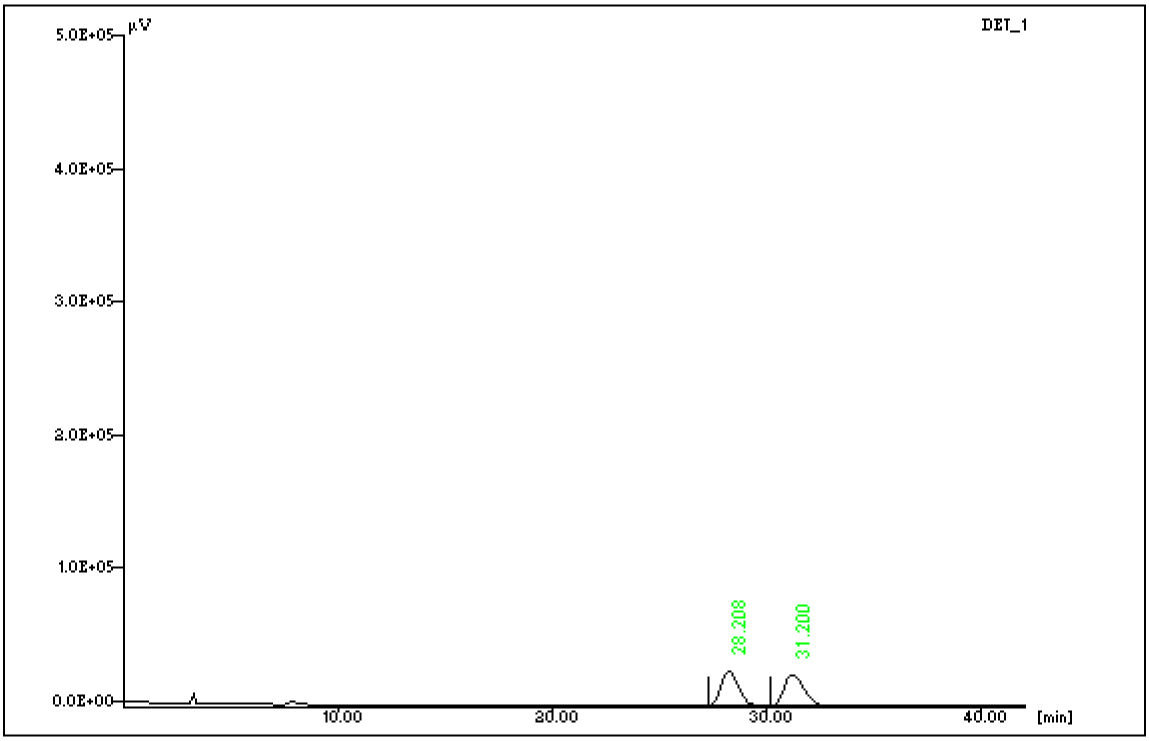

File name : AKD-297-OD-H498.CH1

Control Method :RC220

#	RT	%Area	Area [μV·Sec]	Height[μV]
1	28.28	50.1039	1392802.0000	25500
2	31.20	49.8961	1387023.7500	22540

Total Area of Peak = 2779825.7500 [μV·Sec]

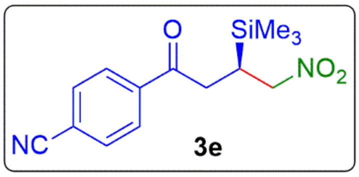

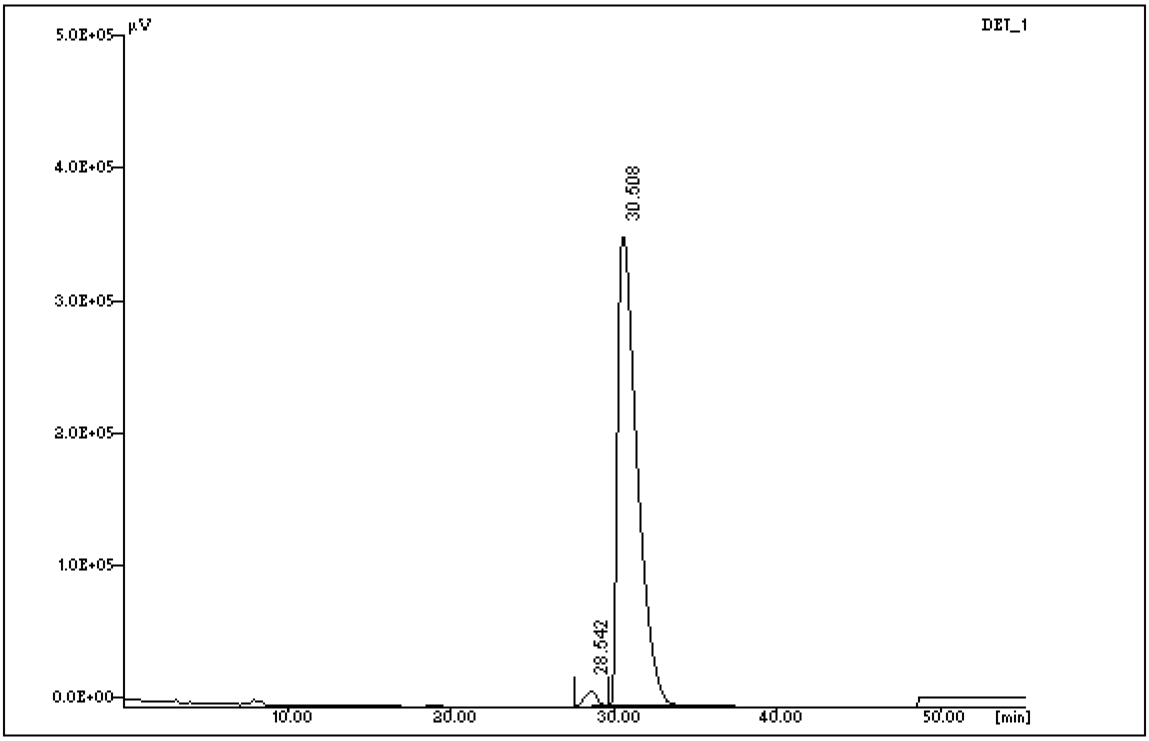

File name : AKD-365-OD-H500.CH1

Control Method :RC220

#	RT	%Area	Area [µV·Sec]	Height[µV]
1	28.52	2.0678	568837.0000	10825
2	30.58	97.9322	26940323.5000	353406

Total Area of Peak = 27509160.5000 [µV·Sec]

Total Area of Signal = 10419634.0000

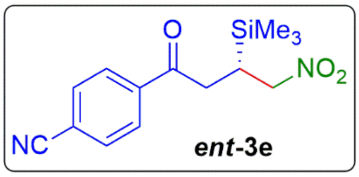

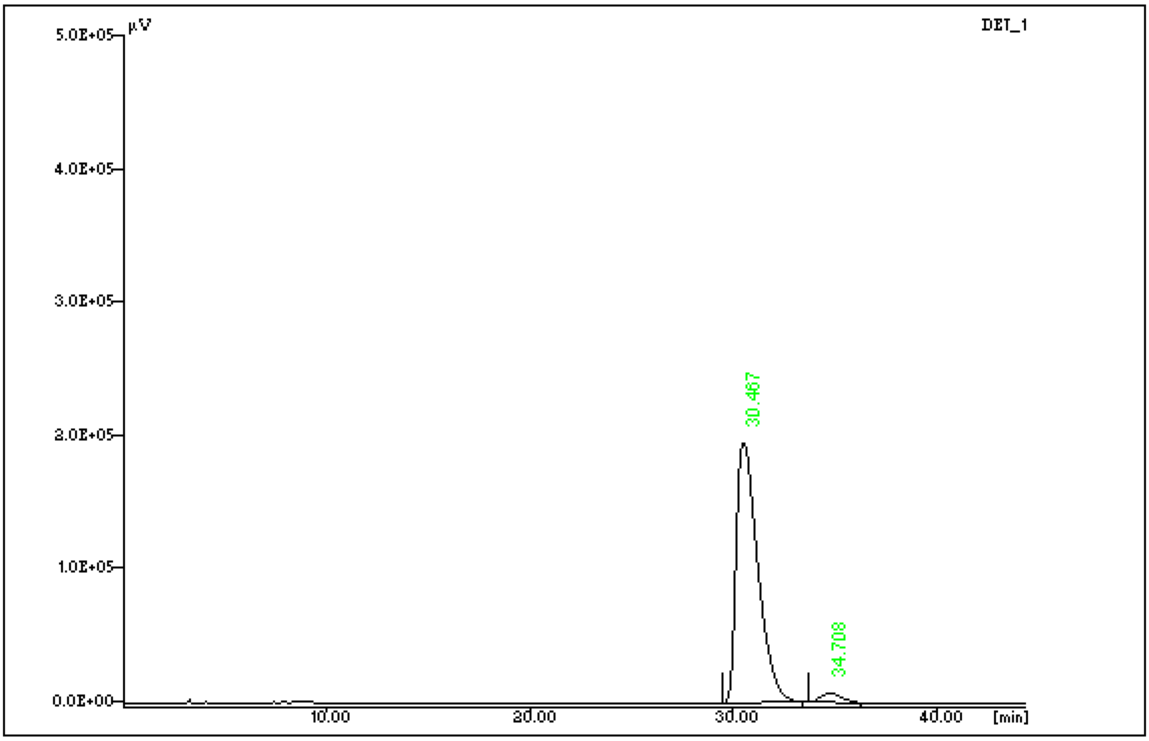

File name : AKD-366-OD-H504.CH1

Control Method :RC220

#	RT	%Area	Area [µV·Sec]	Height[µV]
1	30.47	96.8151	13415905.0000	194938
2	34.78	3.1849	441333.2500	6614

Total Area of Peak = 13857238.2500 [µV·Sec]

Total Area of Signal = 9439679.5000

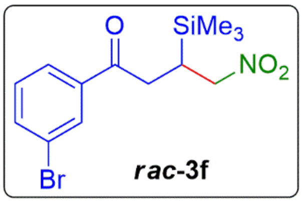

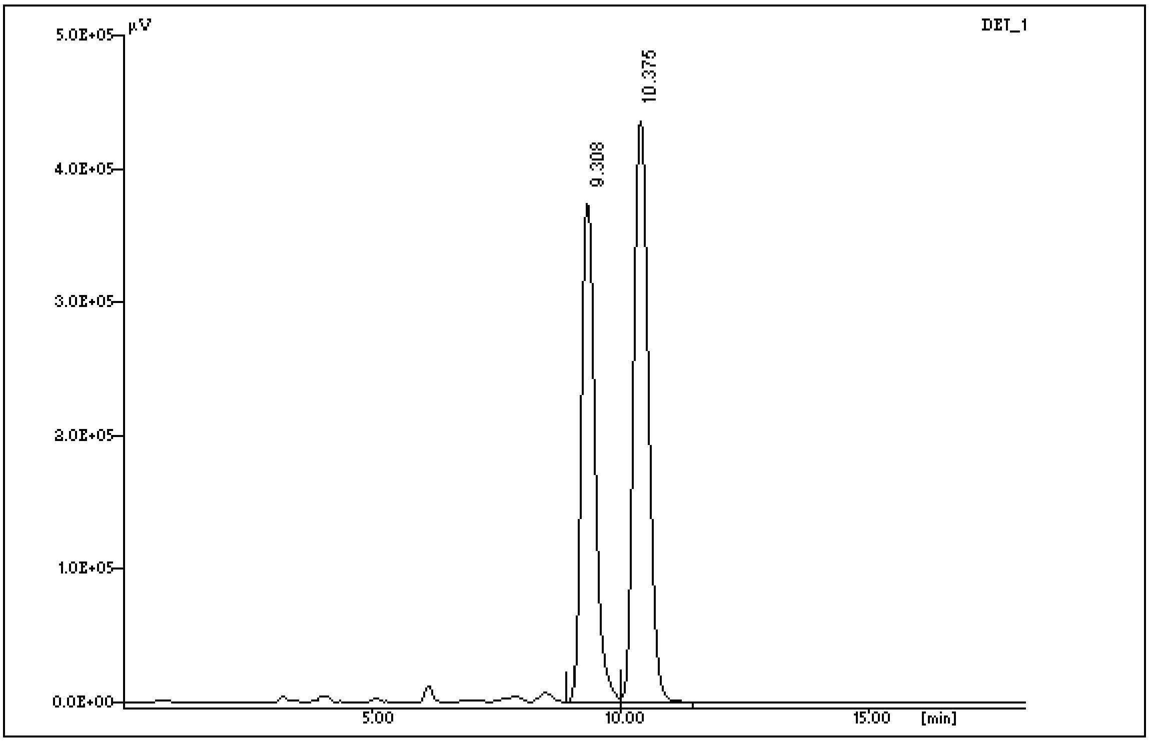

File name : AKD-289-OD-H443.CH1

Control Method :RC220

#	RT	%Area	Area [µV·Sec]	Height[µV]
1	9.30	44.0175	6300532.3148	373615
2	10.35	55.9825	8013171.9180	435675

Total Area of Peak = 14313704.2330 [µV·Sec]

Total Area of Signal = 14482838.5000

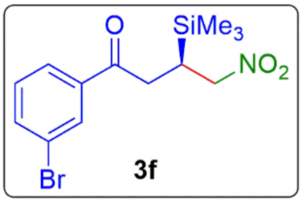

3f

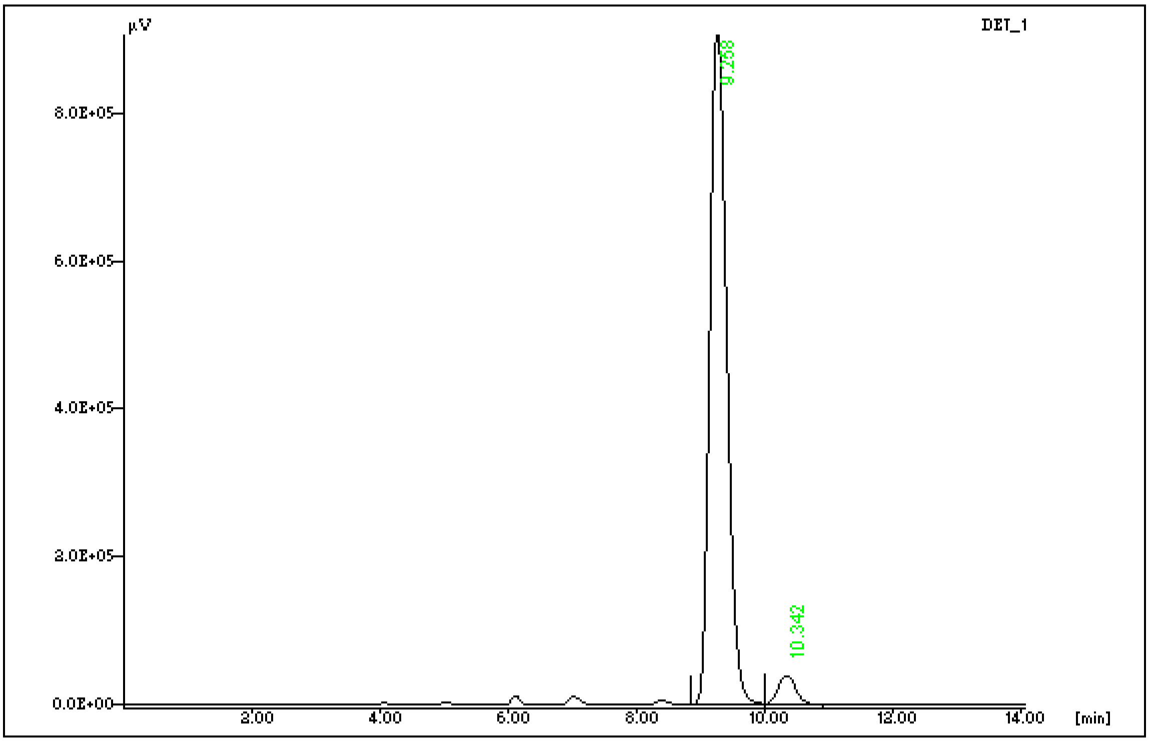

File name : AKD-357-OD-H447.CH1

Control Method :RC220

#	RT	%Area	Area [μV·Sec]	Height[μV]
1	9.25	95.4744	15206911.2950	905366
2	10.32	4.5256	720824.7048	40003

Total Area of Peak = 15927736.0000 [μV·Sec]

Total Area of Signal = 15808909.0000

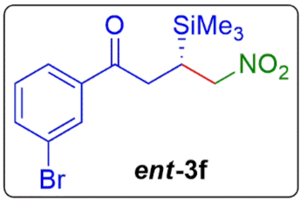

***ent*-3f**

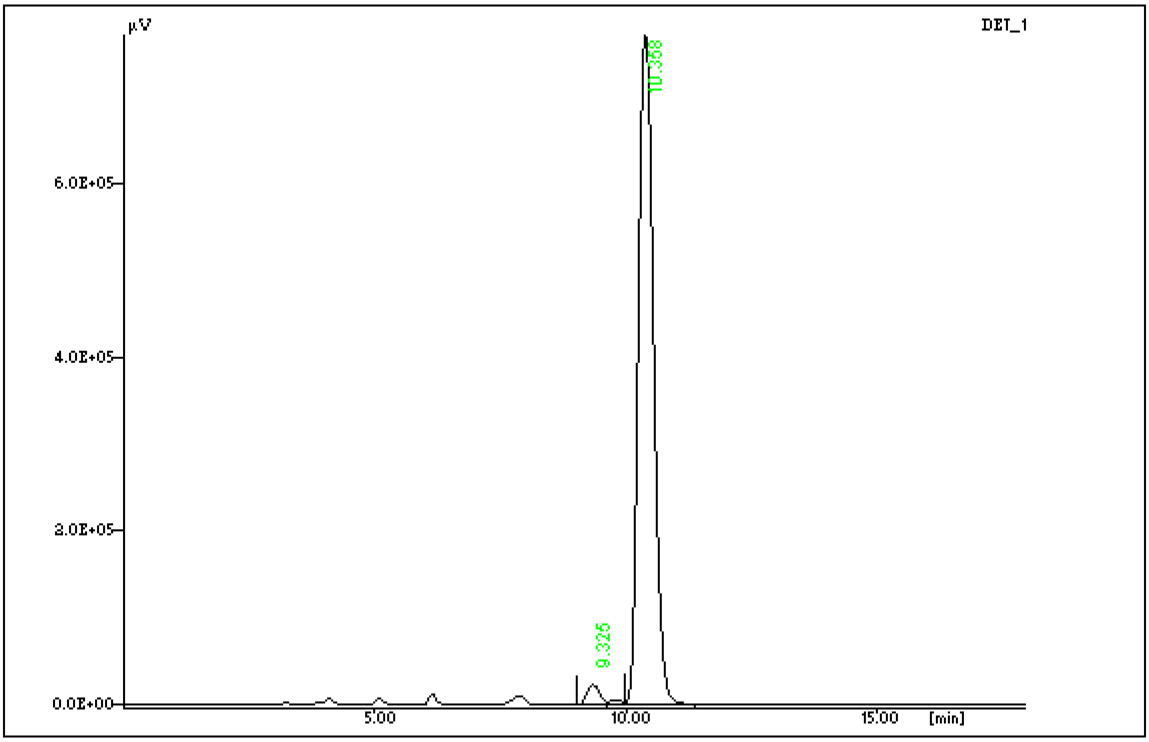

File name : AKD-356-OD-H445.CH1

Control Method :RC220

#	RT	%Area	Area [µV·Sec]	Height[µV]
1	9.32	2.3932	351868.2641	22348
2	10.38	97.6068	14351179.9750	772246

Total Area of Peak = 14703048.2400 [µV·Sec]

Total Area of Signal = 14644125.5000

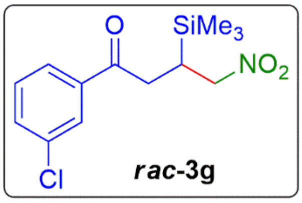

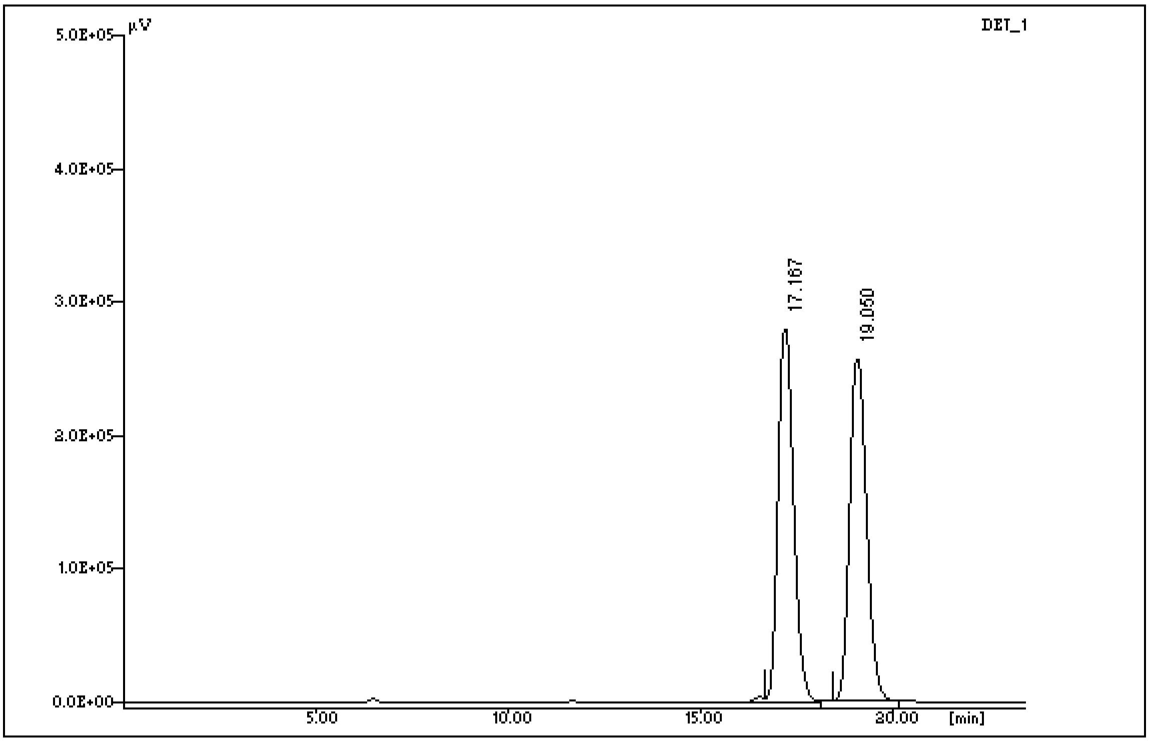

File name : AKD-361-OD-H537.CH1

Control Method :RC-1220

#	RT	%Area	Area [µV·Sec]	Height[µV]
1	17.17	49.7263	7078983.5780	280170
2	19.00	50.2737	7156904.1499	256287

Total Area of Peak = 14235887.7280 [µV·Sec]

Total Area of Signal = 13887309.5000

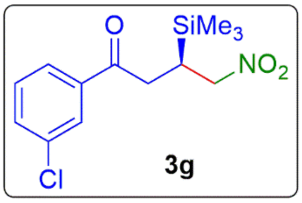

3g

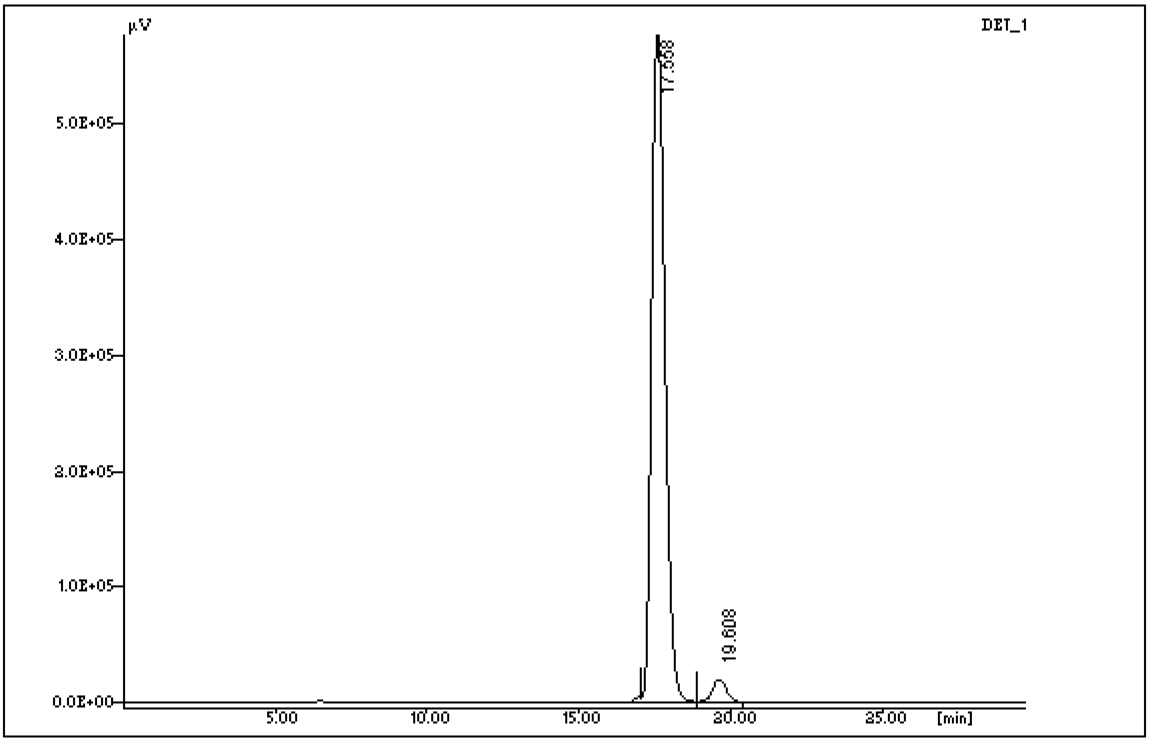

File name : AKD-371-OD-H538.CH1

Control Method :RC-1220

#	RT	%Area	Area [µV·Sec]	Height[µV]
1	17.58	96.2724	15380244.8630	576943
2	19.68	3.7276	595513.9056	19787

Total Area of Peak = 15975758.7680 [µV·Sec]

Total Area of Signal = 15592526.0000

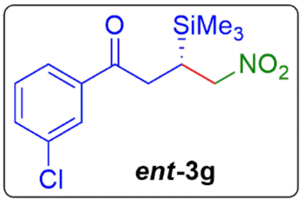

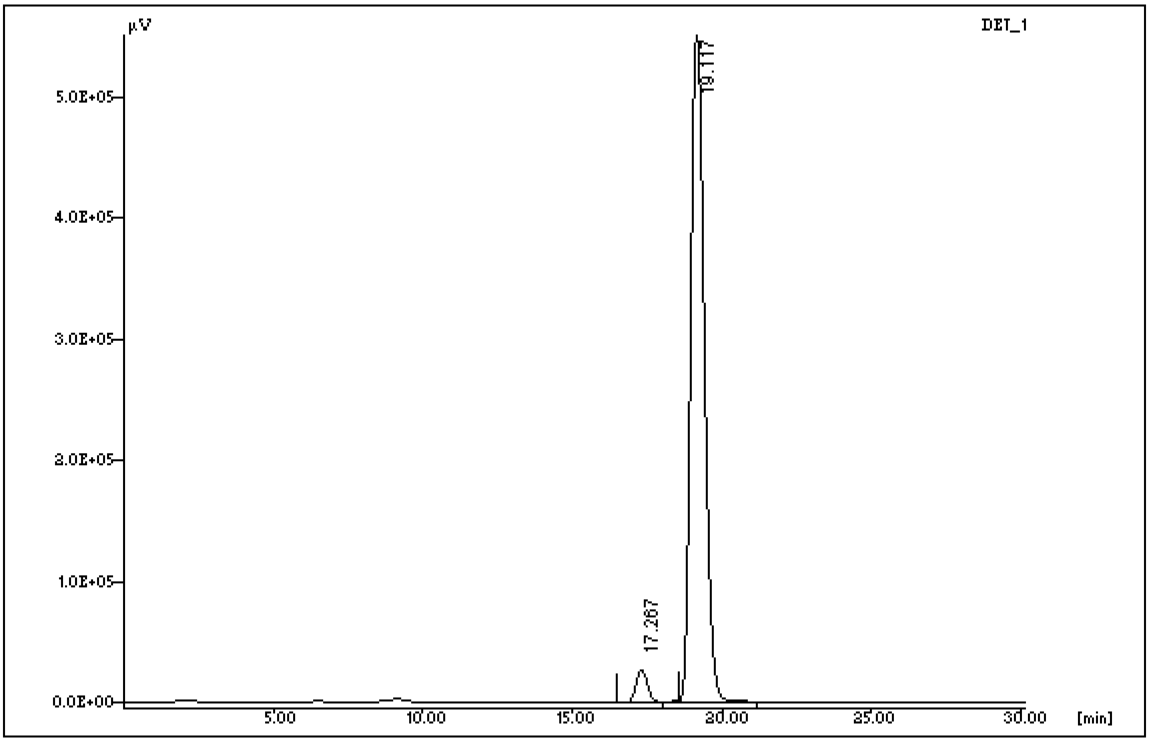

File name : AKD-372-OD-H540.CH1

Control Method :RC-1220

#	RT	%Area	Area [µV·Sec]	Height[µV]
1	17.27	3.9605	630571.7500	26091
2	19.17	96.0395	15290766.9400	550659

Total Area of Peak = 15921338.6900 [µV·Sec]

Total Area of Signal = 16061912.5000

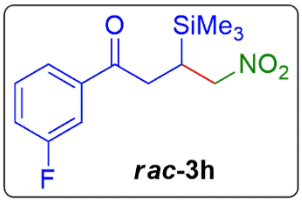

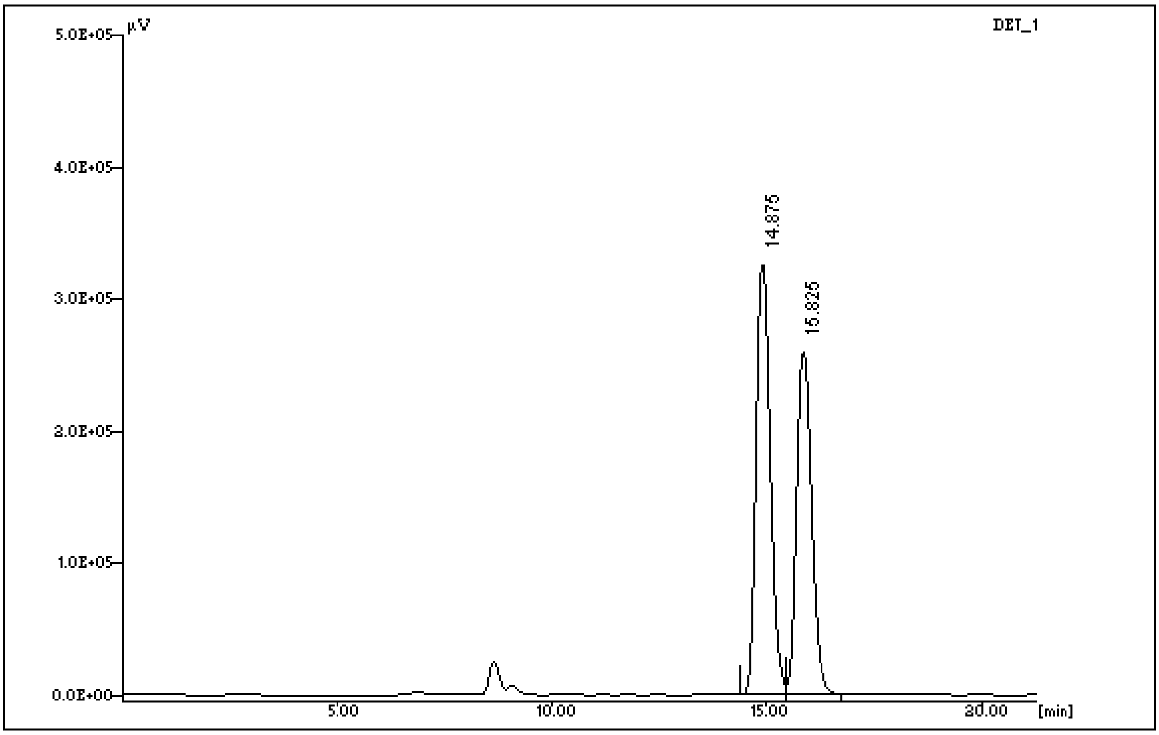

File name : AKD-396-OD-H731.CH1

Control Method :RC-2-220

#	RT	%Area	Area [µV·Sec]	Height[µV]
1	14.85	54.0210	6609458.8010	325651
2	15.83	45.9790	5625511.5247	259072

Total Area of Peak = 12234970.3260 [µV·Sec]

Total Area of Signal = 13250270.5000

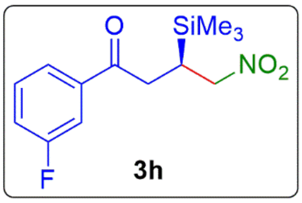

3h

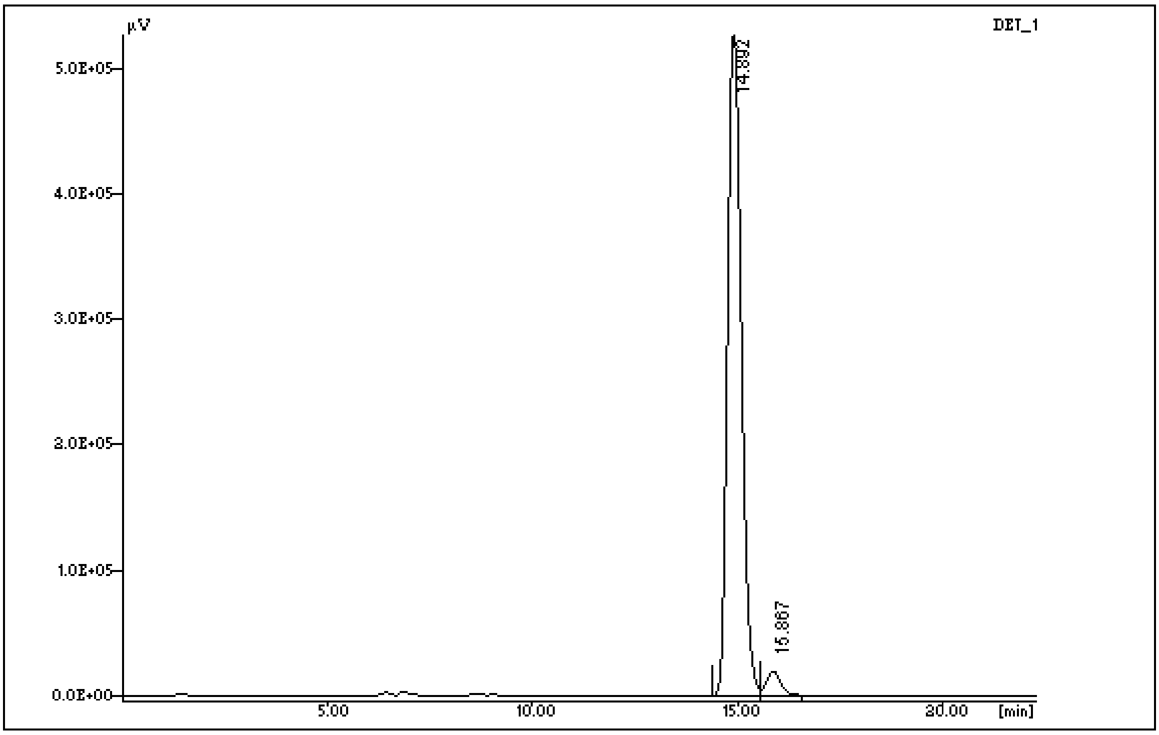

File name : AKD-403-OD-H732.CH1

Control Method :RC-2-220

#	RT	%Area	Area [µV·Sec]	Height[µV]
1	14.89	96.3894	10798620.6350	526751
2	15.87	3.6106	404496.6154	18181

Total Area of Peak = 11203117.2500 [µV·Sec]

Total Area of Signal = 11380713.5000

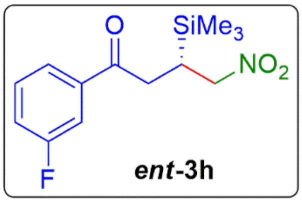

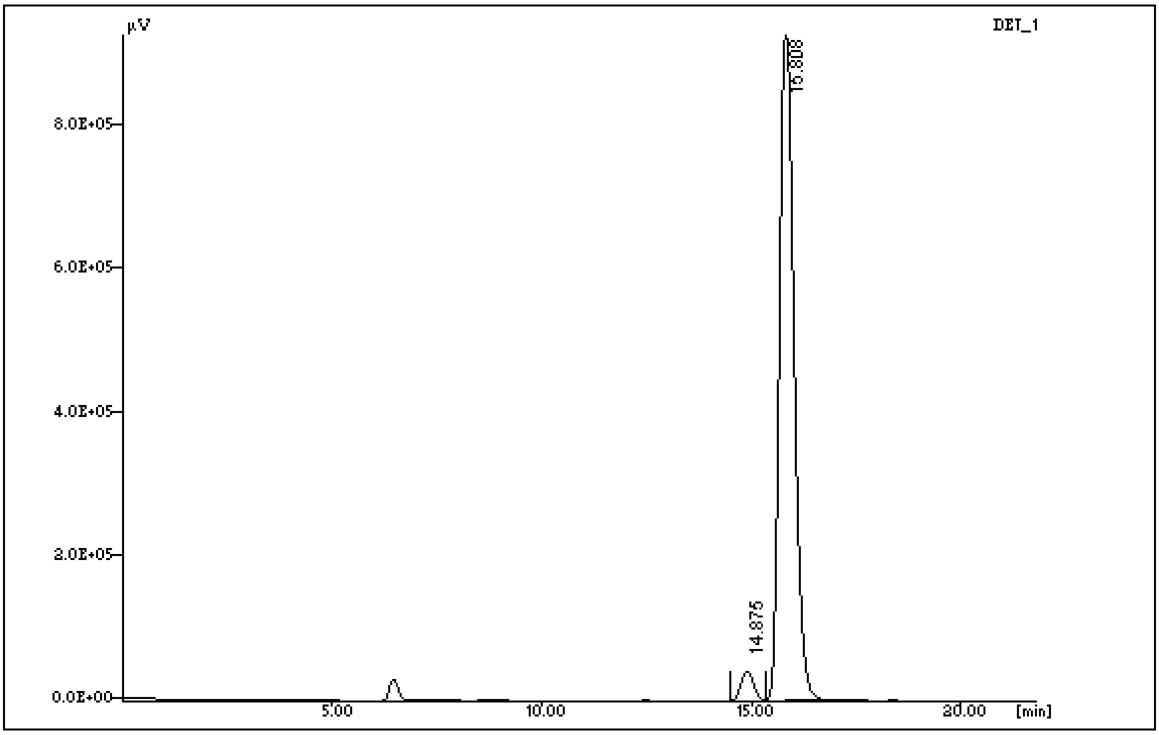

File name : AKD-404-OD-H734.CH1

Control Method :RC-2-220

#	RT	%Area	Area [µV·Sec]	Height[µV]
1	14.88	3.8206	788227.9953	41139
2	15.81	96.1794	19843018.5050	927645

Total Area of Peak = 20631246.5000 [µV·Sec]

Total Area of Signal = 16553402.0000

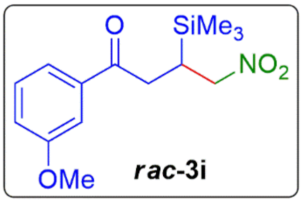

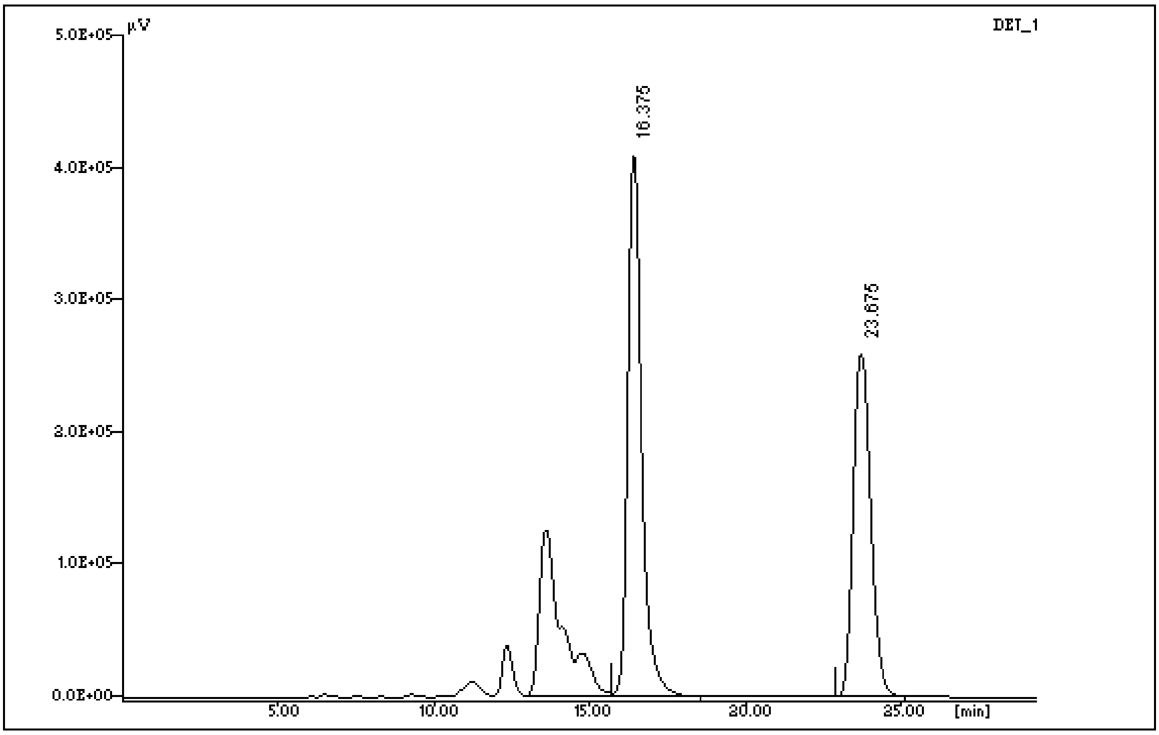

File name : AKD-389-OD-H660.CH1

Control Method :RC-2-220

#	RT	%Area	Area [µV·Sec]	Height[µV]
1	16.38	55.2976	11321145.4340	408254
2	23.68	44.7024	9151968.0000	259390

Total Area of Peak = 20473113.4340 [µV·Sec]

Total Area of Signal = 25856470.0000

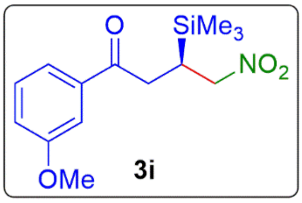

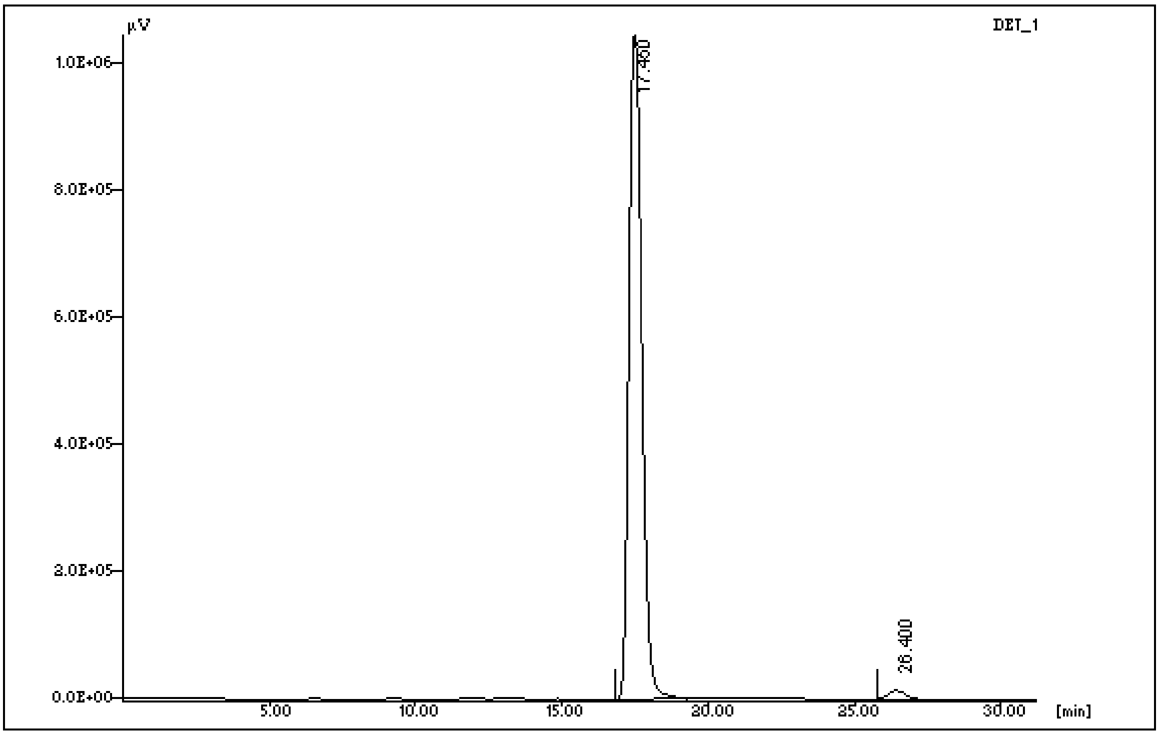

File name : AKD-401-OD-H710.CH1

Control Method :RC-2-220

#	RT	%Area	Area [µV·Sec]	Height[µV]
1	17.45	98.3812	28334706.4330	1042838
2	26.40	1.6188	466230.0000	12742

Total Area of Peak = 28800936.4330 [µV·Sec]

Total Area of Signal = 28161476.0000

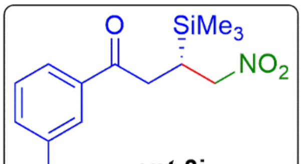

ent-3i

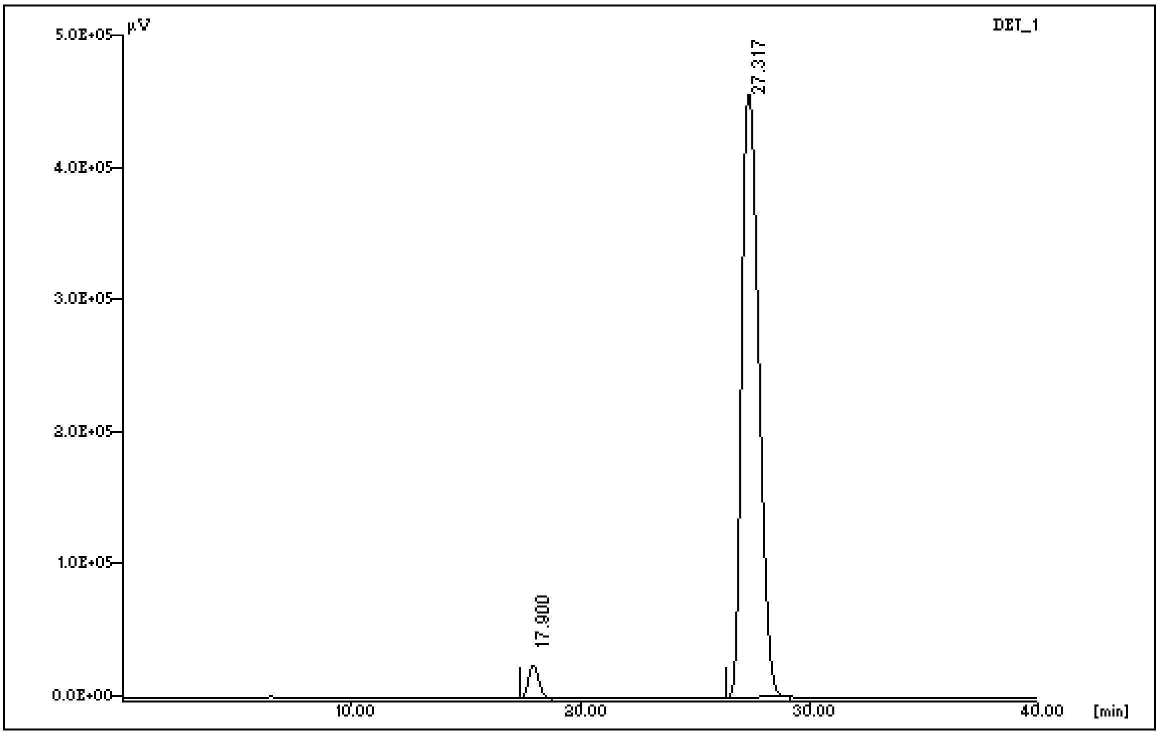

File name : AKD-400-OD-H713.CH1

Control Method :RC-2-220

#	RT	%Area	Area [μV·Sec]	Height[μV]
1	17.90	2.8696	628943.8468	24250
2	27.32	97.1304	21288223.2960	457147

Total Area of Peak = 21917167.1430 [μV·Sec]

Total Area of Signal = 17635563.0000

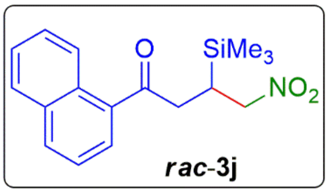

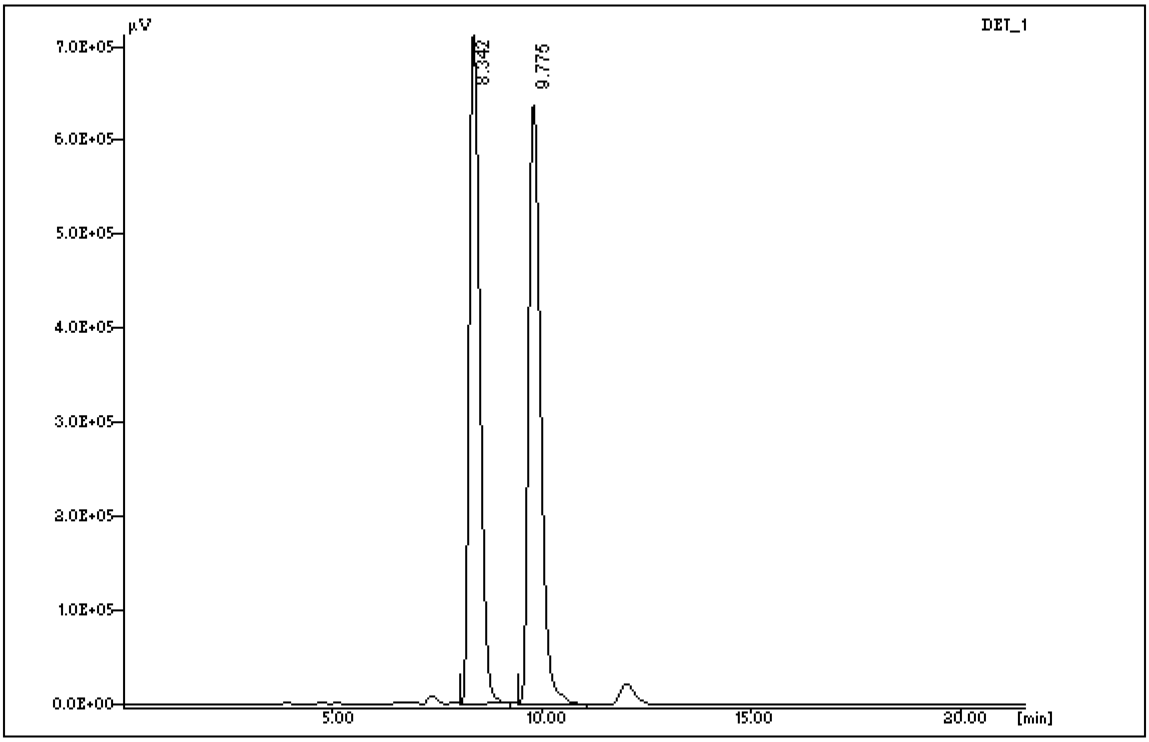

File name : AKD-364-AD-H512.CH1

Control Method :RC220

#	RT	%Area	Area [µV·Sec]	Height[µV]
1	8.34	49.2488	10620215.3330	711735
2	9.78	50.7512	10944214.7500	637533

Total Area of Peak = 21564430.0830 [µV·Sec]

Total Area of Signal = 21645478.0000

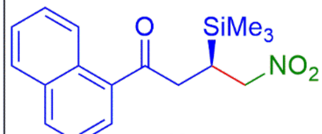

3j

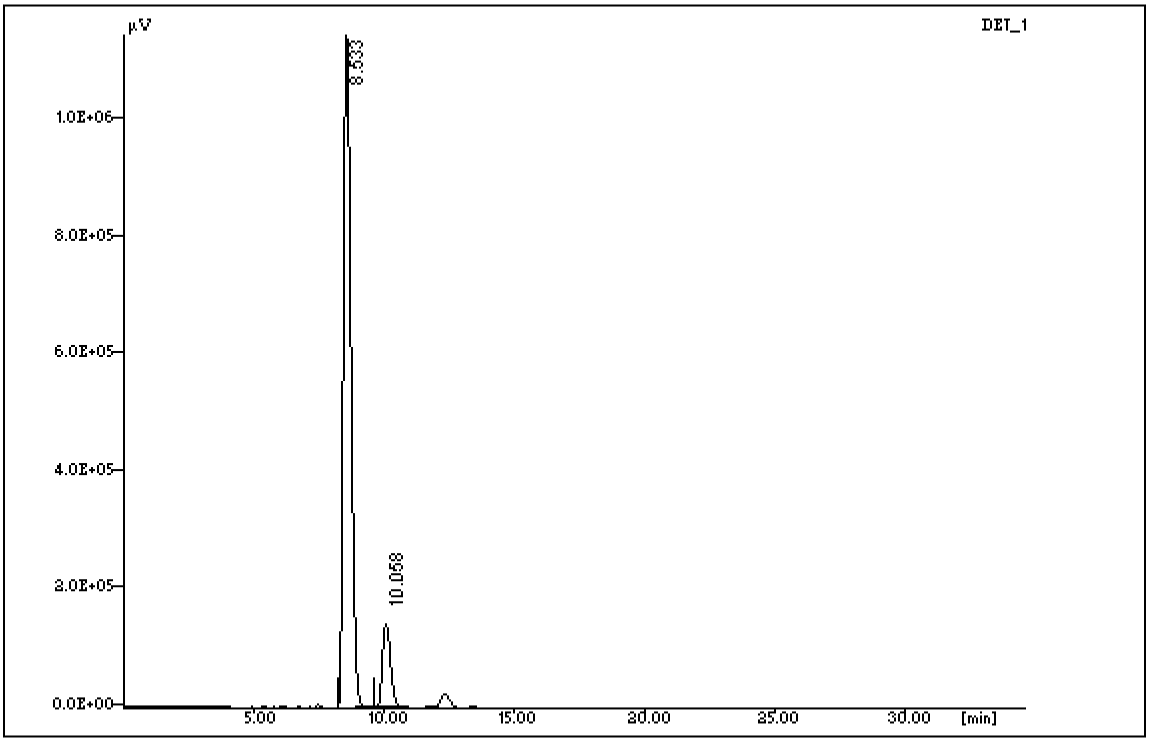

File name : AKD-367-AD-H517.CH1

Control Method :RC220

#	RT	%Area	Area [µV·Sec]	Height[µV]
1	8.53	88.1225	17880464.0000	1146584
2	10.08	11.8775	2409996.2519	140752

Total Area of Peak = 20290460.2520 [µV·Sec]

Total Area of Signal = 8570718.0000

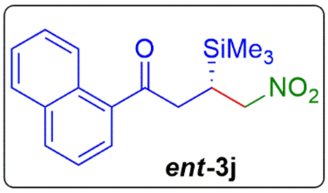

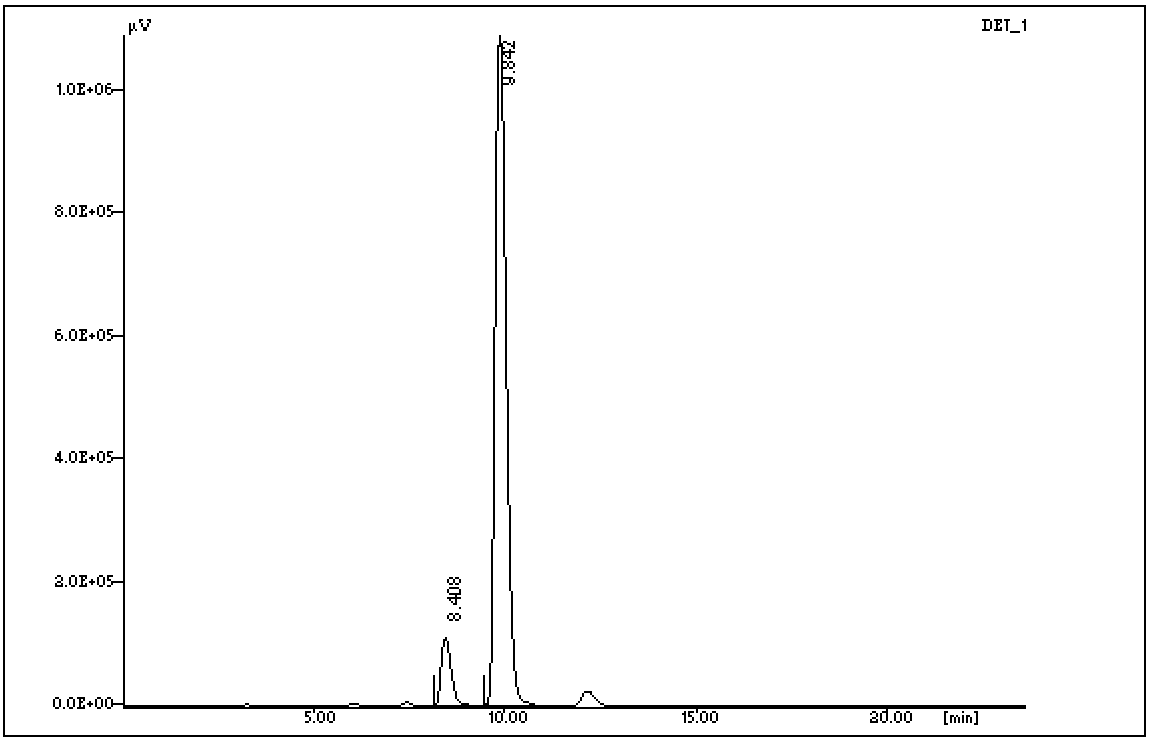

File name : AKD-368-AD-H515.CH1

Control Method :RC220

#	RT	%Area	Area [µV·Sec]	Height[µV]
1	8.40	8.1289	1636453.0000	1146584
2	9.84	91.8711	18494876.0000	1088142

Total Area of Peak = 20131329.0000 [µV·Sec]

Total Area of Signal = 18654089.5000

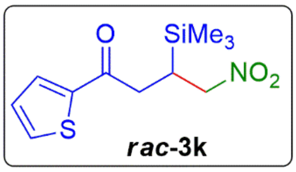

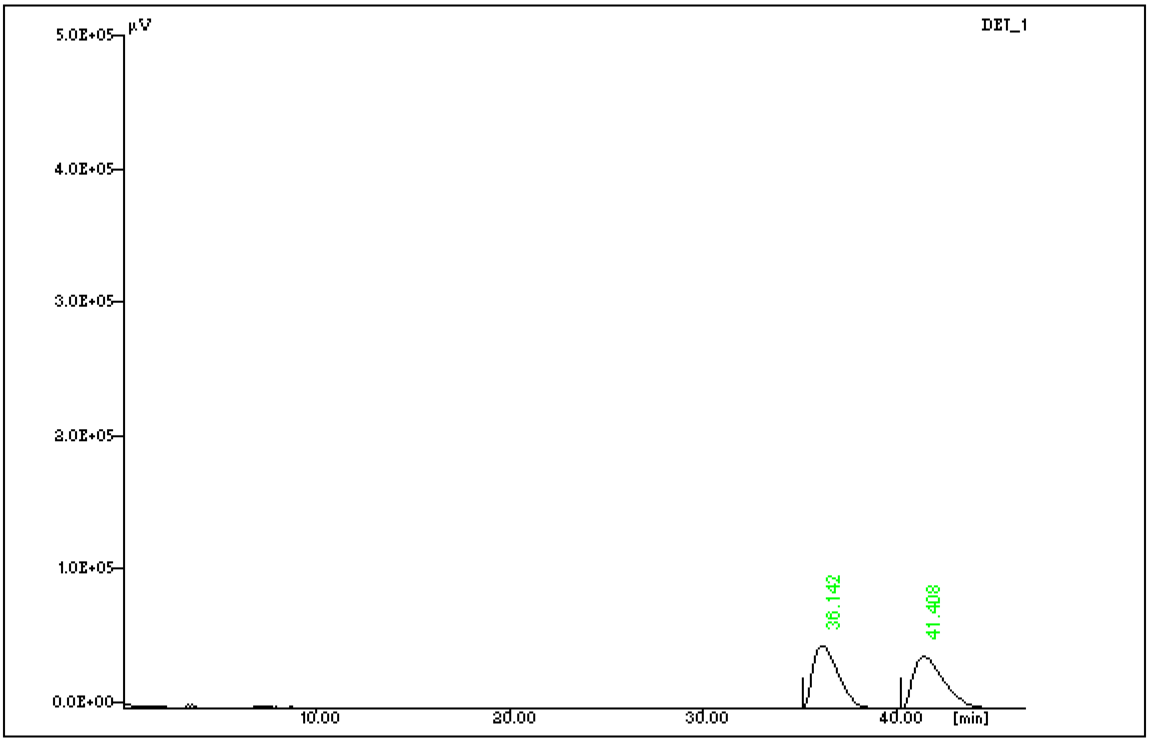

File name : AKD-293-OJ-H457.CH1

Control Method :RC220

#	RT	%Area	Area [μV·Sec]	Height[μV]
1	36.12	50.1969	4032550.2500	46110
2	41.41	49.8031	4000908.5000	38285

Total Area of Peak = 8033458.7500 [μV·Sec]

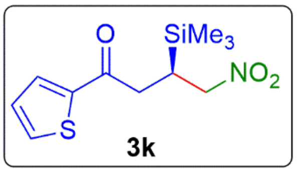

3k

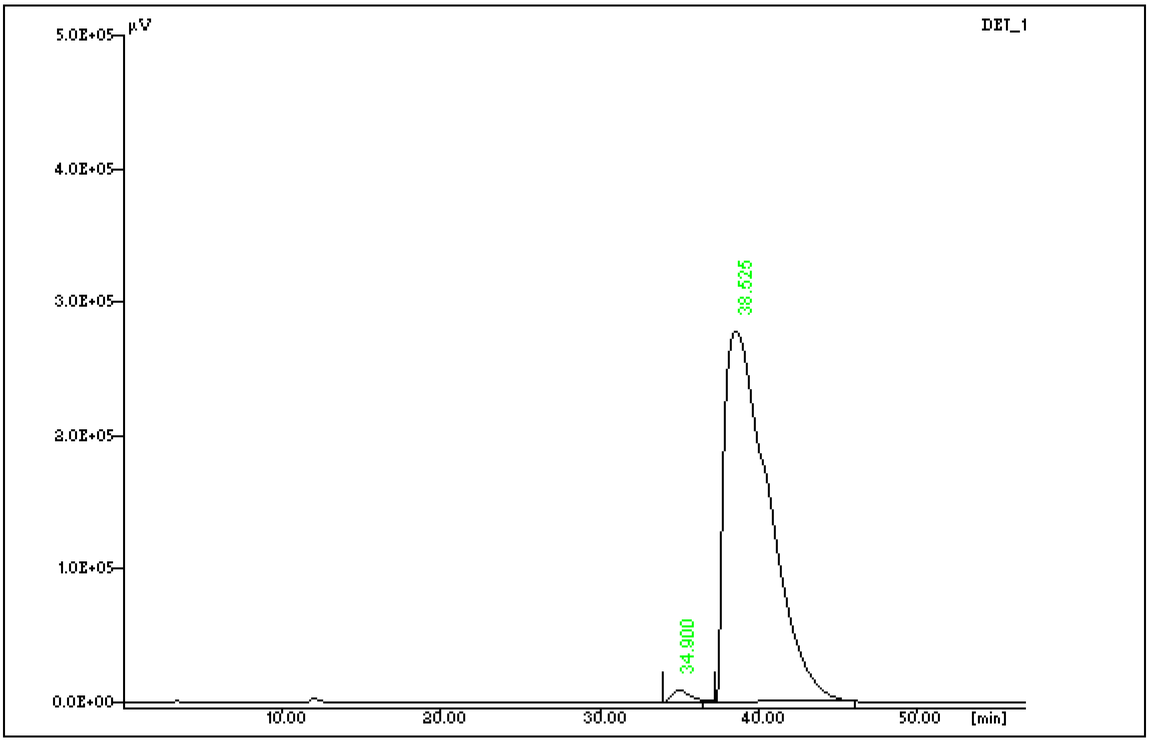

File name : AKD-359-OJ-H469.CH1

Control Method :RC

#	RT	%Area	Area [μV·Sec]	Height[μV]
1	34.90	1.2623	665247.1411	9000
2	38.53	98.7377	52034956.5550	277228

Total Area of Peak = 52700203.6960 [μV·Sec]

Total Area of Signal = 51632324.5000

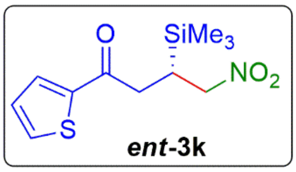

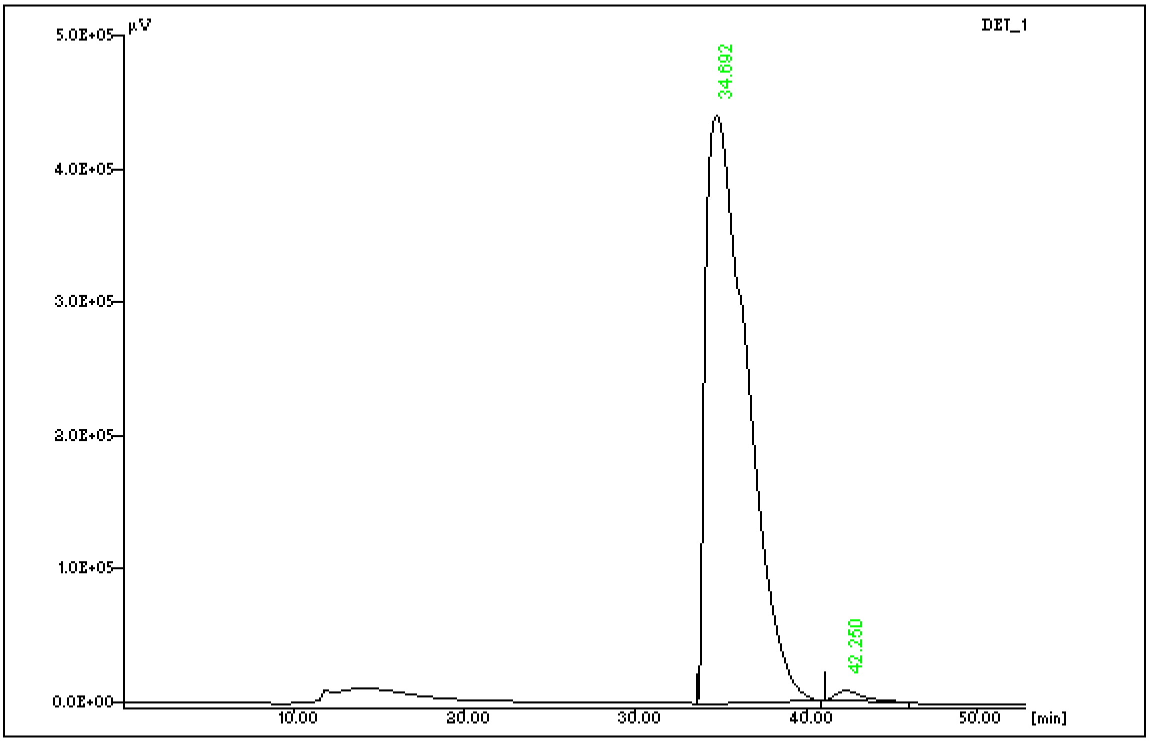

File name : AKD-358-OJ-H467.CH1

Control Method :RC

#	RT	%Area	Area [µV·Sec]	Height[µV]
1	34.69	98.7651	71494687.2500	440262
2	42.20	1.2349	893908.5000	7685

Total Area of Peak = 72388595.7500 [µV·Sec]

Total Area of Signal = 73854160.5000

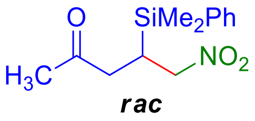

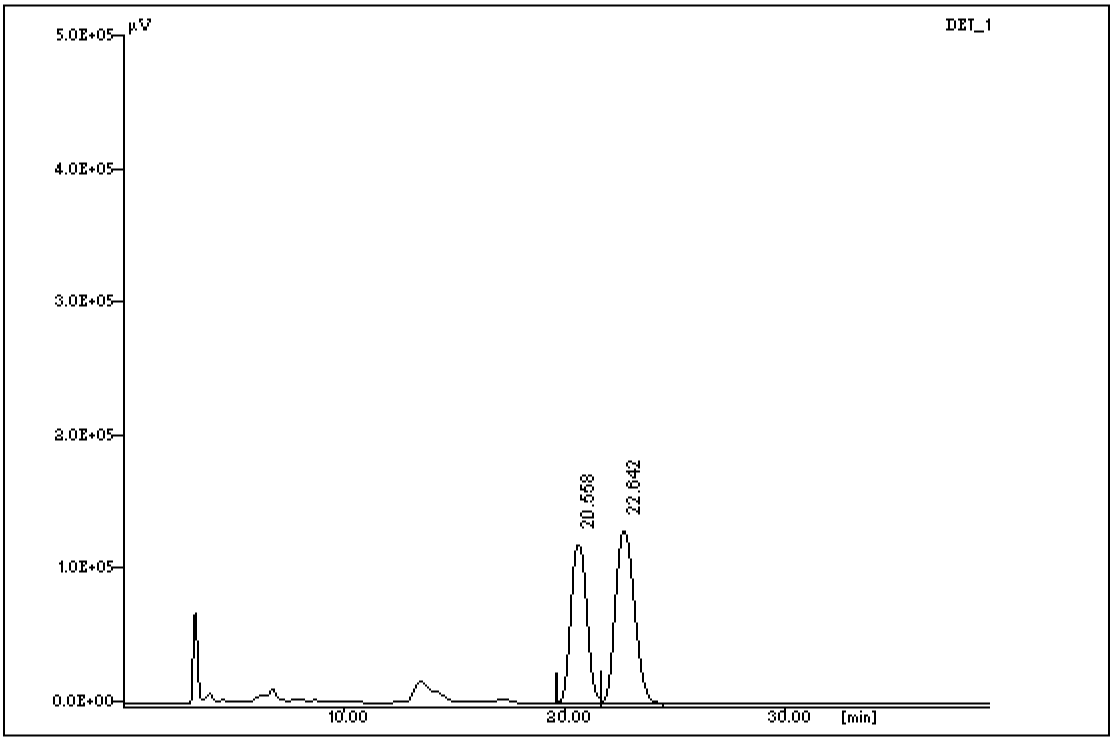

File name : RC-1377-79-OJ-H968.CH1

Control Method :RC220

#	RT	%Area	Area [µV·Sec]	Height[µV]
1	20.56	43.0309	5322327.6974	118049
2	22.64	56.9691	7046288.3026	128311

Total Area of Peak = 12368616.0000 [µV·Sec]

Total Area of Signal = 10945066.0000

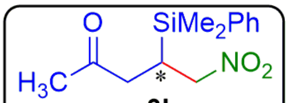

3l

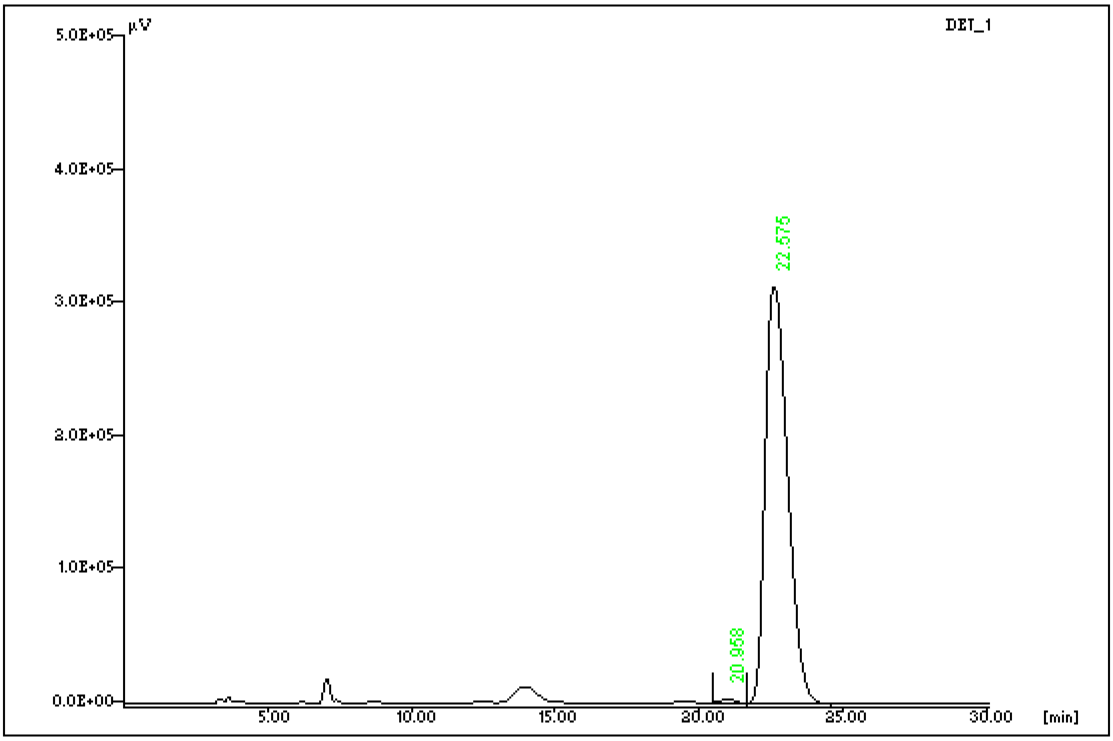

File name : AKD-311-OJ-H139.CH1

Control Method : RC220

#	RT	%Area	Area [μV·Sec]	Height[μV]
1	20.98	0.5283	83657.8838	2485
2	22.55	99.4717	15752933.5000	313142

Total Area of Peak = 15836591.3840 [μV·Sec]

Total Area of Signal = 13556867.500

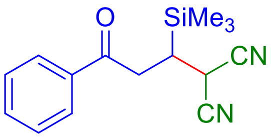

rac

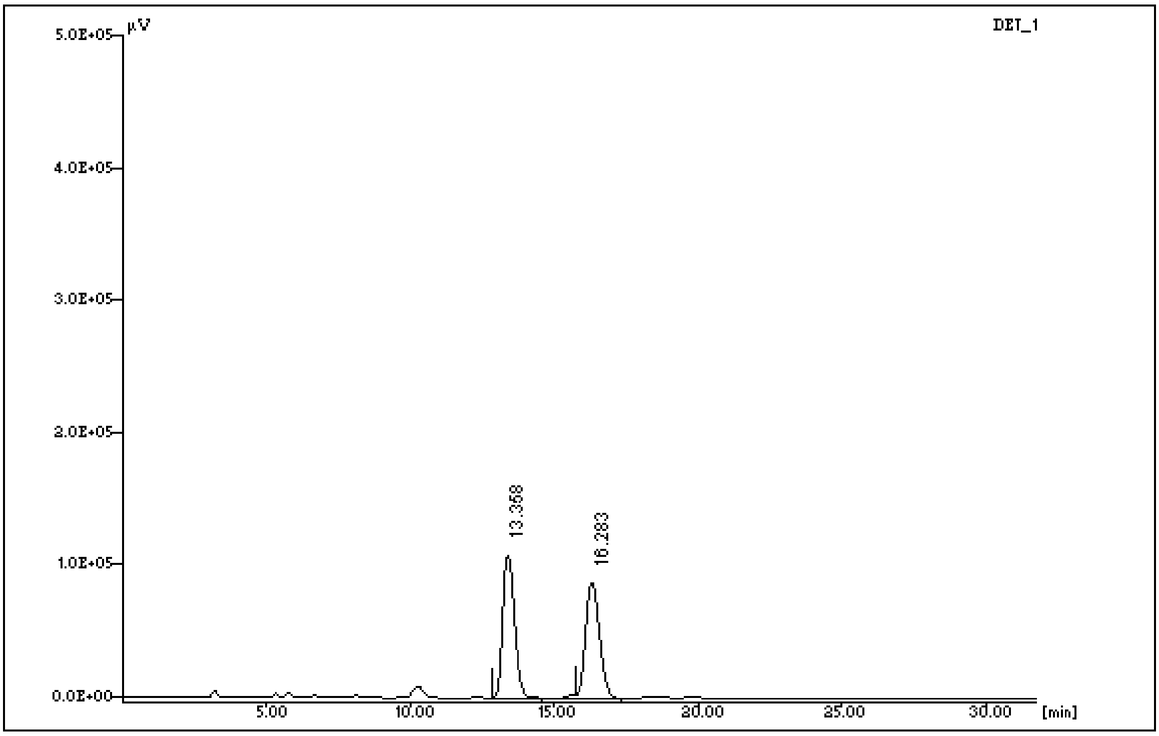

File name : AKD-302-OD-H546.CH1

Control Method :RC220

#	RT	%Area	Area [µV·Sec]	Height[µV]
1	13.36	50.7307	2666193.7500	107600
2	16.28	49.2693	2589389.9880	86144

Total Area of Peak = 5255583.7380 [µV·Sec]

Total Area of Signal = 2923767.0000

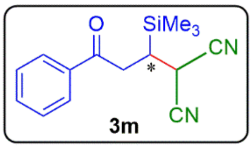

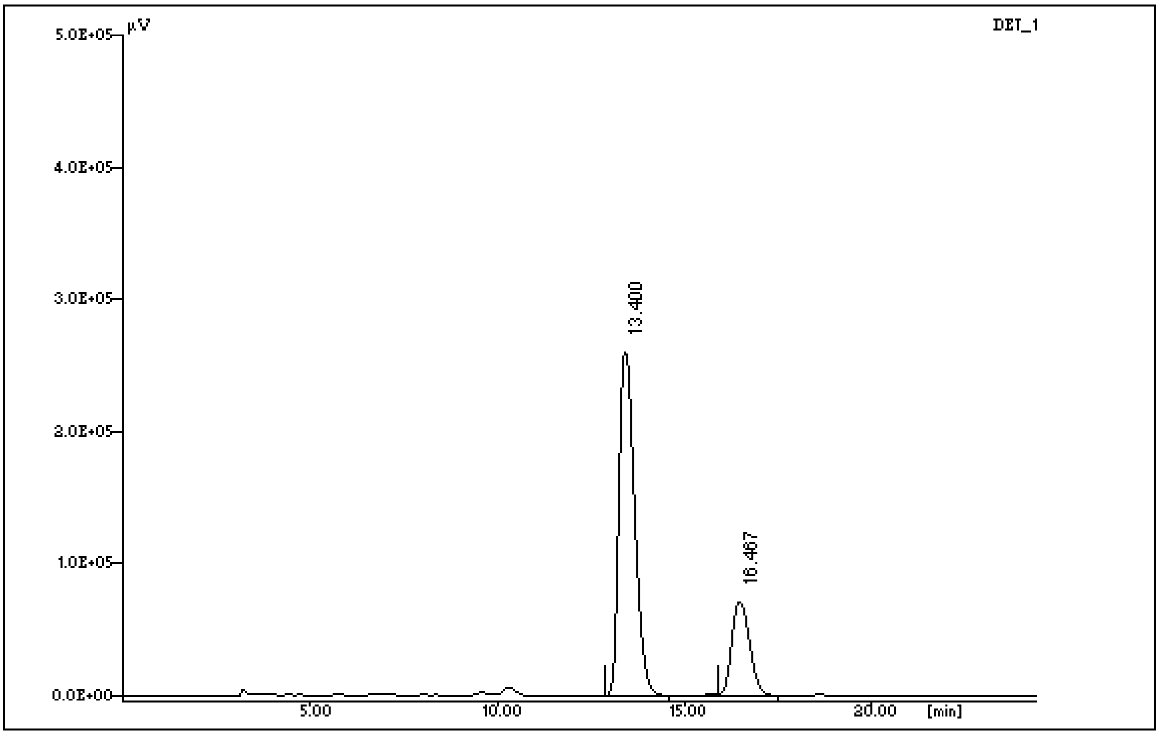

File name : AKD-373-OD-H545.CH1

Control Method :RC220

#	RT	%Area	Area [μV·Sec]	Height[μV]
1	13.40	75.8980	6724426.2500	259660
2	16.47	24.1020	2135395.1566	70143

Total Area of Peak = 8859821.4066 [μV·Sec]

Total Area of Signal = 8744404.5000

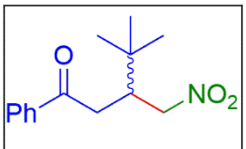

rac-4

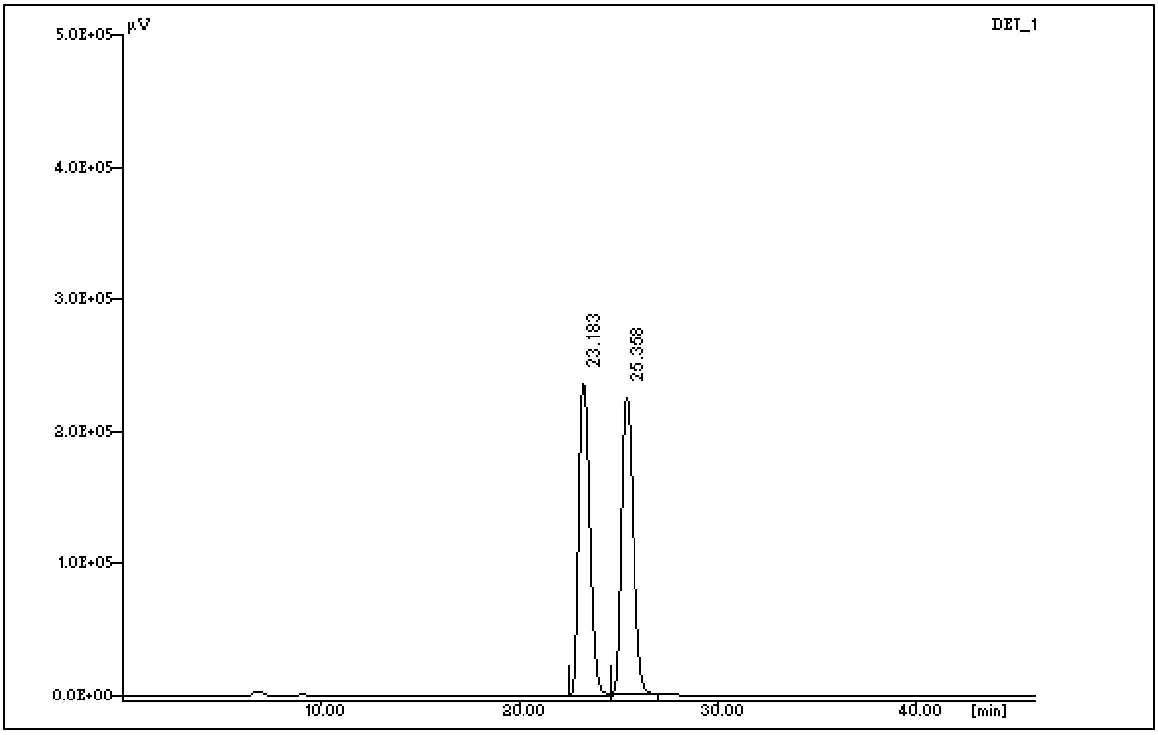

File name : AKD-409-OD-H050.CH1

Control Method :RC-1220

#	RT	%Area	Area [µV·Sec]	Height[µV]
1	23.13	48.7290	7784447.0139	235814
2	25.38	51.2710	8190516.9861	225348

Total Area of Peak = 15974964.0000 [µV·Sec]

Total Area of Signal = 15052014.0000

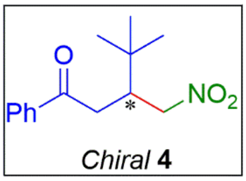

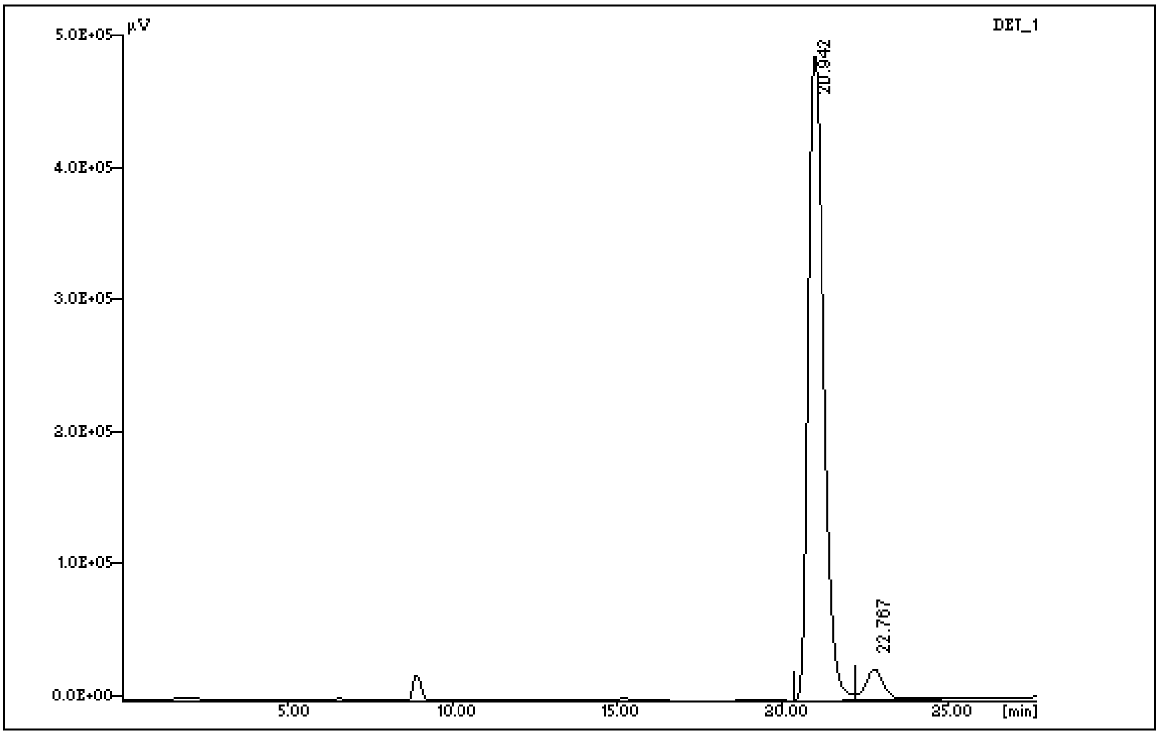

File name : AKD-473-OD-H058.CH1

Control Method :RC-1220

#	RT	%Area	Area [µV·Sec]	Height[µV]
1	20.92	94.8578	14635306.2140	488025
2	22.77	5.1422	793367.9958	22287

Total Area of Peak = 15428674.2100 [µV·Sec]

Total Area of Signal = 9965336.5000

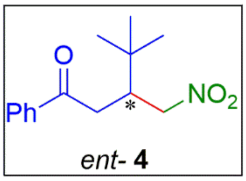

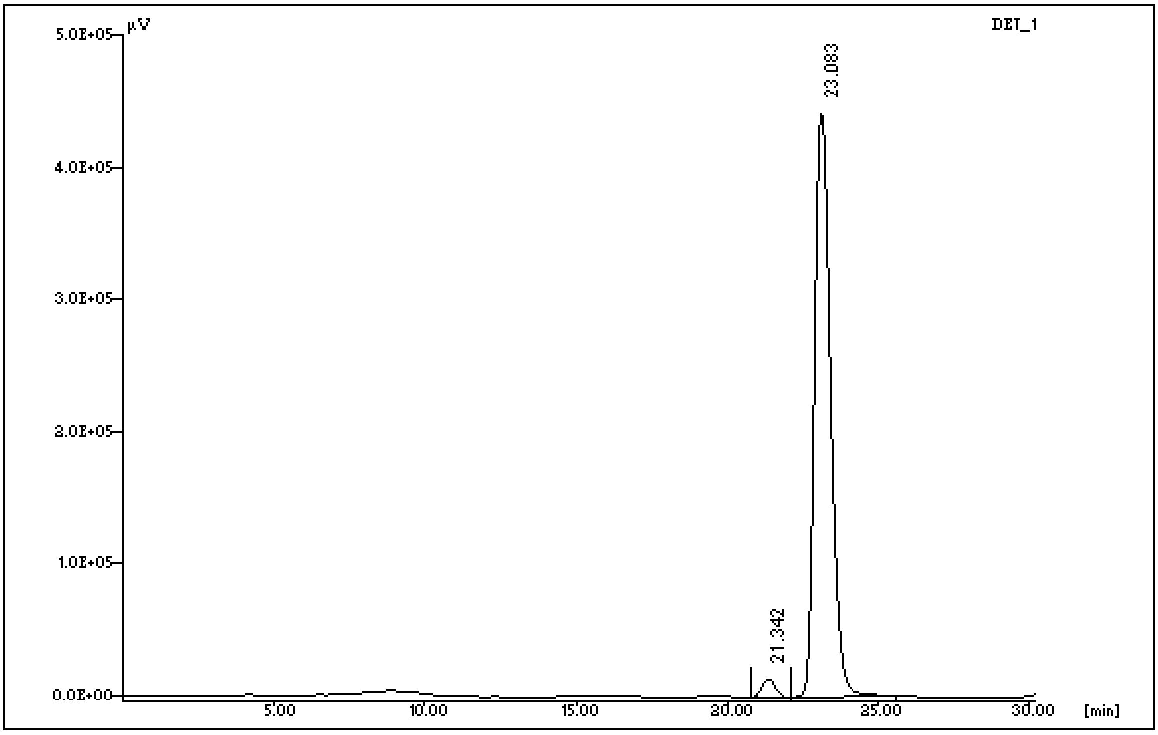

File name : AKD-412-OD-H056.CH1

Control Method :RC-1220

#	RT	%Area	Area [μV·Sec]	Height[μV]
1	21.32	2.5943	387819.0000	13701
2	23.03	97.4057	14561069.4910	440917

Total Area of Peak = 14948888.4910 [μV·Sec]

Total Area of Signal = 13738440.0000

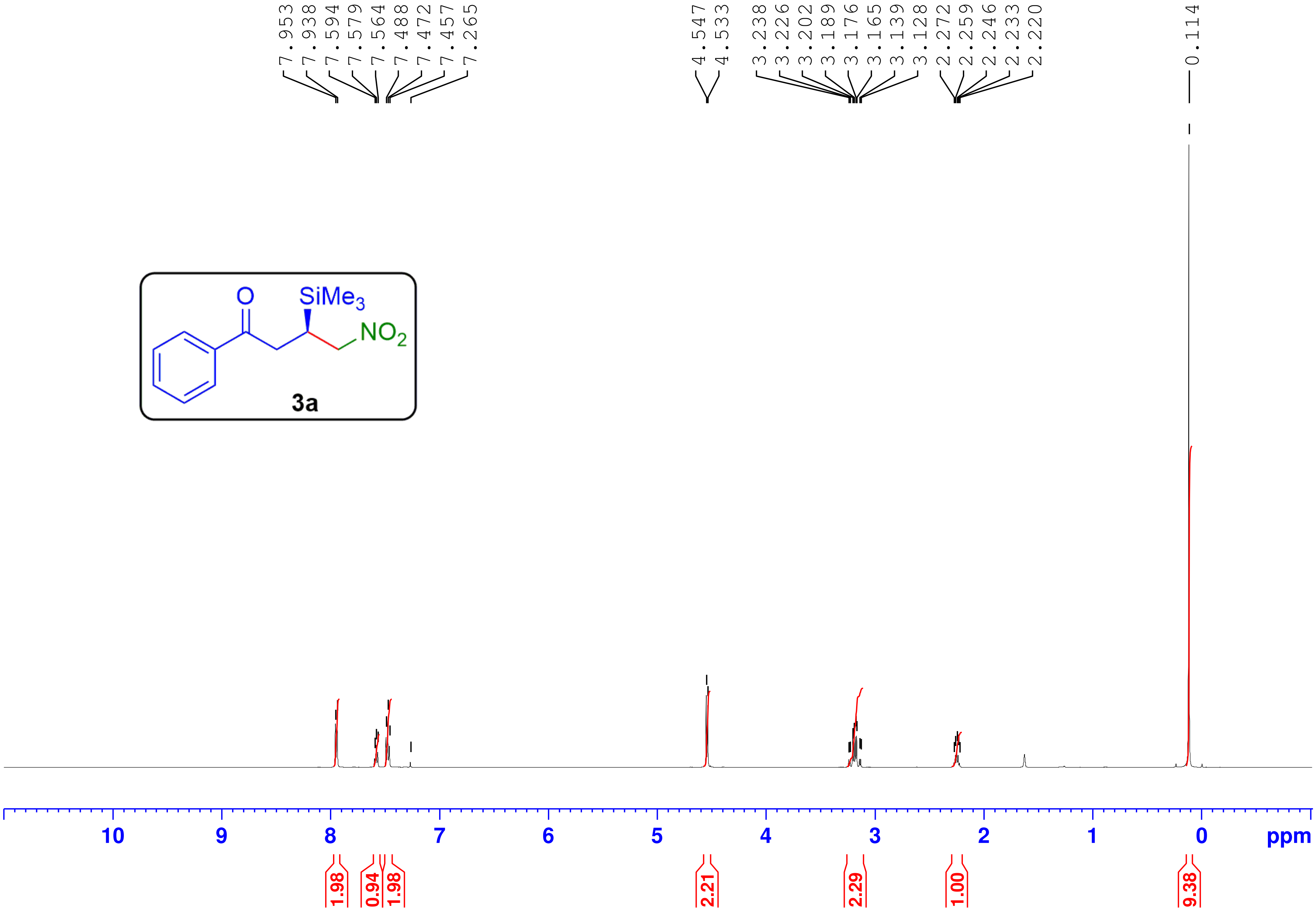

7.953
7.938
7.594
7.579
7.564
7.488
7.472
7.457
7.265
4.547
4.533
3.238
3.226
3.202
3.189
3.176
3.165
3.139
3.128
2.272
2.259
2.246
2.233
2.220
0.114
O
SiMe3
NO2
3a
10
9
8
7
6
5
4
3
2
1
0
ppm
1.98
0.94
1.98
2.21
2.29
1.00
9.38

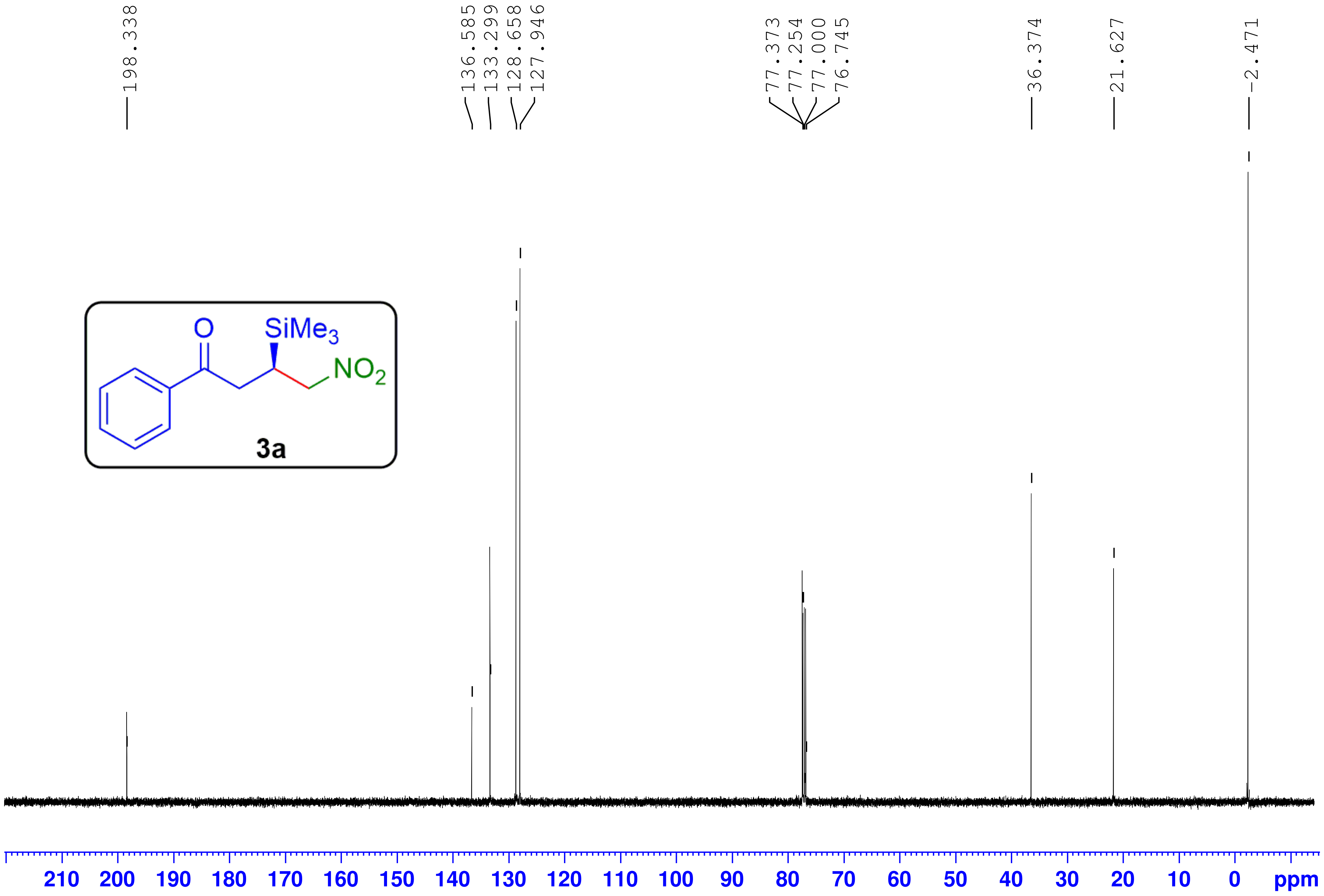
198.338
136.585
133.299
128.658
127.946
77.373
77.254
77.000
76.745
36.374
21.627
-2.471
O
SiMe3
NO2
3a
210 200 190 180 170 160 150 140 130 120 110 100 90 80 70 60 50 40 30 20 10 0 ppm

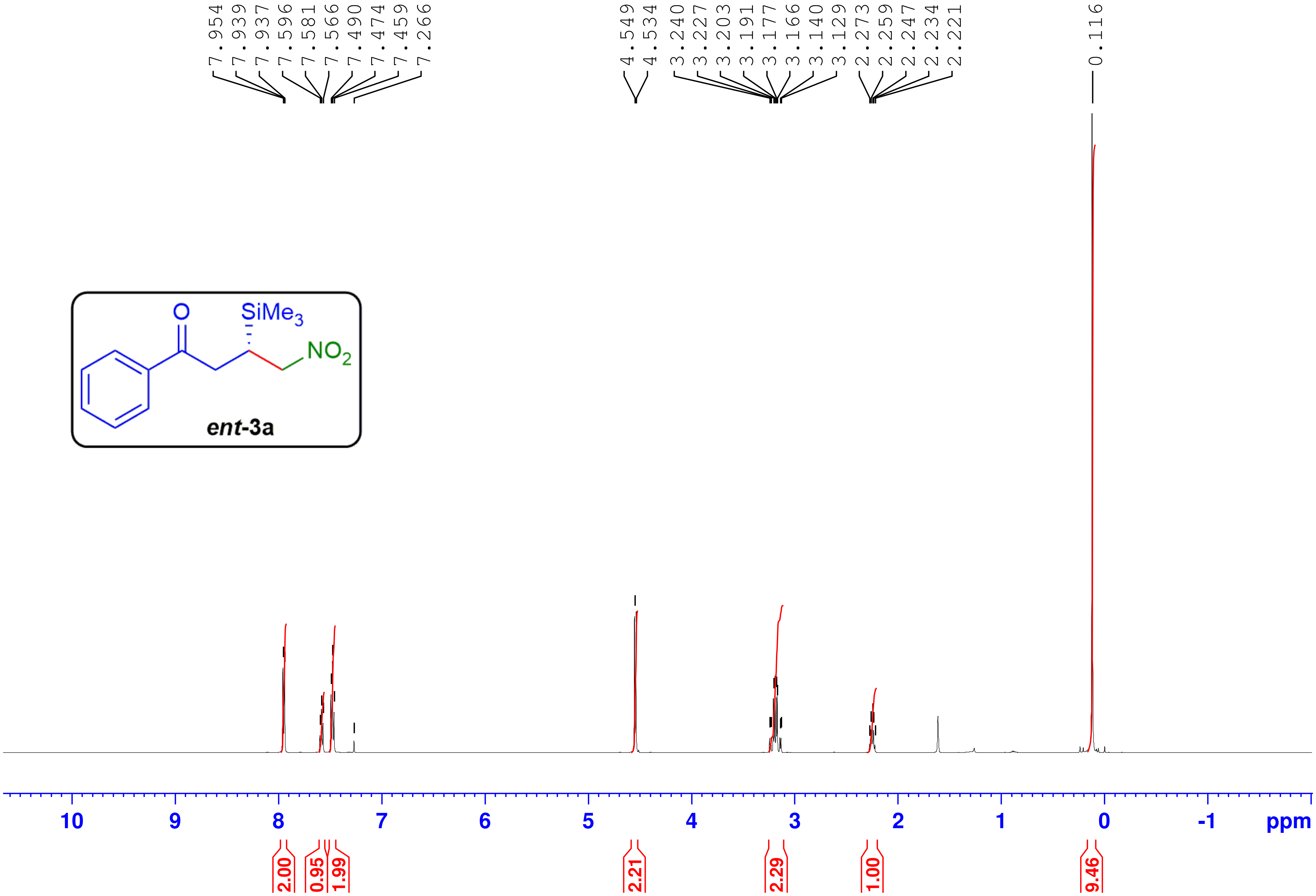
7.954
7.939
7.937
7.596
7.581
7.566
7.490
7.474
7.459
7.266
4.549
4.534
3.240
3.227
3.203
3.191
3.177
3.166
3.140
3.129
2.273
2.259
2.247
2.234
2.221
0.116
O
SiMe3
NO2
ent-3a
10
9
8
7
6
5
4
3
2
1
0
-1
ppm
2.00
0.95
1.99
2.21
2.29
1.00
9.46

S58

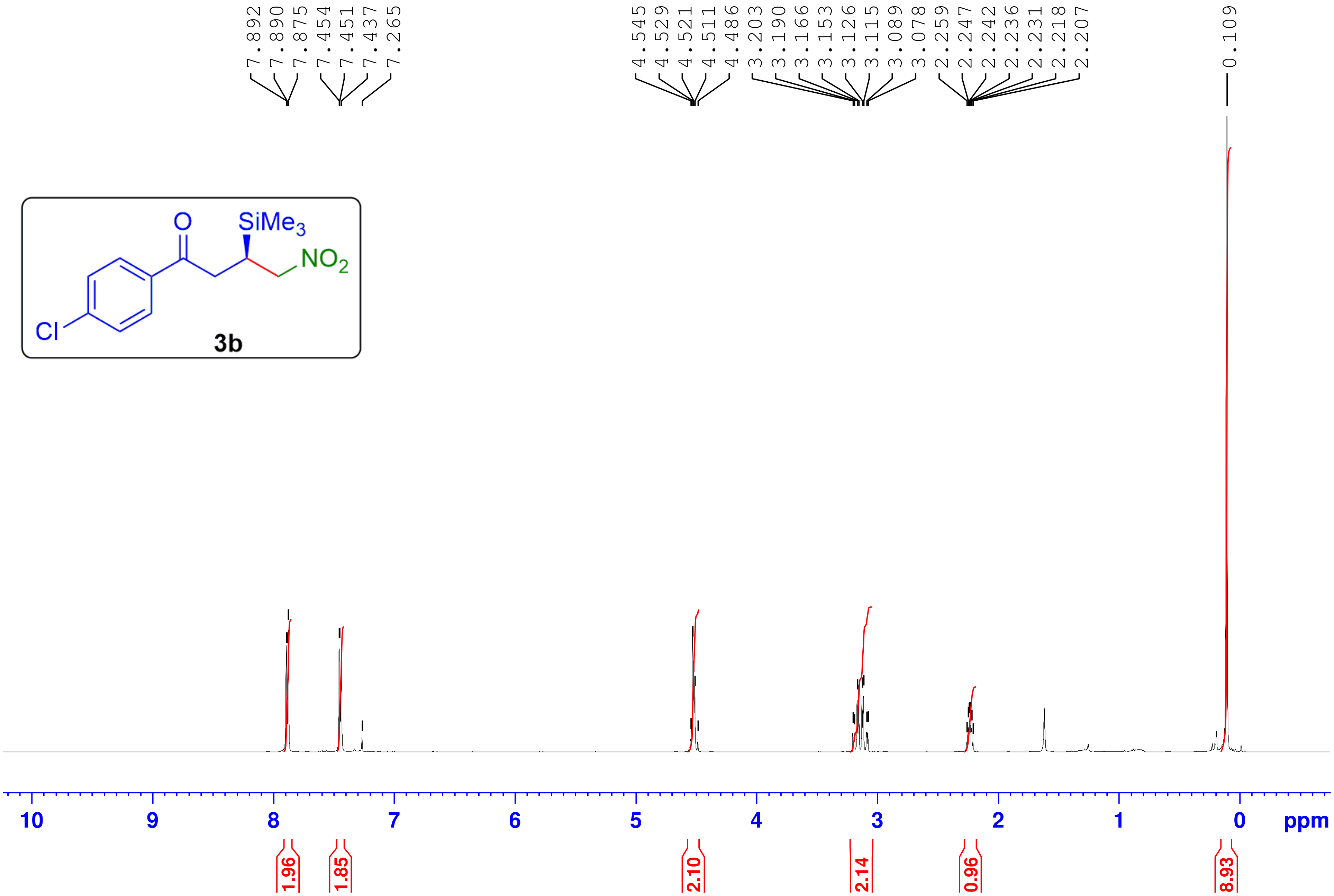
7.892
7.890
7.875
7.454
7.451
7.437
7.265
4.545
4.529
4.521
4.511
4.486
3.203
3.190
3.166
3.153
3.126
3.115
3.089
3.078
2.259
2.247
2.242
2.236
2.231
2.218
2.207
0.109
O
SiMe3
NO2
Cl
3b
10
9
8
7
6
5
4
3
2
1
0
ppm
1.96
1.85
2.10
2.14
0.96
8.93

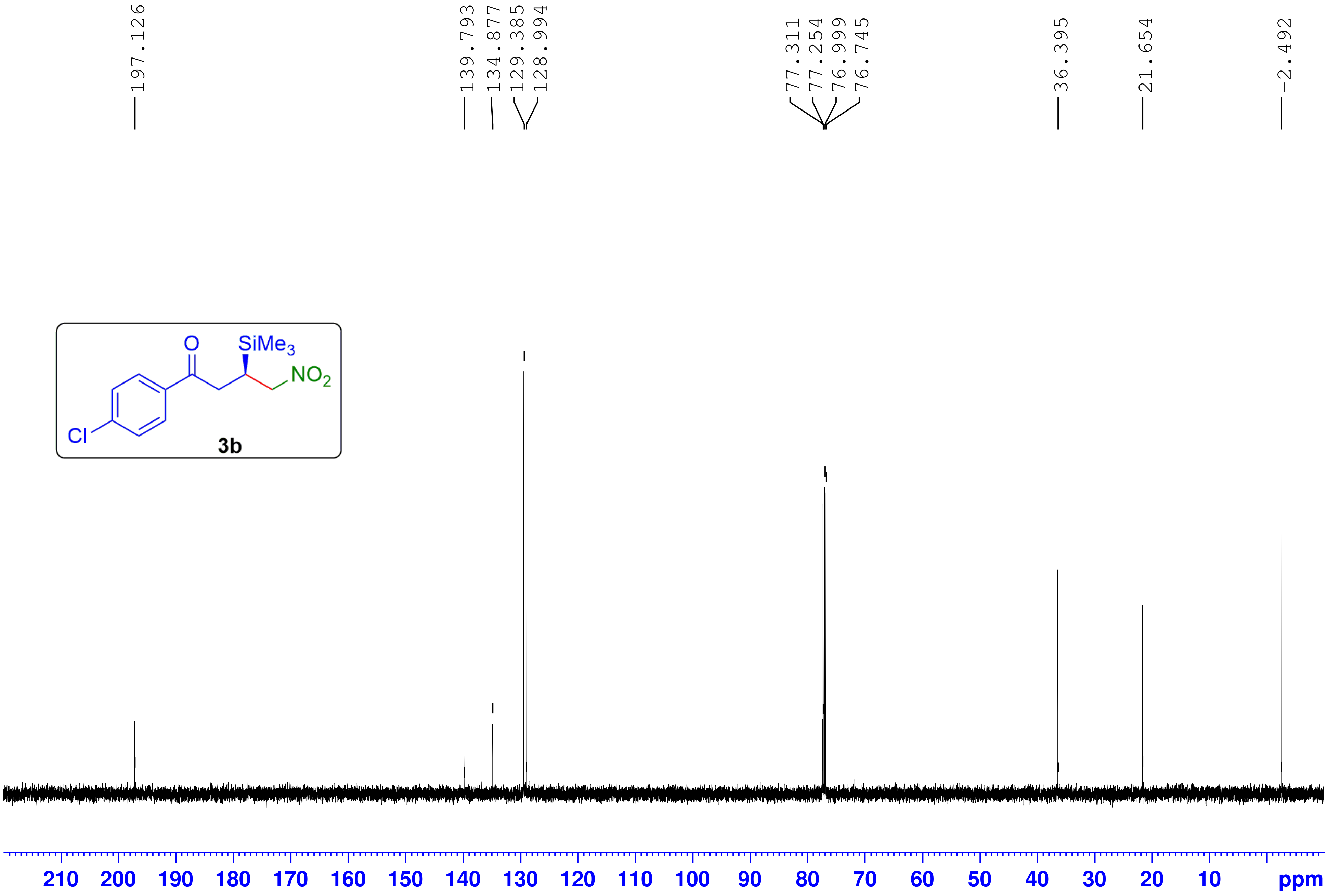
197.126
139.793
134.877
129.385
128.994
77.311
77.254
76.999
76.745
36.395
21.654
-2.492
O
SiMe3
NO2
Cl
3b
210
200
190
180
170
160
150
140
130
120
110
100
90
80
70
60
50
40
30
20
10
ppm

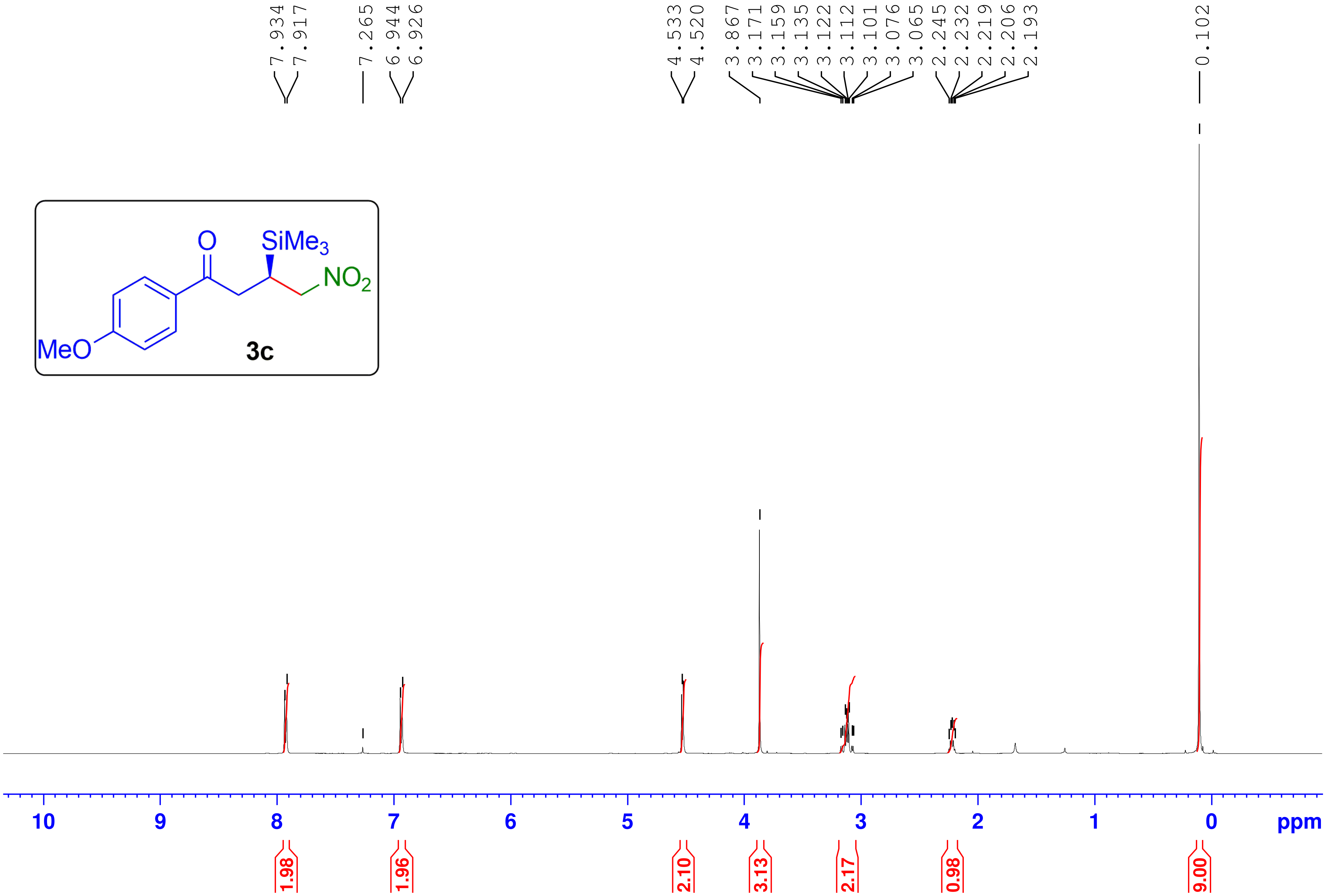
7.934
7.917
7.265
6.944
6.926
4.533
4.520
3.867
3.171
3.159
3.135
3.122
3.112
3.101
3.076
3.065
2.245
2.232
2.219
2.206
2.193
0.102
O
SiMe3
NO2
MeO
3c
10
9
8
7
6
5
4
3
2
1
0
ppm
1.98
1.96
2.10
3.13
2.17
0.98
9.00

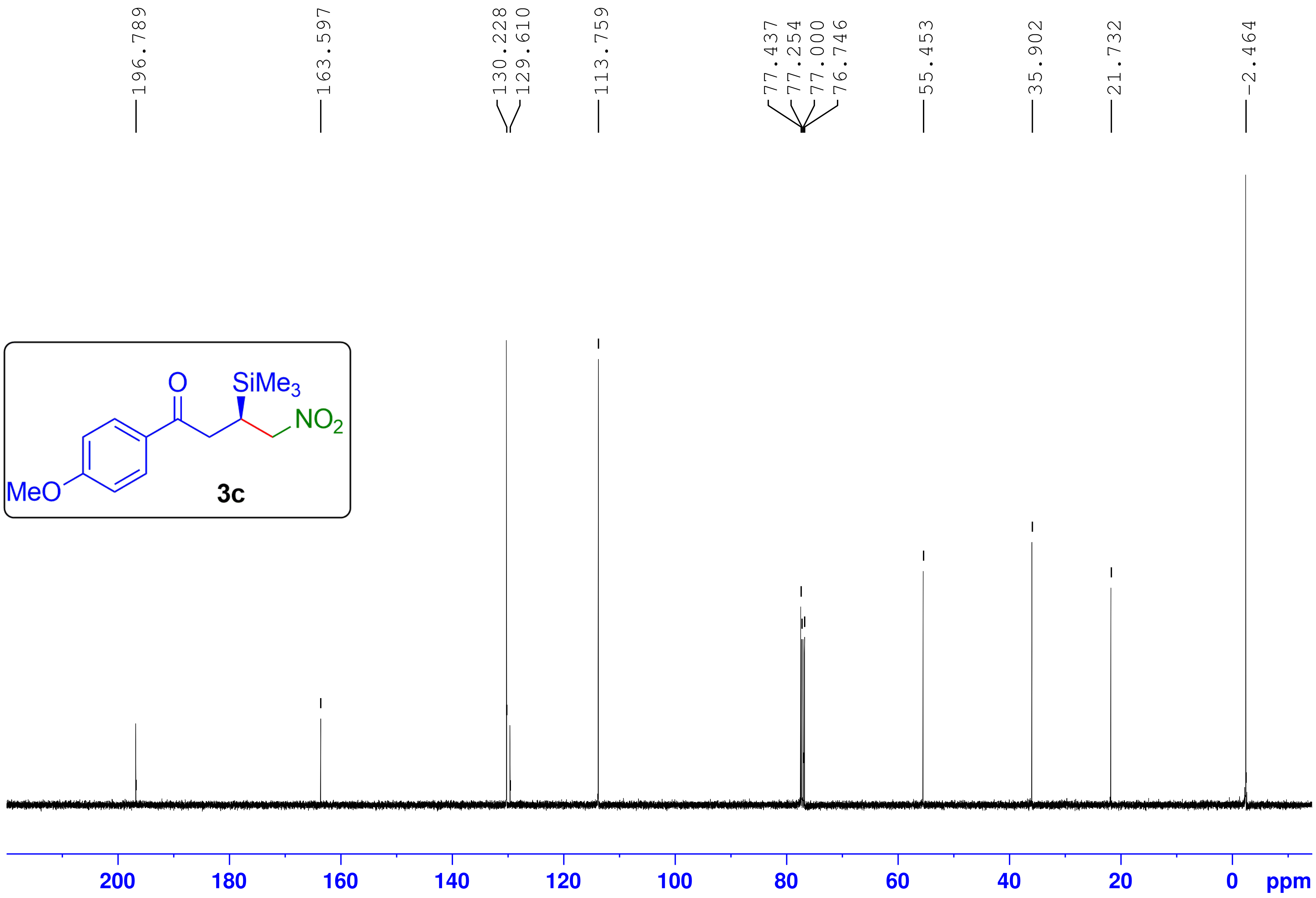

196.789
163.597
130.228
129.610
113.759
77.437
77.254
77.000
76.746
55.453
35.902
21.732
-2.464
O
SiMe3
NO2
MeO
3c
200
180
160
140
120
100
80
60
40
20
0
ppm

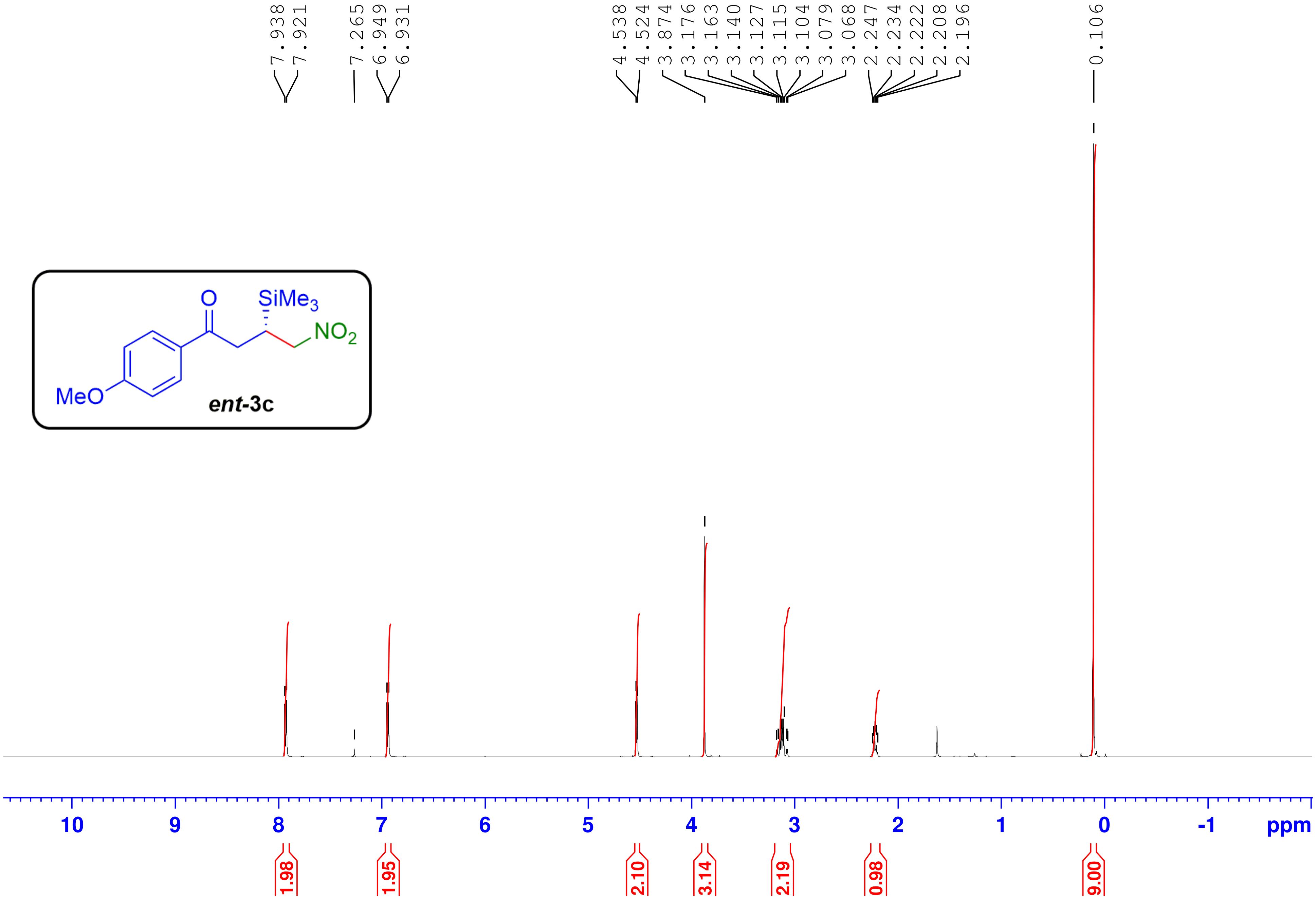
7.938
7.921
7.265
6.949
6.931
4.538
4.524
3.874
3.176
3.163
3.140
3.127
3.115
3.104
3.079
3.068
2.247
2.234
2.222
2.208
2.196
0.106
O
SiMe3
NO2
MeO
ent-3c
10
9
8
7
6
5
4
3
2
1
0
-1
ppm
1.98
1.95
2.10
3.14
2.19
0.98
9.00

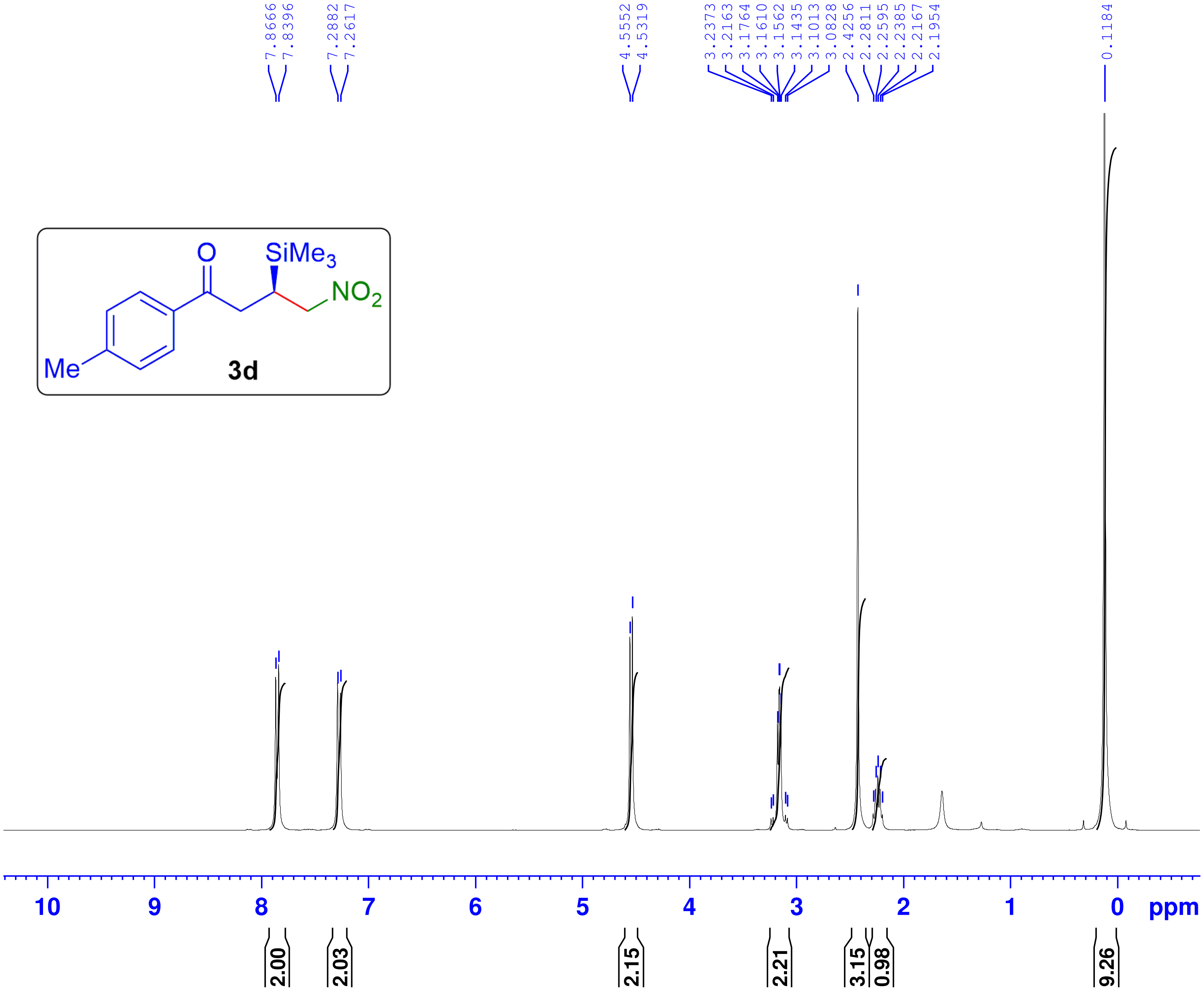

BRUKER

```
Current Data Parameters
NAME     akd 399 and akd 400 date12042021
EXPNO                  9
PROCNO                 1

F2 - Acquisition Parameters
Date_           20210412
Time               14.40
INSTRUM            spect
PROBHD   5 mm PABBO BB-
PULPROG               zg
TD                 32768
SOLVENT            CDCl3
NS                    16
DS                     0
SWH             4504.504 Hz
FIDRES          0.137467 Hz
AQ             3.6372480 sec
RG                  80.6
DW               111.000 usec
DE                  6.50 usec
TE                 299.7 K
D1            2.00000000 sec
TD0                    1

======== CHANNEL f1 ========
NUC1                  1H
P1                 13.50 usec
PL1                -0.30 dB
PL1W         14.53428841 W
SFO1         300.1318008 MHz

F2 - Processing parameters
SI                 16384
SF           300.1300027 MHz
WDW                   EM
SSB      0
LB                  1.00 Hz
GB       0
PC                  1.00
```

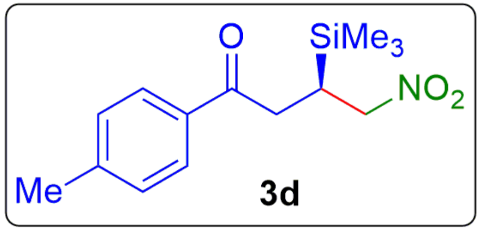

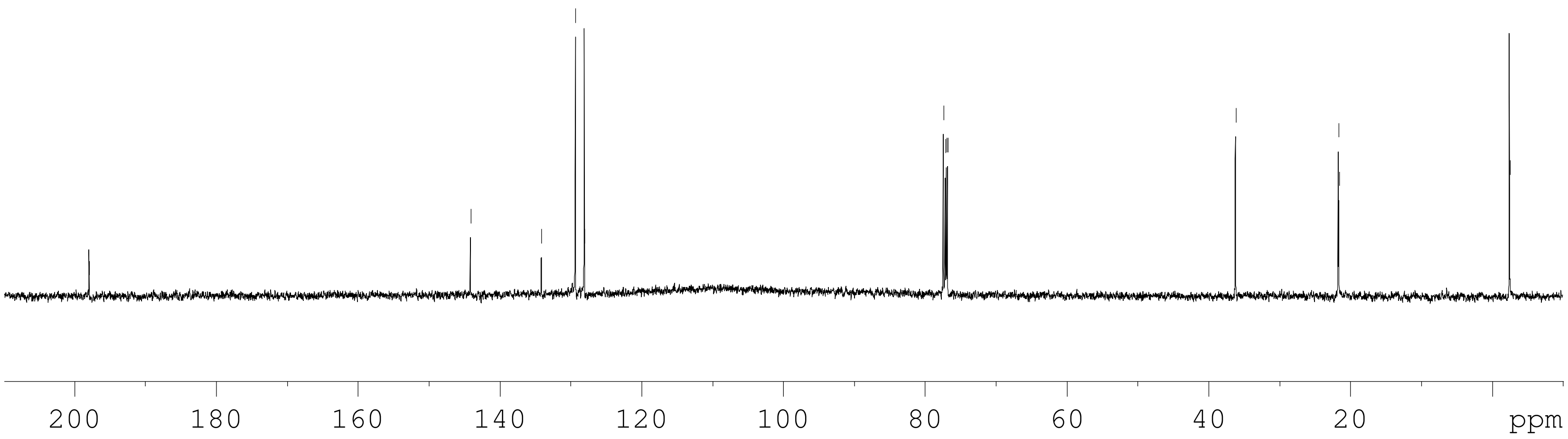

Current Data Parameters
NAME barc-akhil-13C-29Sep20
EXPNO 6
PROCNO 1

F2 - Acquisition Parameters

Date_	20200929
Time	14.29 h
INSTRUM	spect
PROBHD	Z555801_0012 (
PULPROG	zgdc
TD	16384
SOLVENT	CDCl3
NS	55
DS	4
SWH	44247.789 Hz
FIDRES	5.401341 Hz
AQ	0.1851392 sec
RG	71.8
DW	11.300 usec
DE	6.50 usec
TE	298.0 K
D1	3.00000000 sec
D11	0.03000000 sec
TD0	1
SFO1	201.1878208 MHz
NUC1	13C
P1	11.00 usec
PLW1	312.79998779 W
SFO2	800.0332001 MHz
NUC2	1H
CPDPRG[2	waltz16
PCPD2	60.00 usec
PLW2	13.00000000 W
PLW12	0.29249999 W

F2 - Processing parameters

SI	16384
SF	201.1677168 MHz
WDW	EM
SSB	0
LB	5.00 Hz
GB	0
PC	1.40

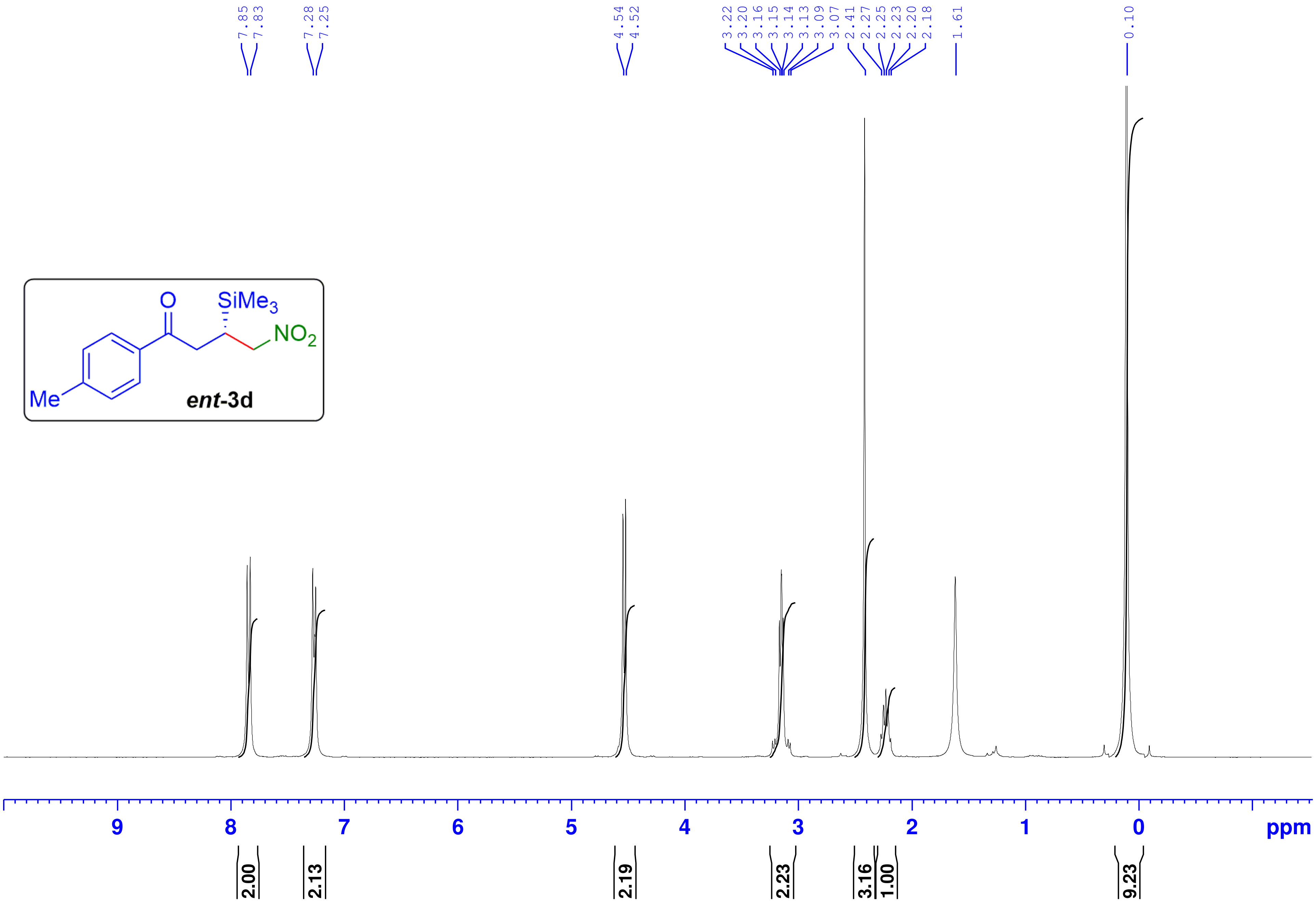
7.85
7.83
7.28
7.25
4.54
4.52
3.22
3.20
3.16
3.15
3.14
3.13
3.09
3.07
2.41
2.27
2.25
2.23
2.20
2.18
1.61
0.10
O
SiMe3
NO2
Me
ent-3d
9
8
7
6
5
4
3
2
1
0
ppm
2.00
2.13
2.19
2.23
3.16
1.00
9.23

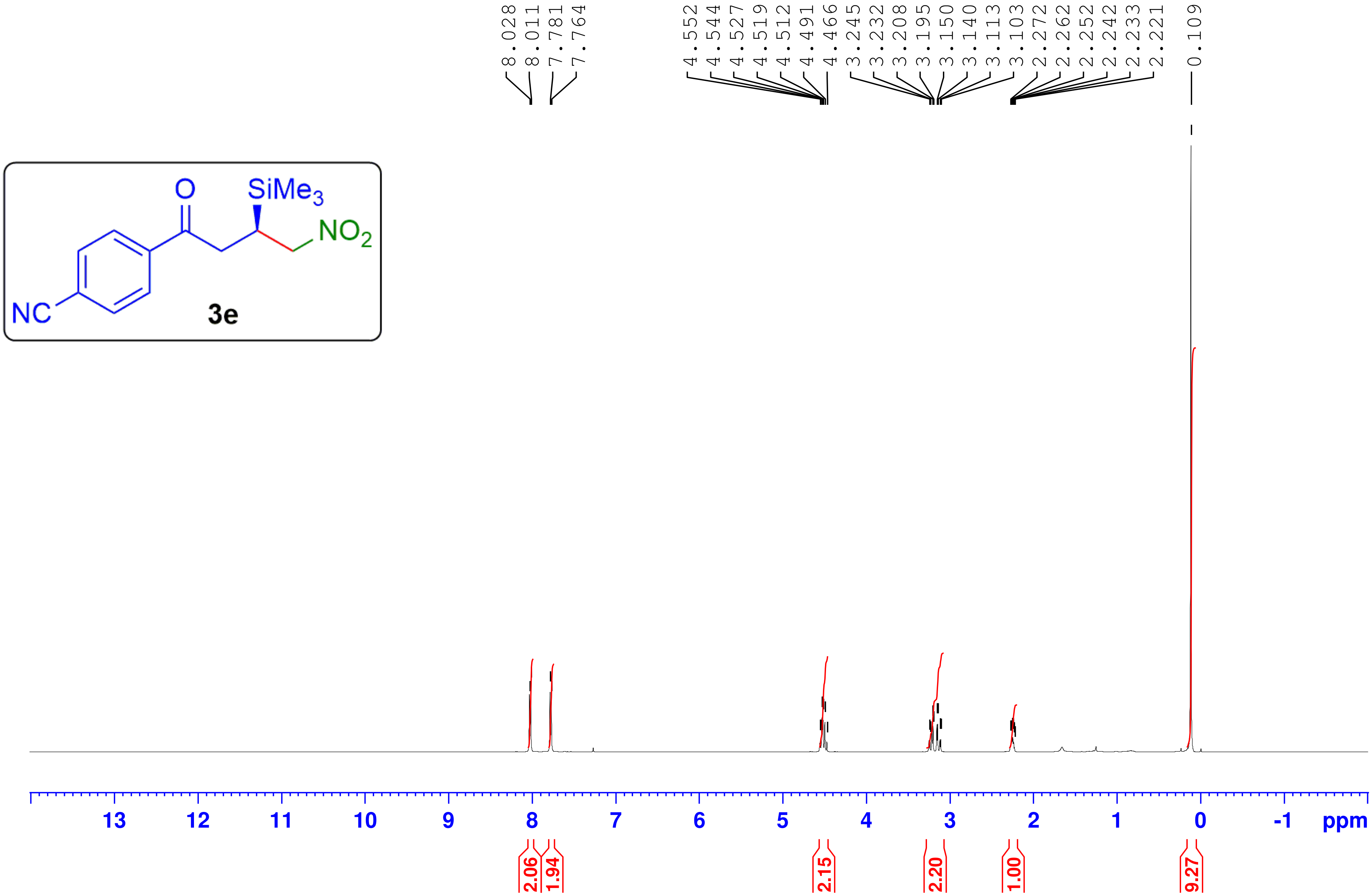

8.028
8.011
7.781
7.764
4.552
4.544
4.527
4.519
4.512
4.491
4.466
3.245
3.232
3.208
3.195
3.150
3.140
3.113
3.103
2.272
2.262
2.252
2.242
2.233
2.221
0.109
O
SiMe3
NO2
NC
3e
13
12
11
10
9
8
7
6
5
4
3
2
1
0
-1
ppm
2.06
1.94
2.15
2.20
1.00
9.27

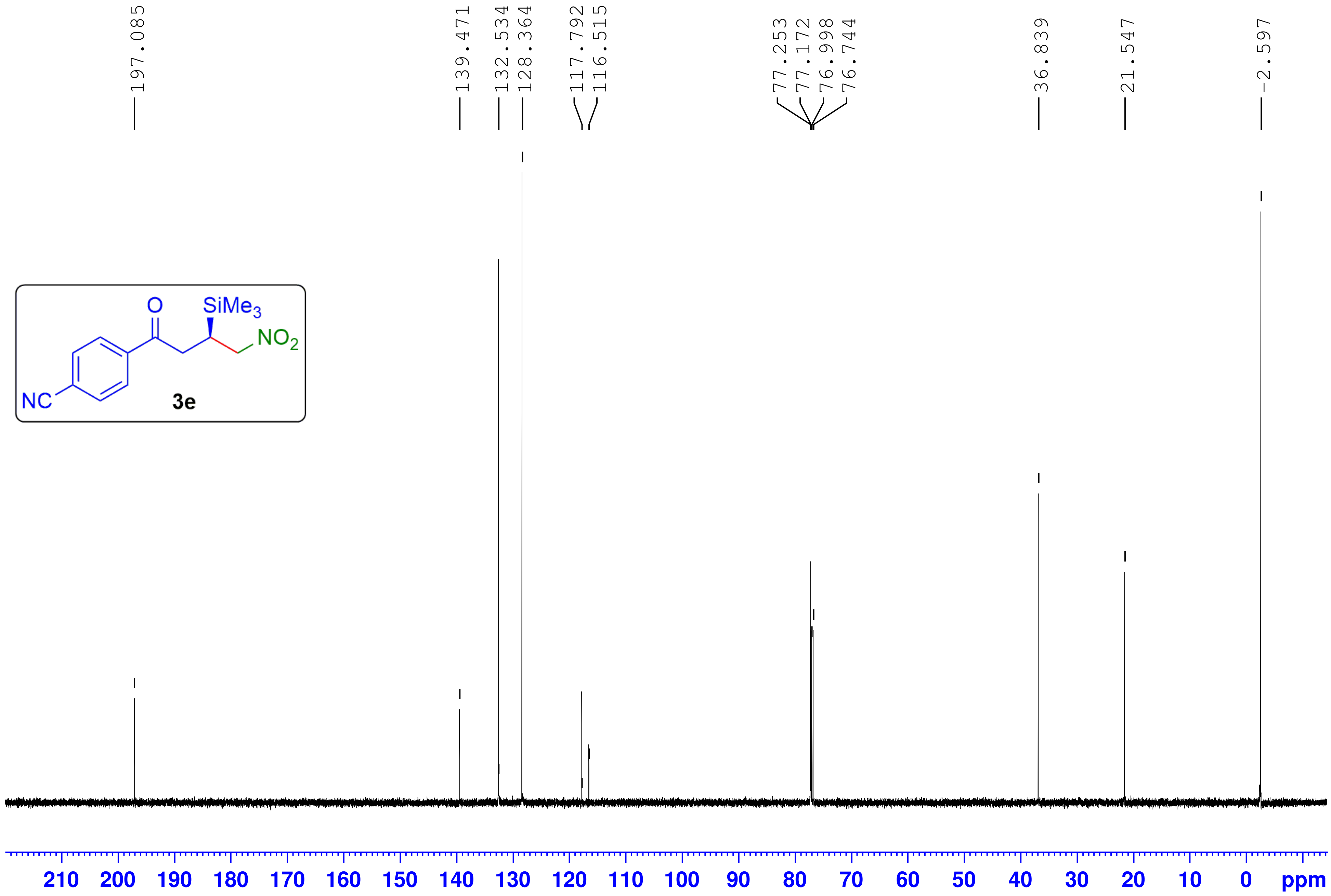
197.085
139.471
132.534
128.364
117.792
116.515
77.253
77.172
76.998
76.744
36.839
21.547
-2.597
O
SiMe3
NO2
NC
3e
210 200 190 180 170 160 150 140 130 120 110 100 90 80 70 60 50 40 30 20 10 0 ppm

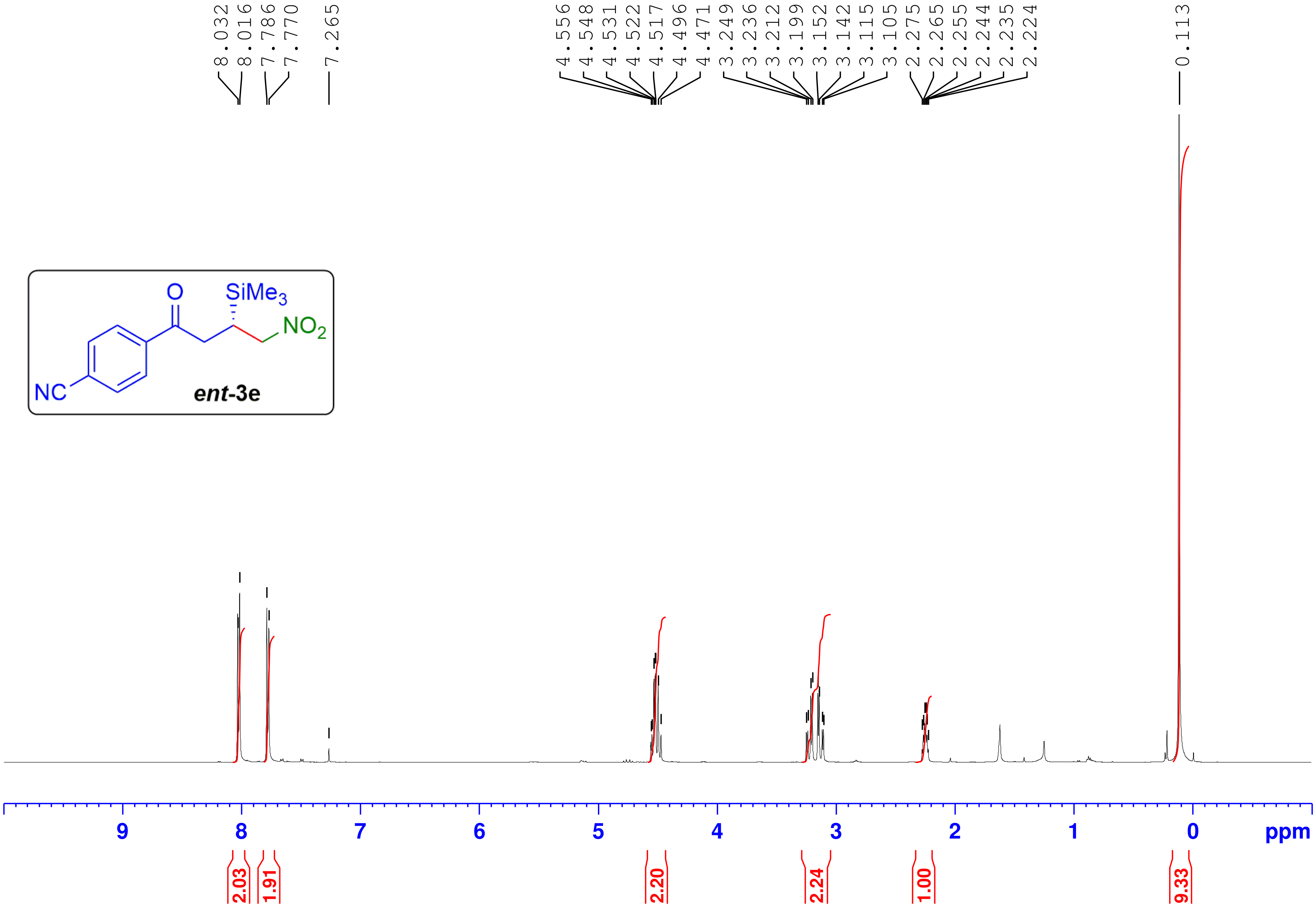

8.032
8.016
7.786
7.770
7.265
4.556
4.548
4.531
4.522
4.517
4.496
4.471
3.249
3.236
3.212
3.199
3.152
3.142
3.115
3.105
2.275
2.265
2.255
2.244
2.235
2.224
0.113
O
SiMe3
NO2
NC
ent-3e
9
8
7
6
5
4
3
2
1
0
ppm
2.03
1.91
2.20
2.24
1.00
9.33

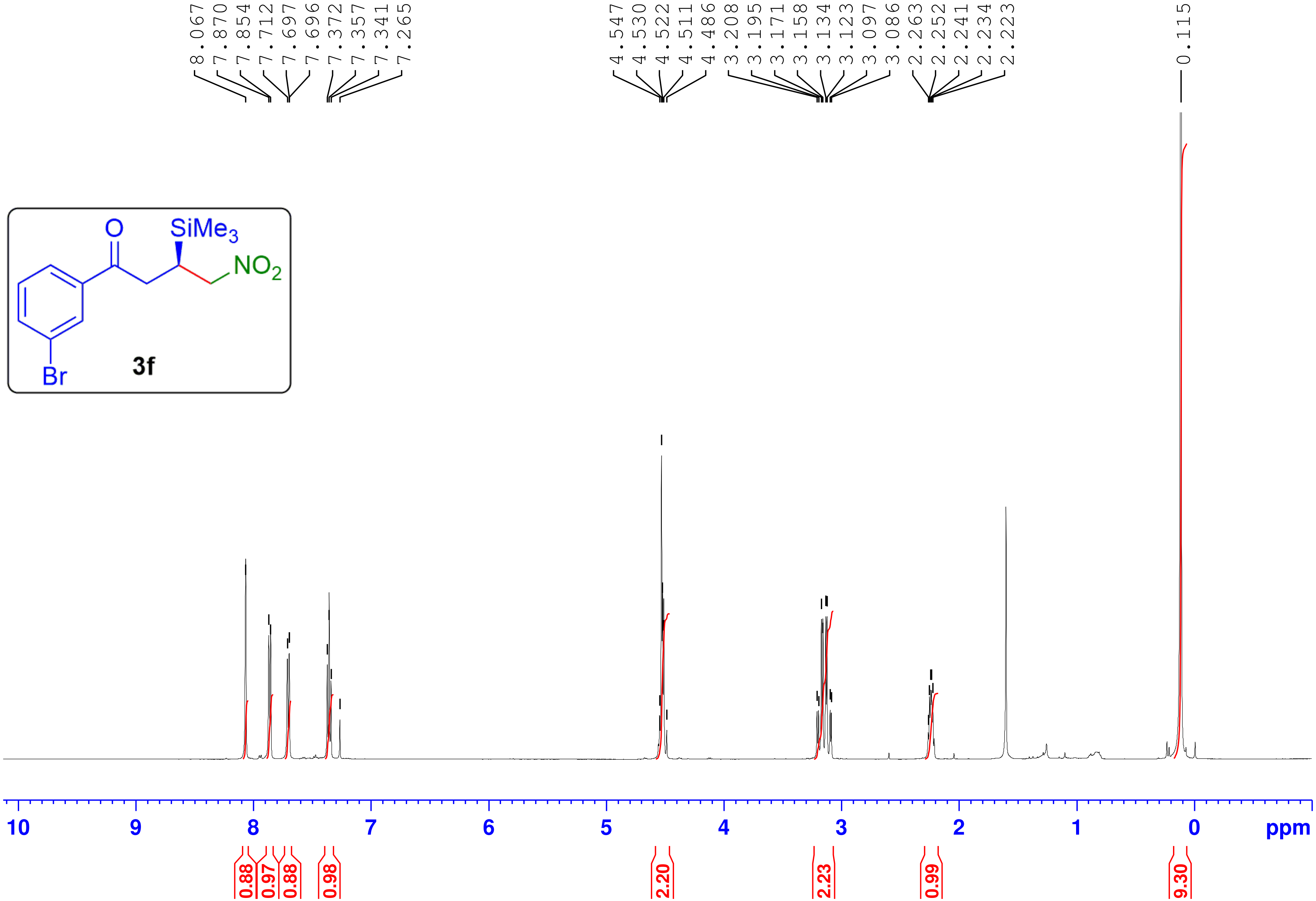
8.067
7.870
7.854
7.712
7.697
7.696
7.372
7.357
7.341
7.265
4.547
4.530
4.522
4.511
4.486
3.208
3.195
3.171
3.158
3.134
3.123
3.097
3.086
2.263
2.252
2.241
2.234
2.223
0.115
O
SiMe3
NO2
Br
3f
10
9
8
7
6
5
4
3
2
1
0
ppm
0.88
0.97
0.88
0.98
2.20
2.23
0.99
9.30

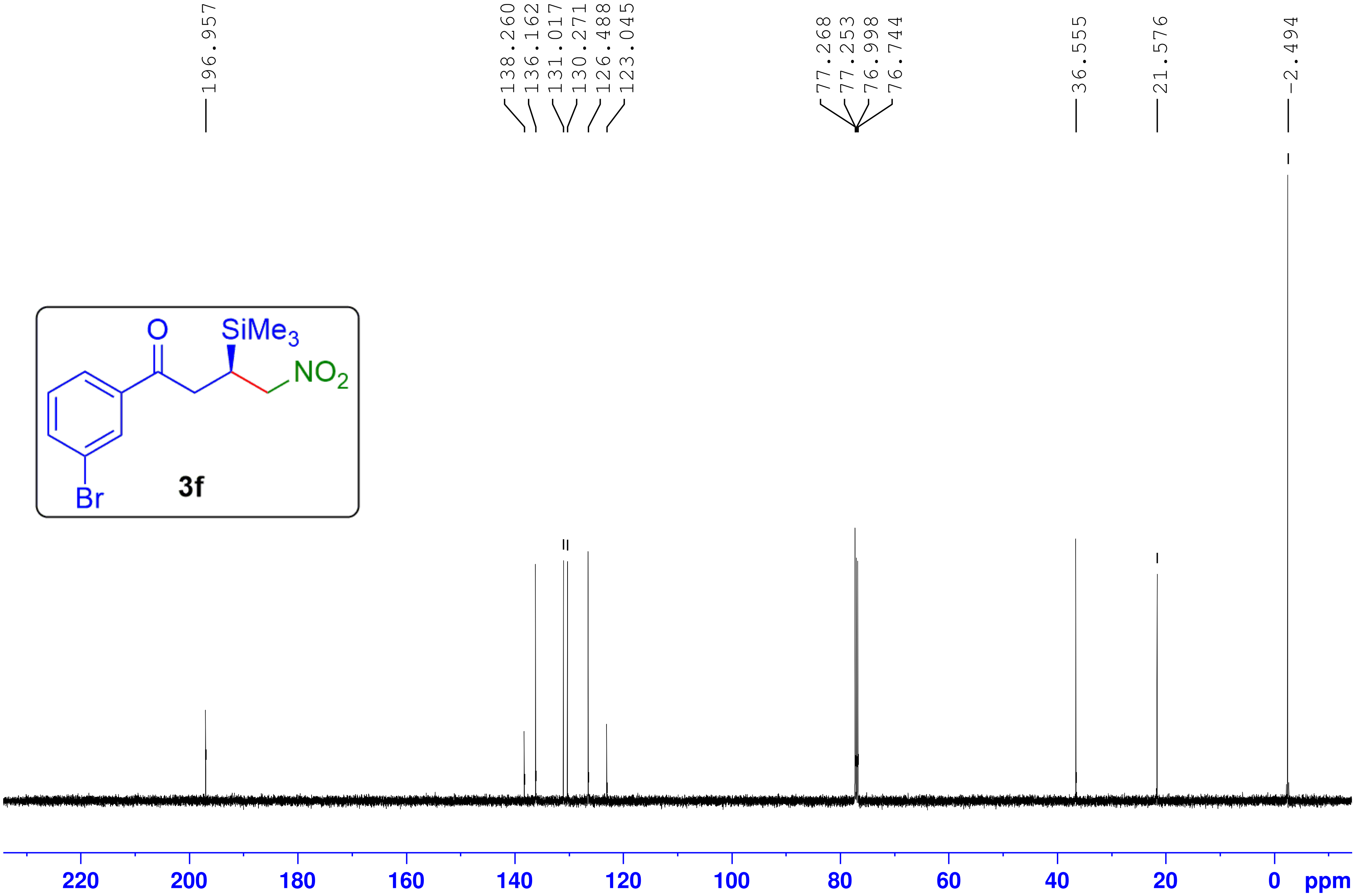

196.957
138.260
136.162
131.017
130.271
126.488
123.045
77.268
77.253
76.998
76.744
36.555
21.576
-2.494
O
SiMe3
NO2
Br
3f
220
200
180
160
140
120
100
80
60
40
20
0
ppm

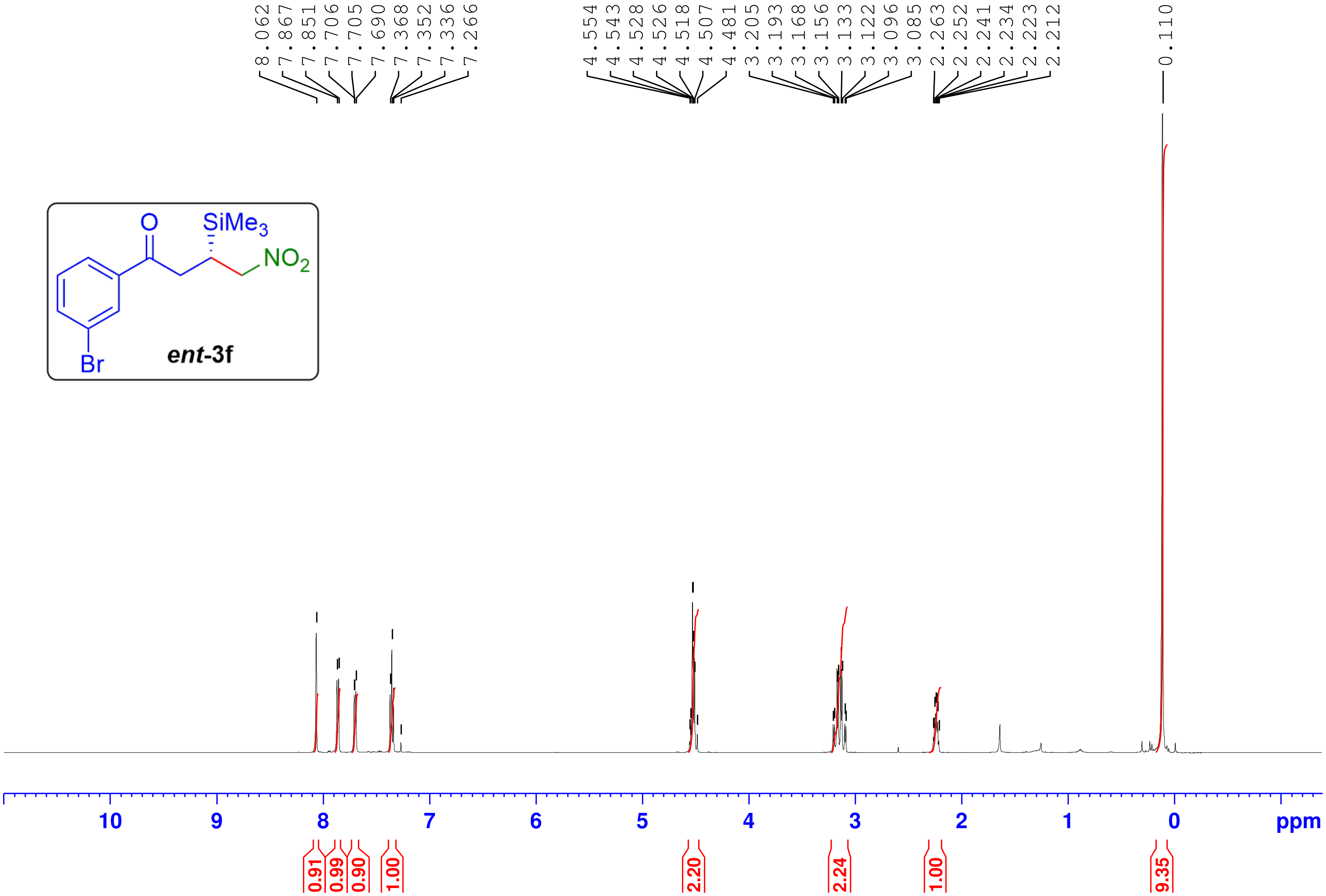
8.062
7.867
7.851
7.706
7.705
7.690
7.368
7.352
7.336
7.266
4.554
4.543
4.528
4.526
4.518
4.507
4.481
3.205
3.193
3.168
3.156
3.133
3.122
3.096
3.085
2.263
2.252
2.241
2.234
2.223
2.212
0.110
O
SiMe3
NO2
Br
ent-3f
10
9
8
7
6
5
4
3
2
1
0
ppm
0.91
0.99
0.90
1.00
2.20
2.24
1.00
9.35

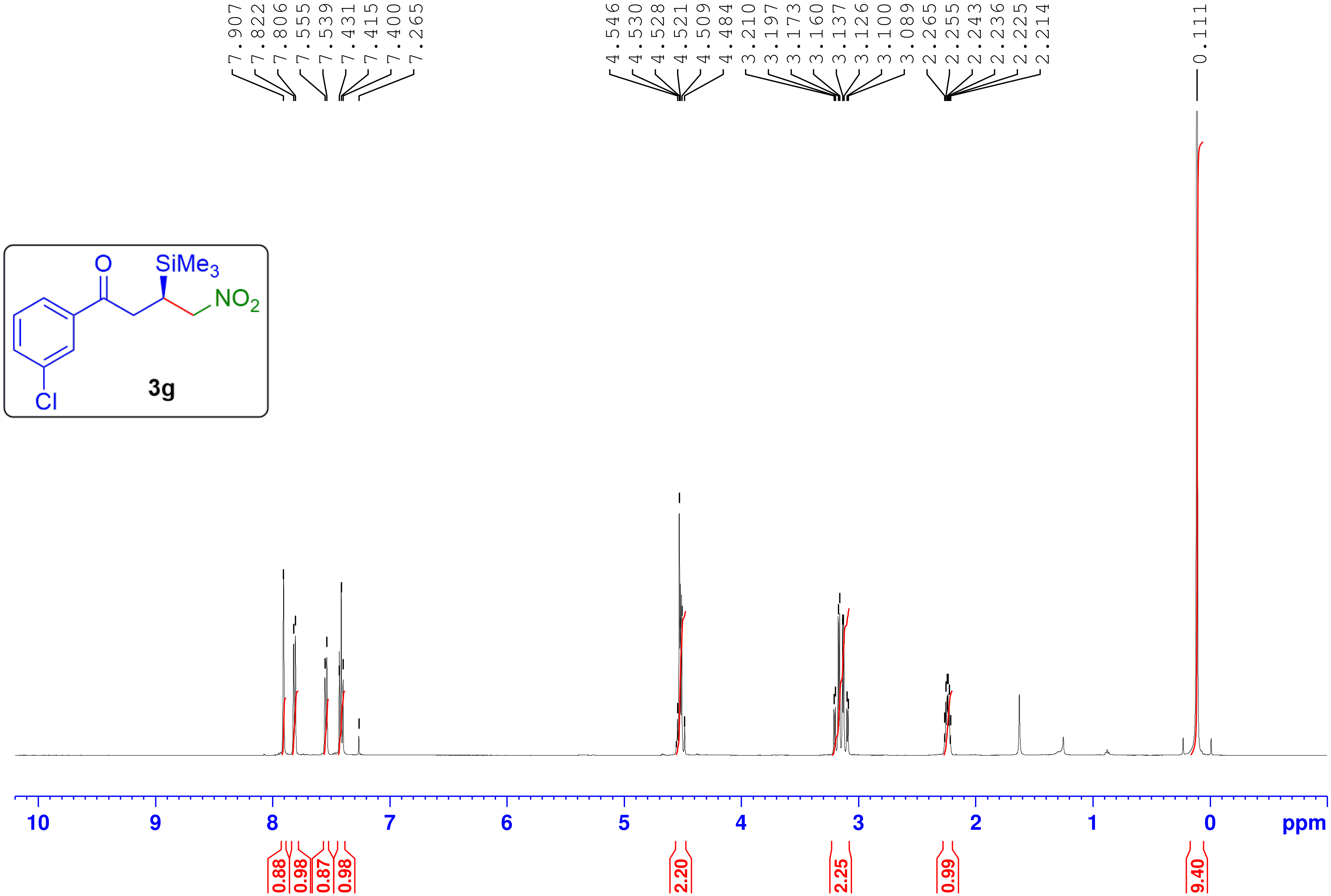
O
SiMe3
NO2
Cl
3g
7.907
7.822
7.806
7.555
7.539
7.431
7.415
7.400
7.265
4.546
4.530
4.528
4.521
4.509
4.484
3.210
3.197
3.173
3.160
3.137
3.126
3.100
3.089
2.265
2.255
2.243
2.236
2.225
2.214
0.111
10
9
8
7
6
5
4
3
2
1
0
ppm
0.88
0.98
0.87
0.98
2.20
2.25
0.99
9.40

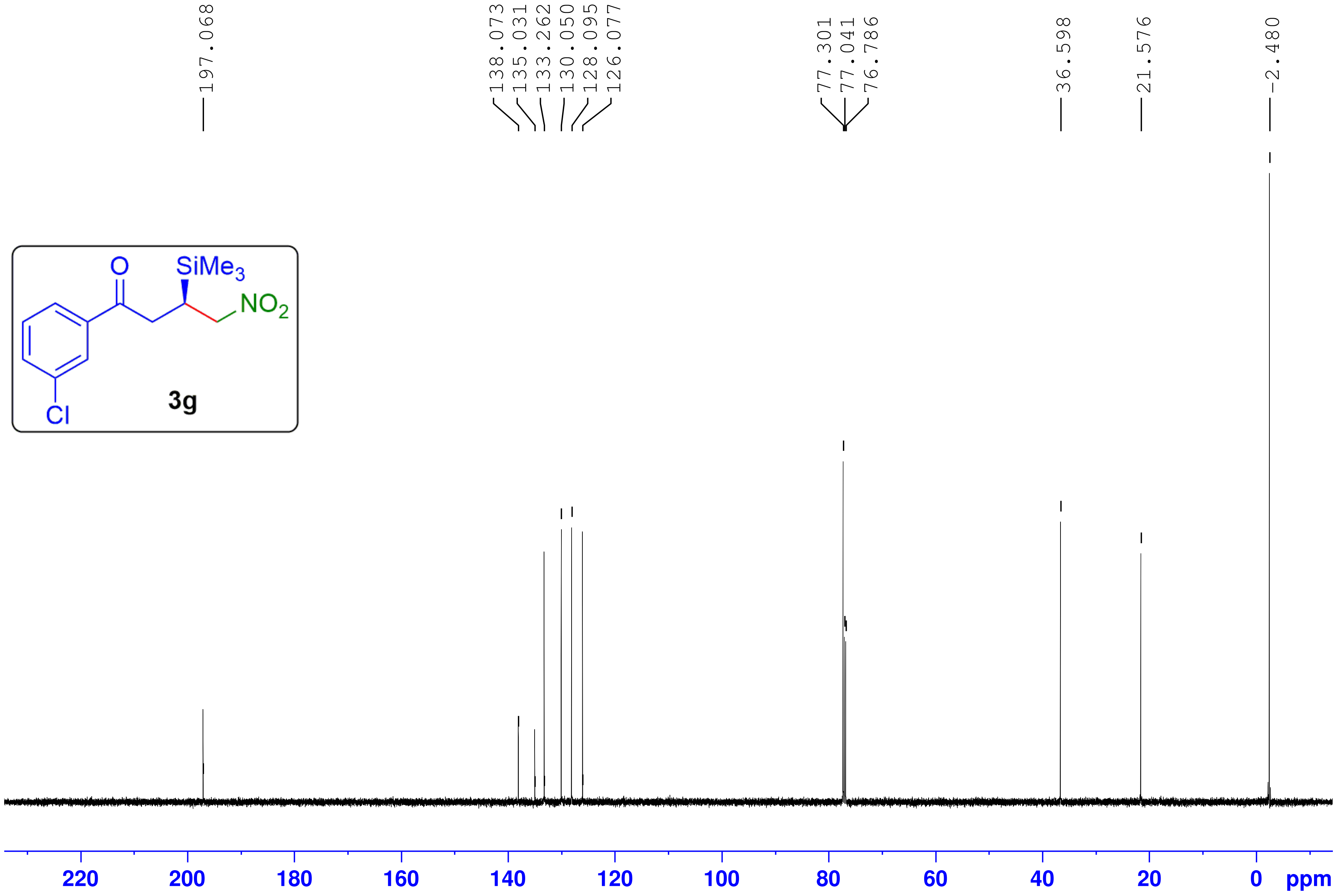

197.068
138.073
135.031
133.262
130.050
128.095
126.077
77.301
77.041
76.786
36.598
21.576
-2.480
O
SiMe3
NO2
Cl
3g
220
200
180
160
140
120
100
80
60
40
20
0
ppm

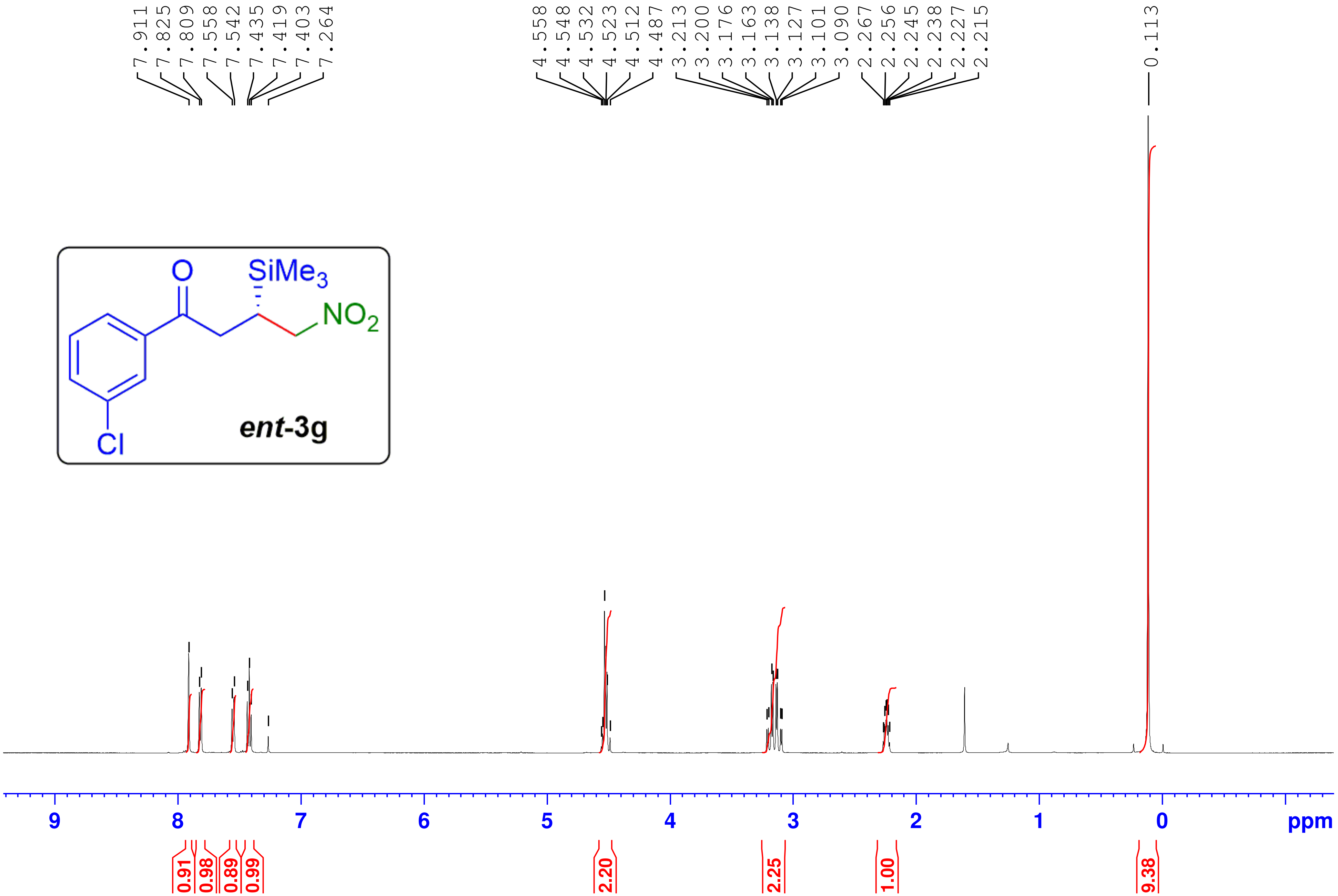
7.911
7.825
7.809
7.558
7.542
7.435
7.419
7.403
7.264
4.558
4.548
4.532
4.523
4.512
4.487
3.213
3.200
3.176
3.163
3.138
3.127
3.101
3.090
2.267
2.256
2.245
2.238
2.227
2.215
0.113
O
SiMe3
NO2
Cl
ent-3g
9
8
7
6
5
4
3
2
1
0
ppm
0.91
0.98
0.89
0.99
2.20
2.25
1.00
9.38

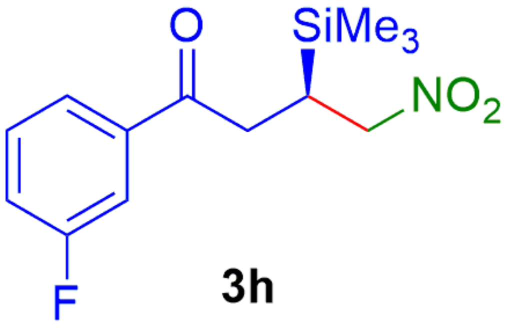

3h

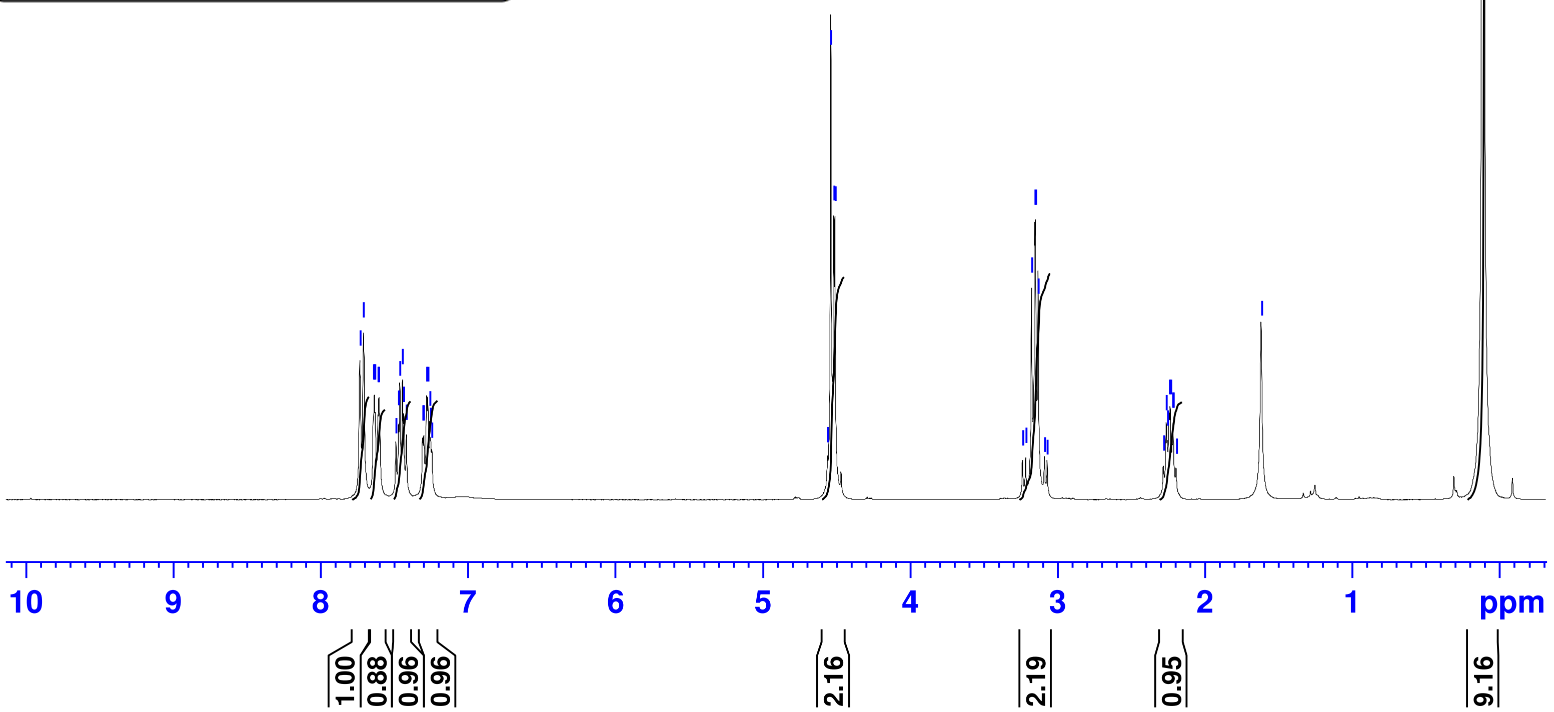

Current Data Parameters
NAME nmr 403 404
EXPNO 12
PROCNO 1

F2 - Acquisition Parameters
Date_ 20210715
Time 10.22
INSTRUM spect
PROBHD 5 mm PABBO BB-
PULPROG zg
TD 32768
SOLVENT CDCl3
NS 16
DS 0
SWH 4504.504 Hz
FIDRES 0.137467 Hz
AQ 3.6372480 sec
RG 80.6
DW 111.000 usec
DE 6.50 usec
TE 296.9 K
D1 2.00000000 sec
TD0 1

======== CHANNEL f1 ========
NUC1 1H
P1 13.50 usec
PL1 -0.30 dB
PL1W 14.53428841 W
SFO1 300.1318008 MHz

F2 - Processing parameters
SI 16384
SF 300.1300070 MHz
WDW EM
SSB 0
LB 1.00 Hz
GB 0
PC 1.00

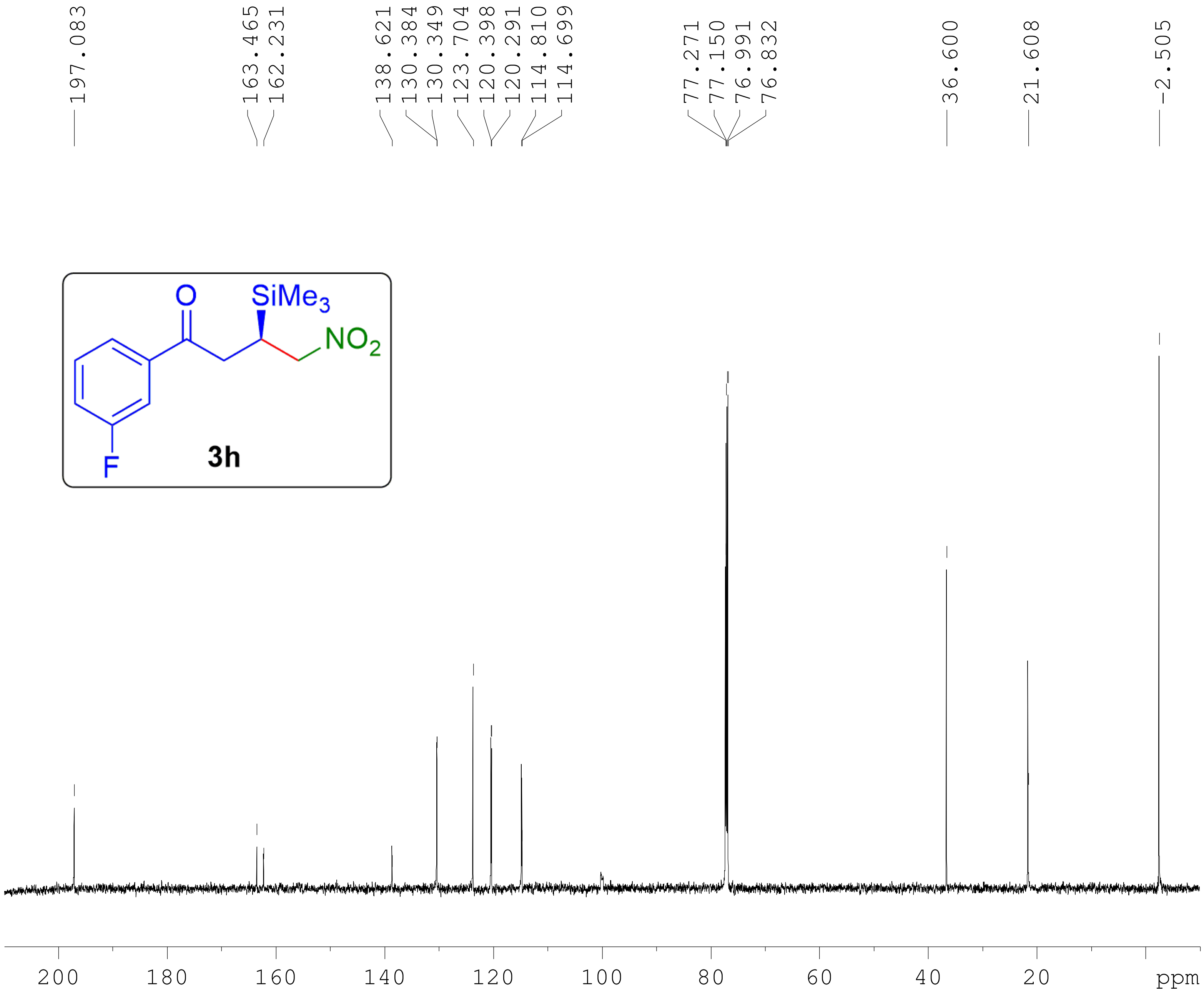

Current Data Parameters

NAME	barc-akhil-13C-29Sep20
EXPNO	9
PROCNO	1

F2 - Acquisition Parameters

Date_	20200929
Time	15.50 h
INSTRUM	spect
PROBHD	Z555801_0012 (
PULPROG	zgdc
TD	16384
SOLVENT	CDCl3
NS	436
DS	4
SWH	44247.789 Hz
FIDRES	5.401341 Hz
AQ	0.1851392 sec
RG	71.8
DW	11.300 usec
DE	6.50 usec
TE	298.0 K
D1	3.00000000 sec
D11	0.03000000 sec
TD0	1
SFO1	201.1878208 MHz
NUC1	13C
P1	11.00 usec
PLW1	312.79998779 W
SFO2	800.0332001 MHz
NUC2	1H
CPDPRG[2	waltz16
PCPD2	60.00 usec
PLW2	13.00000000 W
PLW12	0.29249999 W

F2 - Processing parameters

SI	16384
SF	201.1677099 MHz
WDW	EM
SSB	0
LB	5.00 Hz
GB	0
PC	1.40

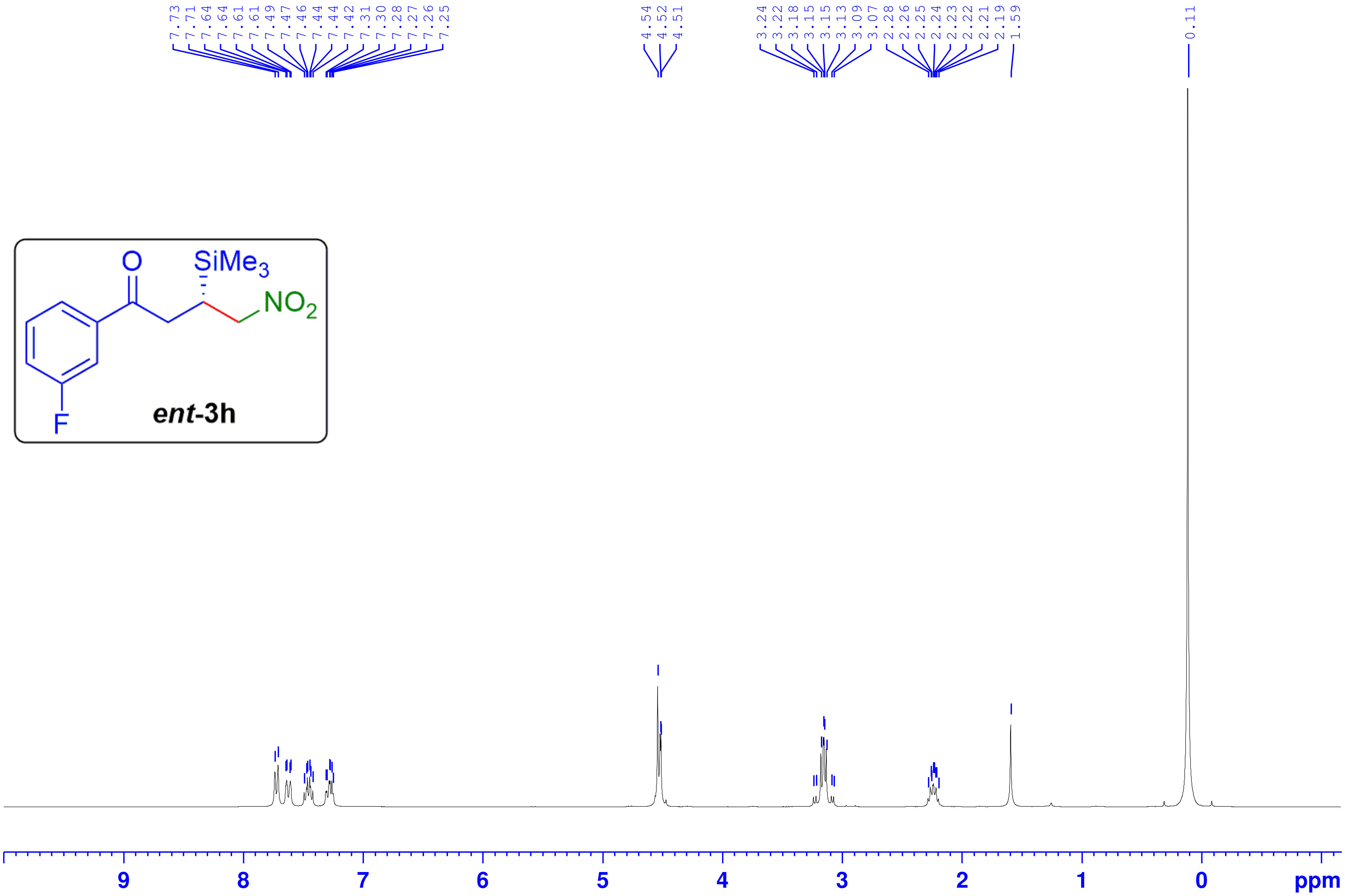
7.73
7.71
7.64
7.64
7.61
7.61
7.49
7.47
7.46
7.44
7.44
7.42
7.31
7.30
7.28
7.27
7.26
7.25
4.54
4.52
4.51
3.24
3.22
3.18
3.15
3.15
3.13
3.09
3.07
2.28
2.26
2.25
2.24
2.23
2.22
2.21
2.19
1.59
0.11
O
SiMe3
NO2
F
ent-3h
9
8
7
6
5
4
3
2
1
0
ppm

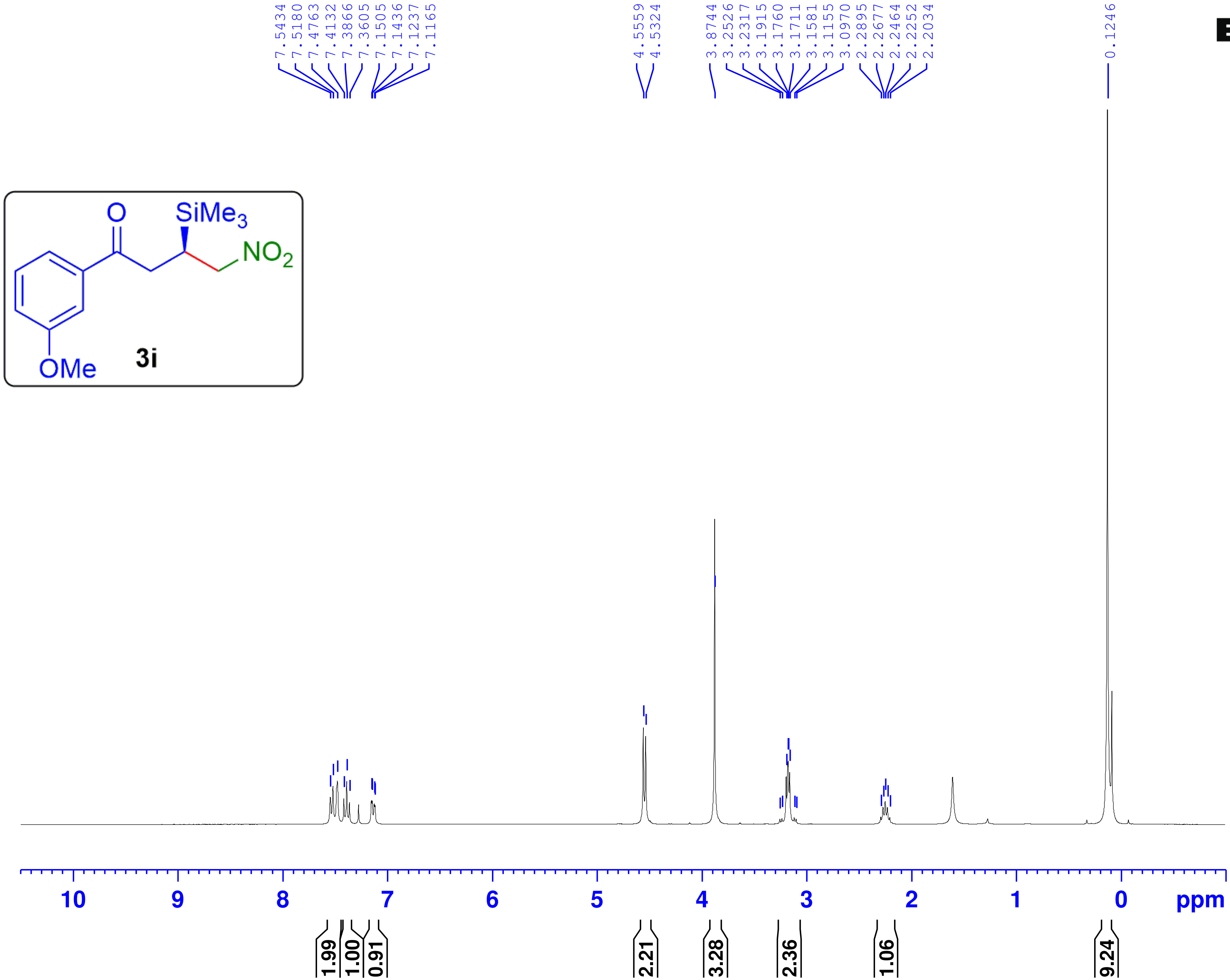


```
Current Data Parameters
NAME     akd 399 and akd 400 date12042021
EXPNO                  10
PROCNO                  1

F2 - Acquisition Parameters
Date_            20210412
Time                14.45
INSTRUM             spect
PROBHD   5 mm PABBO BB-
PULPROG                zg
TD                  32768
SOLVENT             CDCl3
NS                     16
DS                      0
SWH              4504.504 Hz
FIDRES           0.137467 Hz
AQ              3.6372480 sec
RG                   80.6
DW                111.000 usec
DE                   6.50 usec
TE                  299.7 K
D1             2.00000000 sec
TD0                     1

======== CHANNEL f1 ========
NUC1                   1H
P1                  13.50 usec
PL1                 -0.30 dB
PL1W          14.53428841 W
SFO1          300.1318008 MHz

F2 - Processing parameters
SI                  16384
SF            300.1300027 MHz
WDW                    EM
SSB      0
LB                   1.00 Hz
GB       0
PC                   1.00
```

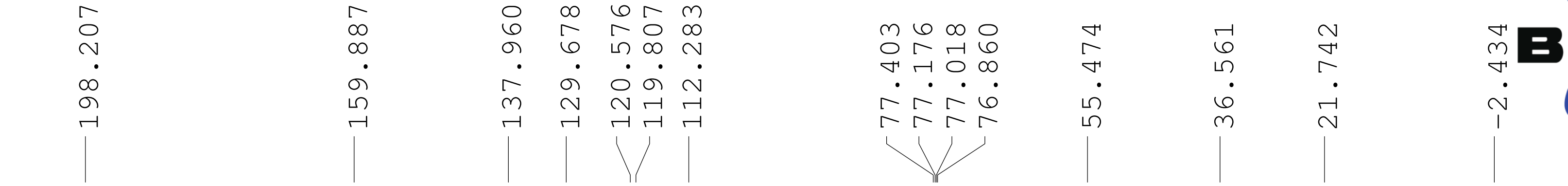

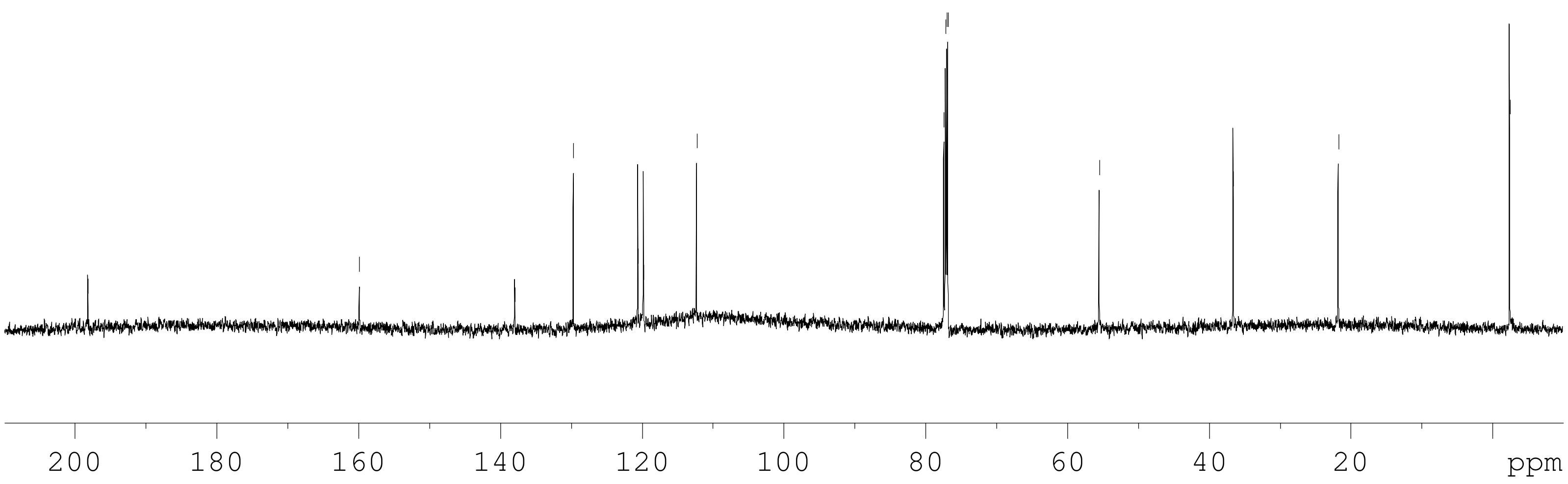

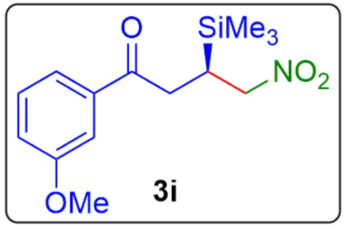

3i

BRUKER

```
Current Data Parameters
NAME      barc-akhil-13C-29Sep20
EXPNO                  7
PROCNO                 1

F2 - Acquisition Parameters
Date_           20200929
Time               14.50 h
INSTRUM            spect
PROBHD   Z555801_0012 (
PULPROG             zgdc
TD                 16384
SOLVENT            CDCl3
NS                   164
DS                     4
SWH            44247.789 Hz
FIDRES          5.401341 Hz
AQ             0.1851392 sec
RG                  71.8
DW                11.300 usec
DE                  6.50 usec
TE                 298.0 K
D1            3.00000000 sec
D11           0.03000000 sec
TD0                    1
SFO1         201.1878208 MHz
NUC1                 13C
P1                 11.00 usec
PLW1        312.79998779 W
SFO2         800.0332001 MHz
NUC2                  1H
CPDPRG[2         waltz16
PCPD2              60.00 usec
PLW2         13.00000000 W
PLW12         0.29249999 W

F2 - Processing parameters
SI                 16384
SF           201.1677040 MHz
WDW                   EM
SSB      0
LB                  5.00 Hz
GB       0
PC                  1.40
```

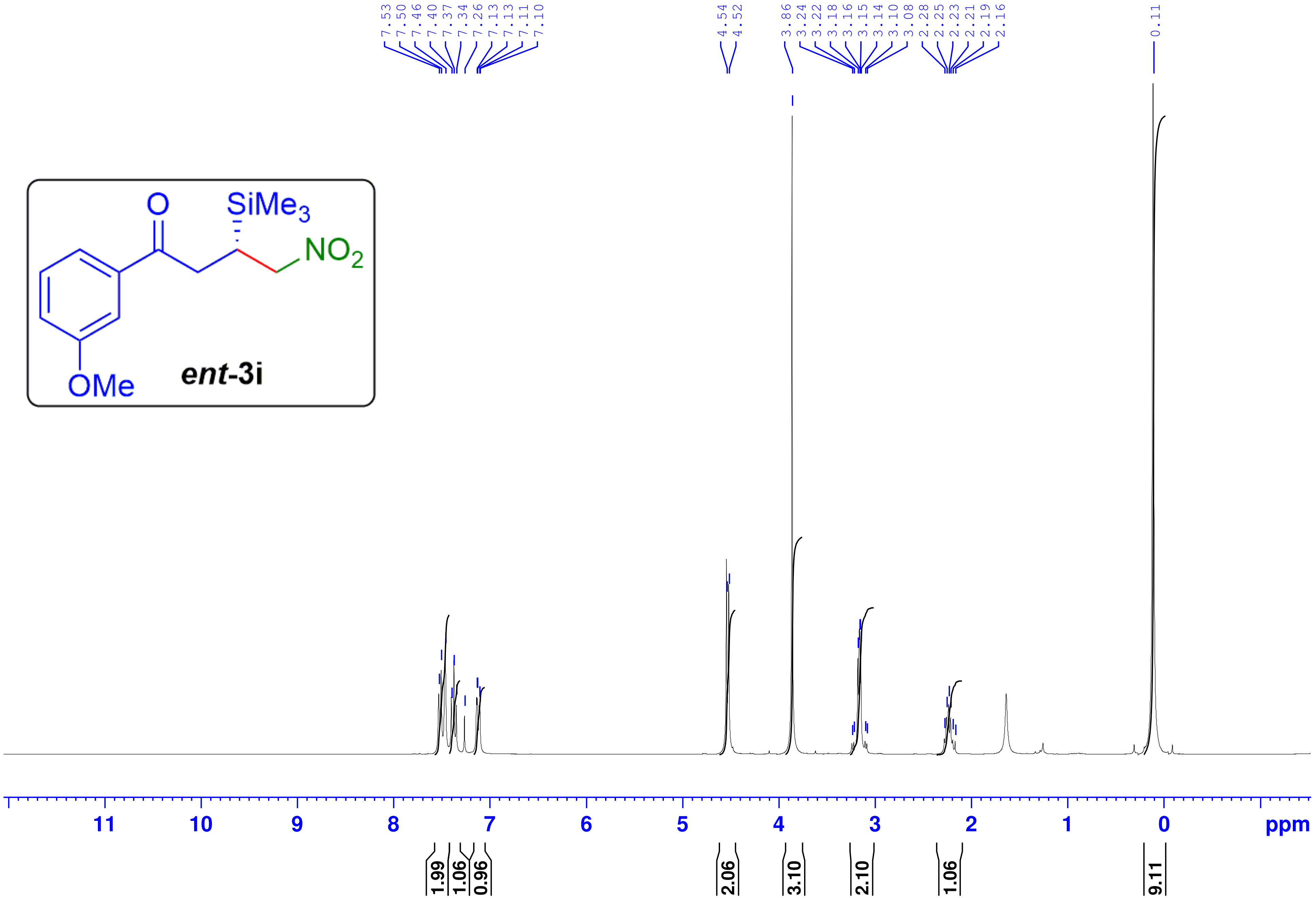
7.53
7.50
7.46
7.40
7.37
7.34
7.26
7.13
7.13
7.11
7.10
4.54
4.52
3.86
3.24
3.22
3.18
3.16
3.15
3.14
3.10
3.08
2.28
2.25
2.23
2.21
2.19
2.16
0.11
O
SiMe3
NO2
OMe
ent-3i
11
10
9
8
7
6
5
4
3
2
1
0
ppm
1.99
1.06
0.96
2.06
3.10
2.10
1.06
9.11

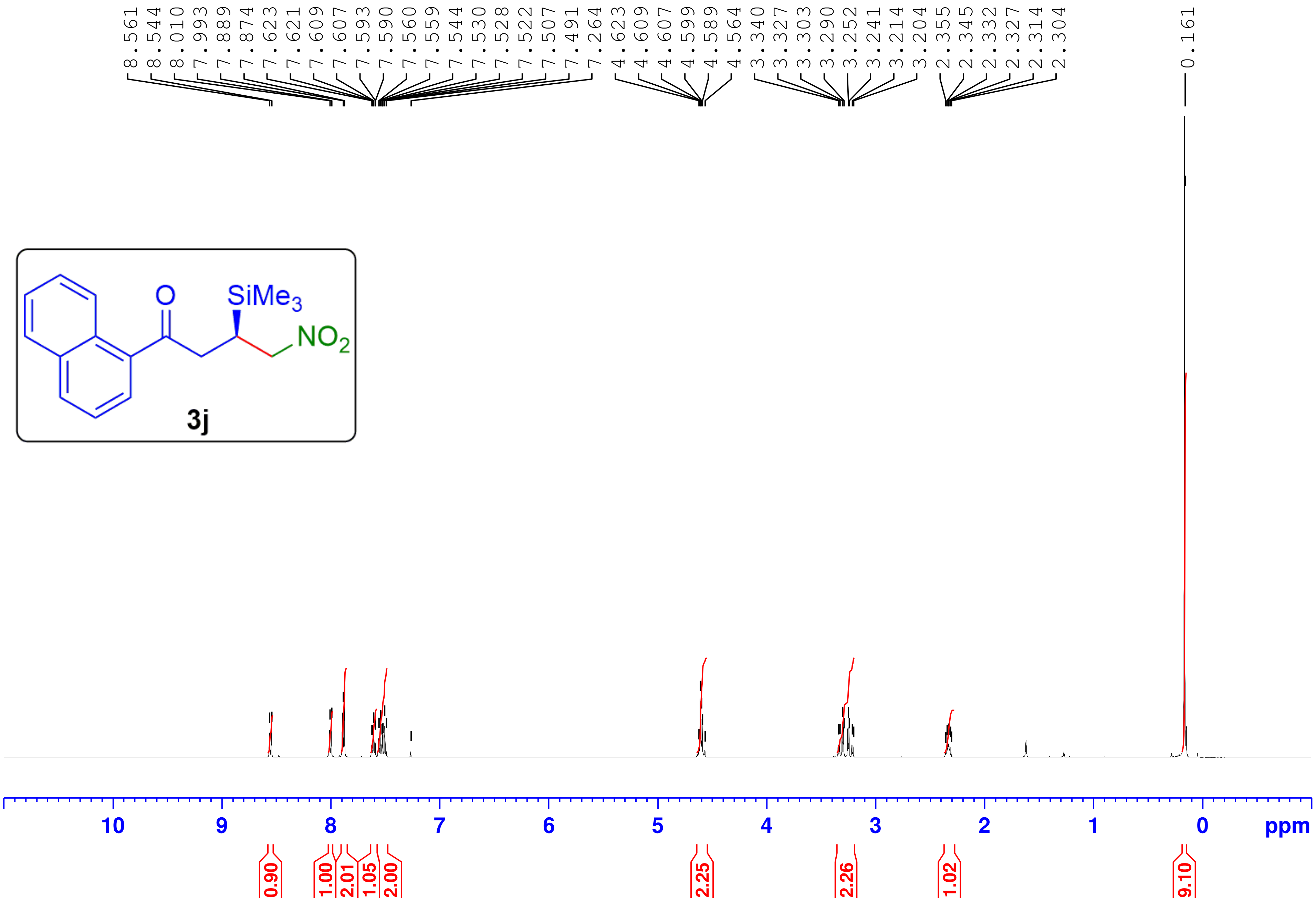
8.561
8.544
8.010
7.993
7.889
7.874
7.623
7.621
7.609
7.607
7.593
7.590
7.560
7.559
7.544
7.530
7.528
7.522
7.507
7.491
7.264
4.623
4.609
4.607
4.599
4.589
4.564
3.340
3.327
3.303
3.290
3.252
3.241
3.214
3.204
2.355
2.345
2.332
2.327
2.314
2.304
0.161
O
SiMe3
NO2
3j
10
9
8
7
6
5
4
3
2
1
0
ppm
0.90
1.00
2.01
1.05
2.00
2.25
2.26
1.02
9.10

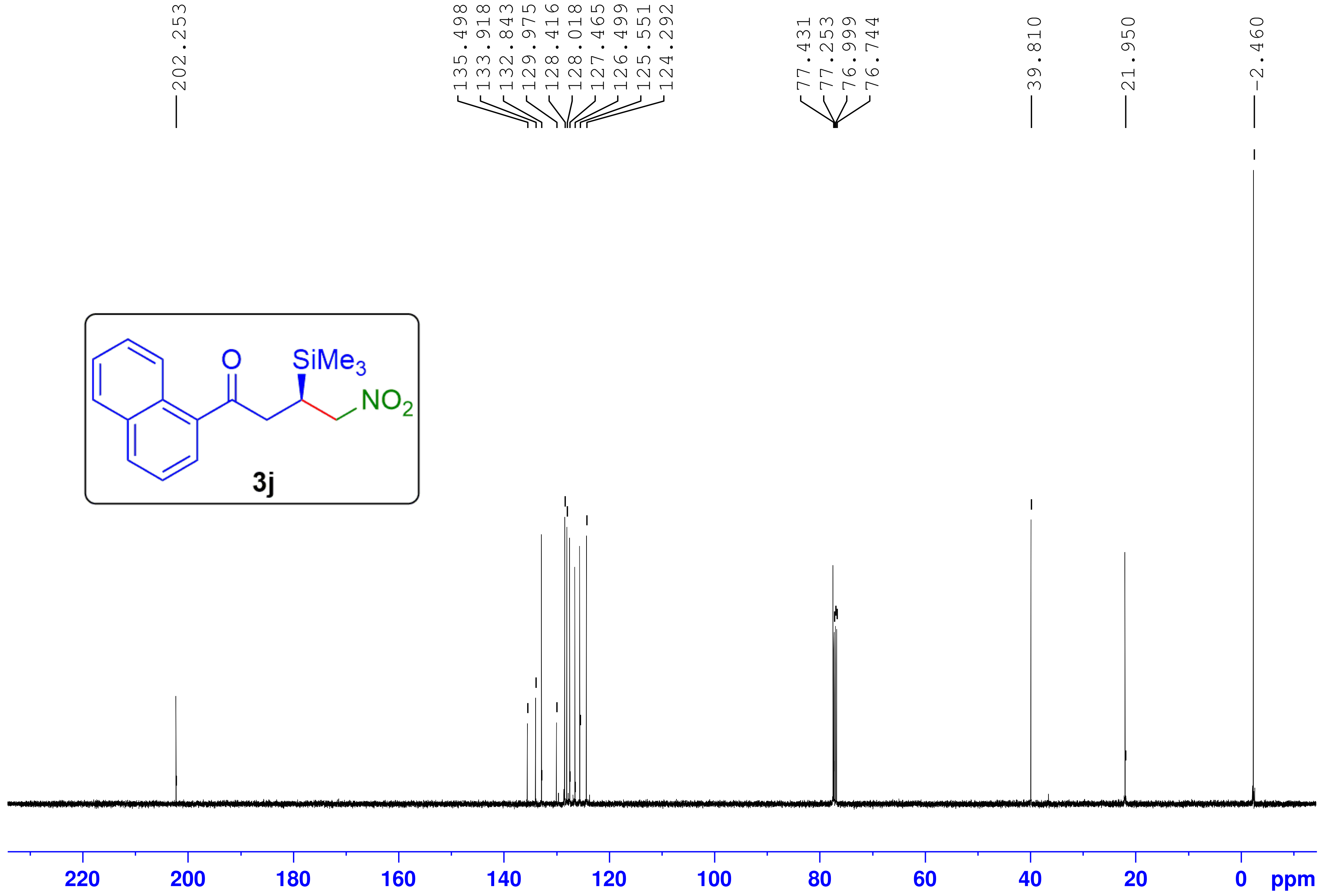
202.253
135.498
133.918
132.843
129.975
128.416
128.018
127.465
126.499
125.551
124.292
77.431
77.253
76.999
76.744
39.810
21.950
-2.460
O
SiMe3
NO2
3j
220
200
180
160
140
120
100
80
60
40
20
0
ppm

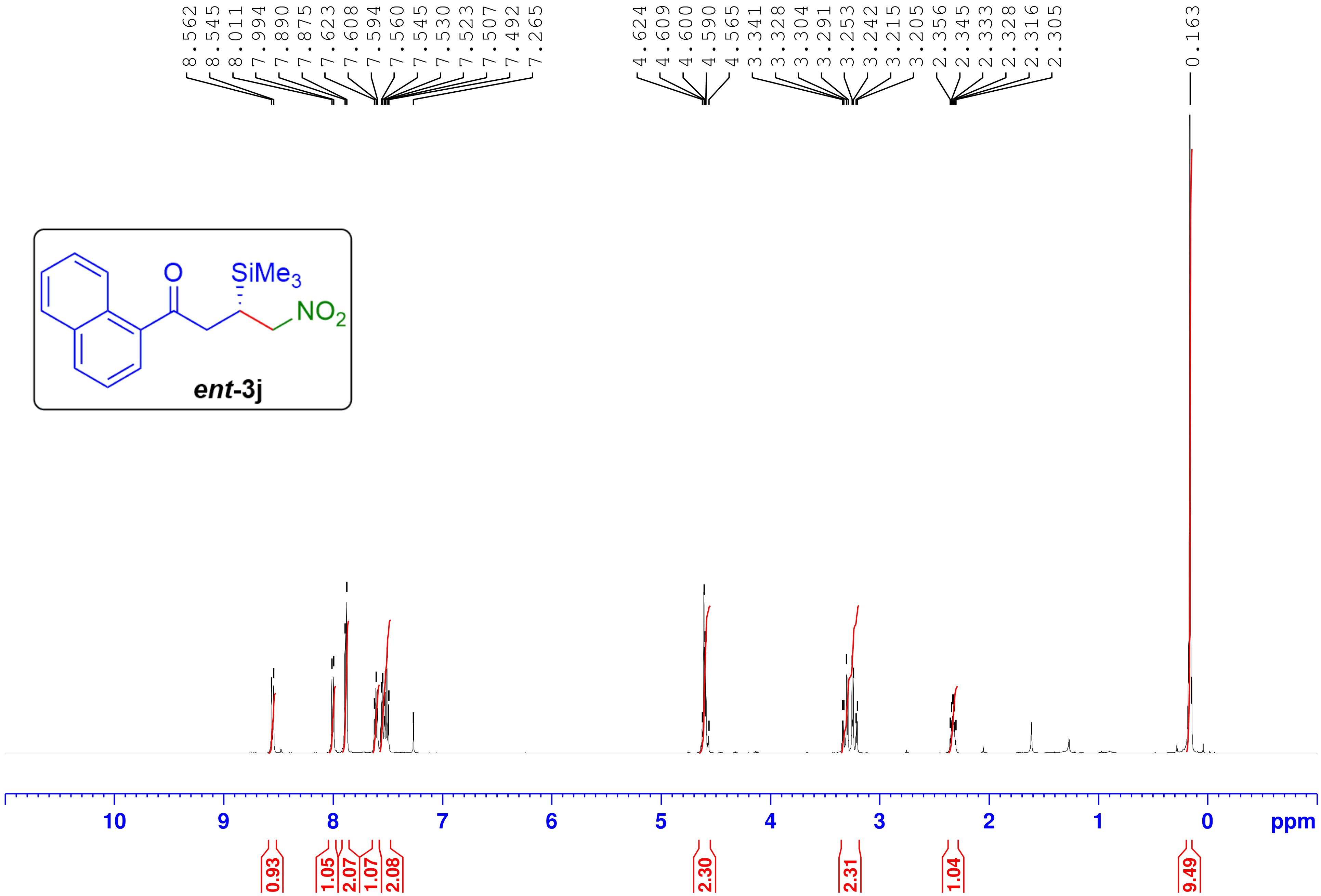

8.562
8.545
8.011
7.994
7.890
7.875
7.623
7.608
7.594
7.560
7.545
7.530
7.523
7.507
7.492
7.265
4.624
4.609
4.600
4.590
4.565
3.341
3.328
3.304
3.291
3.253
3.242
3.215
3.205
2.356
2.345
2.333
2.328
2.316
2.305
0.163
O
SiMe3
NO2
ent-3j
10
9
8
7
6
5
4
3
2
1
0
ppm
0.93
1.05
2.07
1.07
2.08
2.30
2.31
1.04
9.49

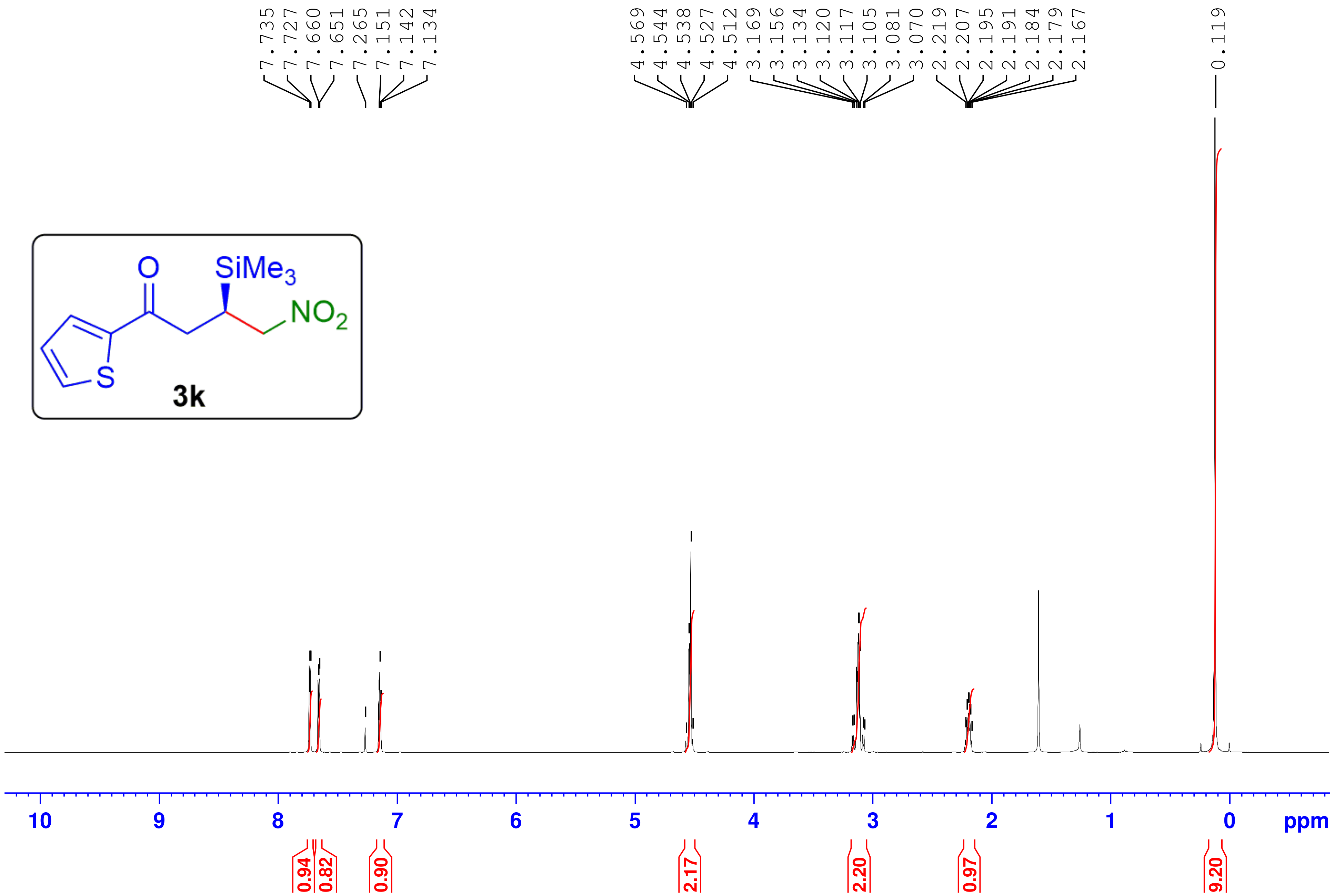
7.735
7.727
7.660
7.651
7.265
7.151
7.142
7.134
4.569
4.544
4.538
4.527
4.512
3.169
3.156
3.134
3.120
3.117
3.105
3.081
3.070
2.219
2.207
2.195
2.191
2.184
2.179
2.167
0.119
O
SiMe3
NO2
S
3k
10
9
8
7
6
5
4
3
2
1
0
ppm
0.94
0.82
0.90
2.17
2.20
0.97
9.20

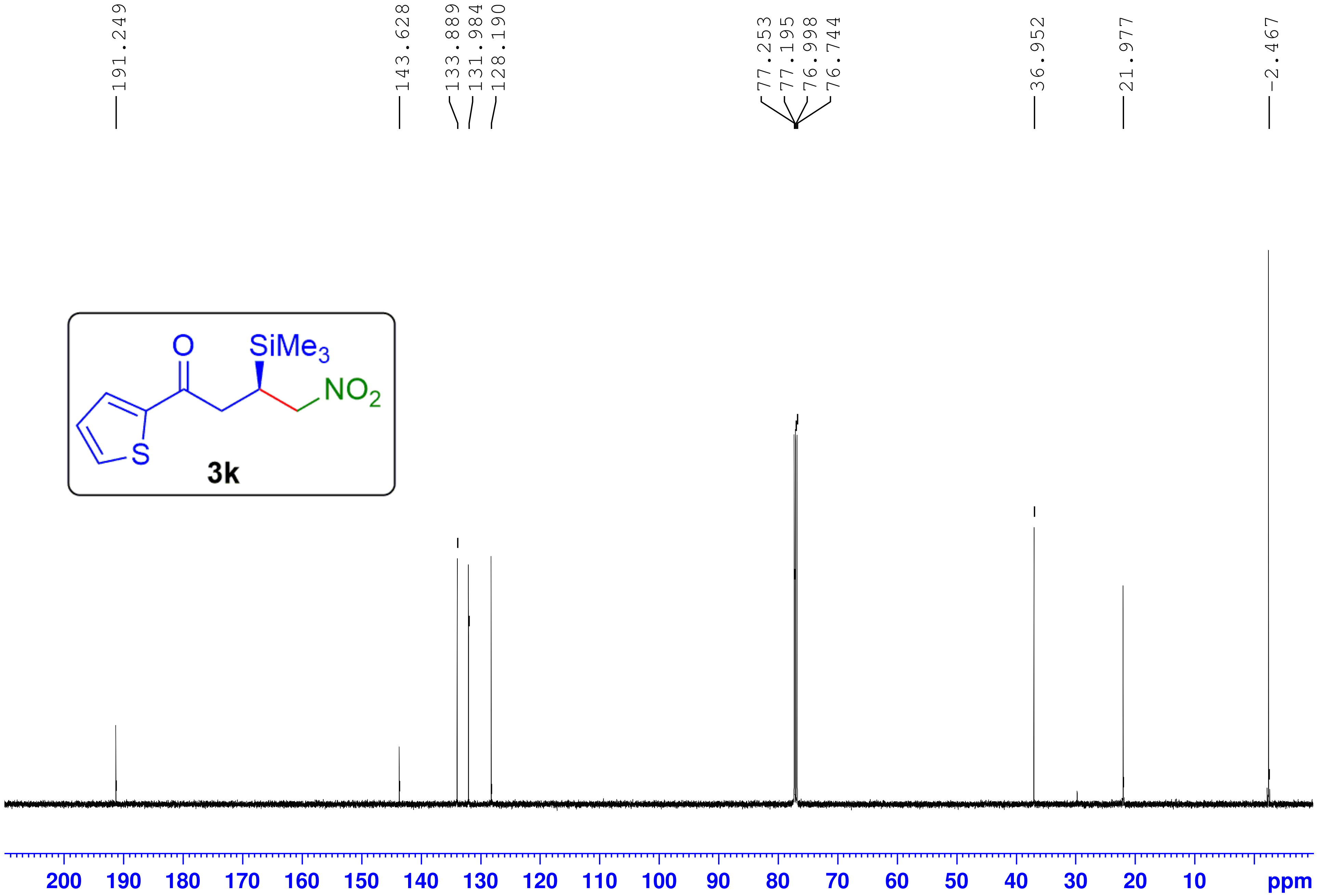
191.249
143.628
133.889
131.984
128.190
77.253
77.195
76.998
76.744
36.952
21.977
-2.467
O
SiMe3
NO2
S
3k
200
190
180
170
160
150
140
130
120
110
100
90
80
70
60
50
40
30
20
10
ppm

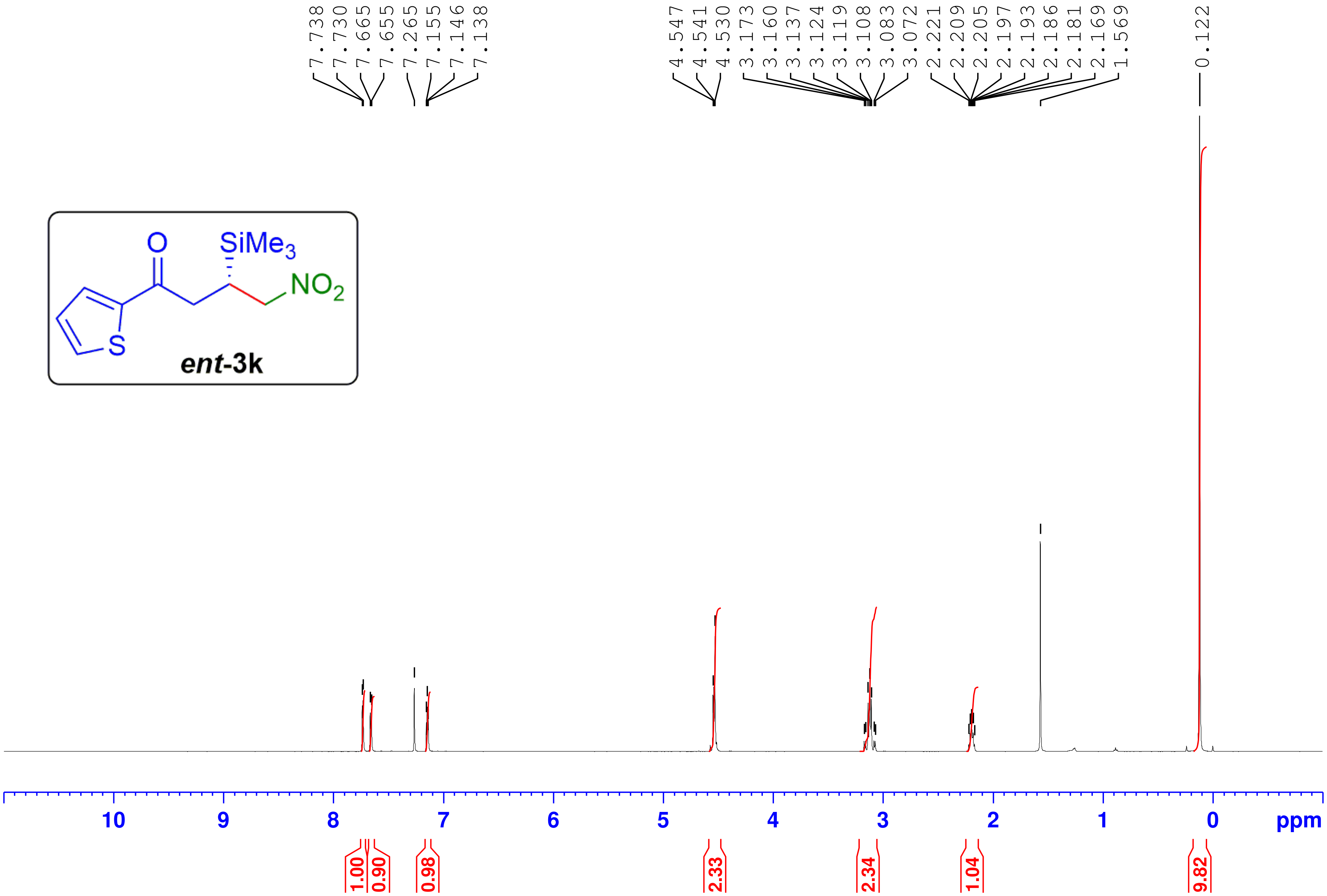
7.738
7.730
7.665
7.655
7.265
7.155
7.146
7.138
4.547
4.541
4.530
3.173
3.160
3.137
3.124
3.119
3.108
3.083
3.072
2.221
2.209
2.205
2.197
2.193
2.186
2.181
2.169
1.569
0.122
O
SiMe3
NO2
S
ent-3k
10
9
8
7
6
5
4
3
2
1
0
ppm
1.00
0.90
0.98
2.33
2.34
1.04
9.82

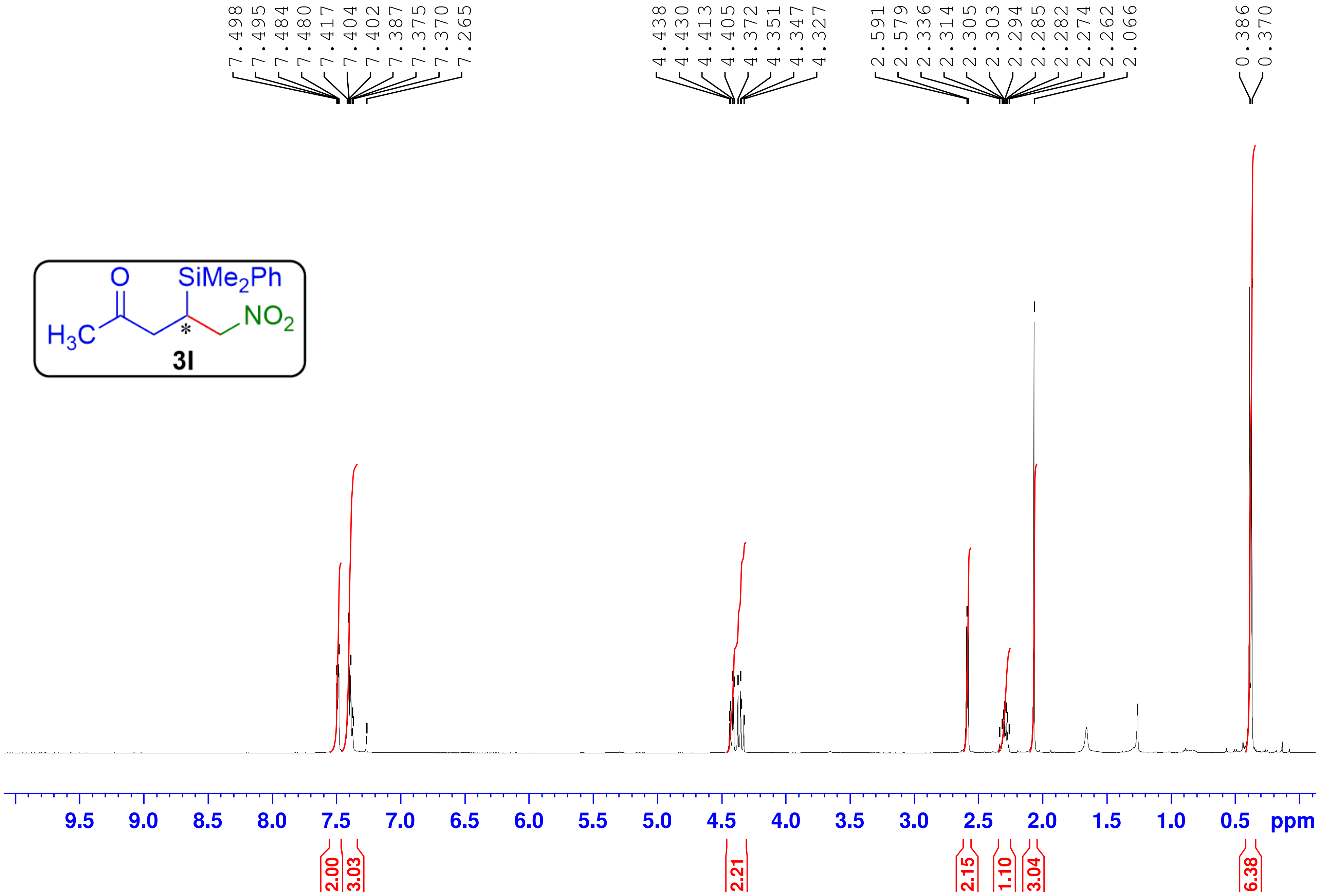

7.498
7.495
7.484
7.480
7.417
7.404
7.402
7.387
7.375
7.370
7.265
4.438
4.430
4.413
4.405
4.372
4.351
4.347
4.327
2.591
2.579
2.336
2.314
2.305
2.303
2.294
2.285
2.282
2.274
2.262
2.066
0.386
0.370
O
SiMe2Ph
H3C
NO2
3l
9.5
9.0
8.5
8.0
7.5
7.0
6.5
6.0
5.5
5.0
4.5
4.0
3.5
3.0
2.5
2.0
1.5
1.0
0.5
ppm
2.00
3.03
2.21
2.15
1.10
3.04
6.38

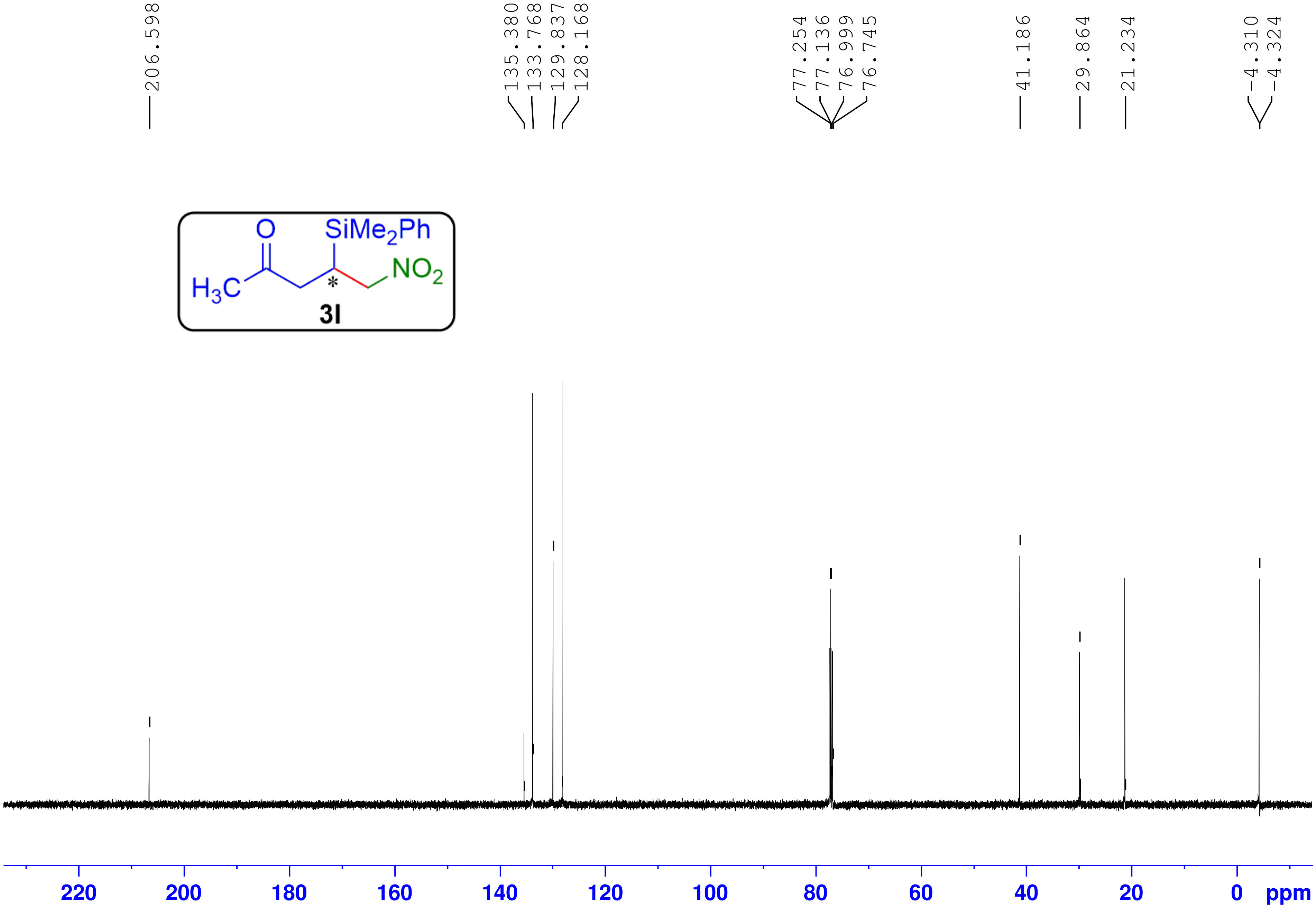
206.598
135.380
133.768
129.837
128.168
77.254
77.136
76.999
76.745
41.186
29.864
21.234
-4.310
-4.324
O
SiMe2Ph
NO2
H3C
3l
220
200
180
160
140
120
100
80
60
40
20
0
ppm

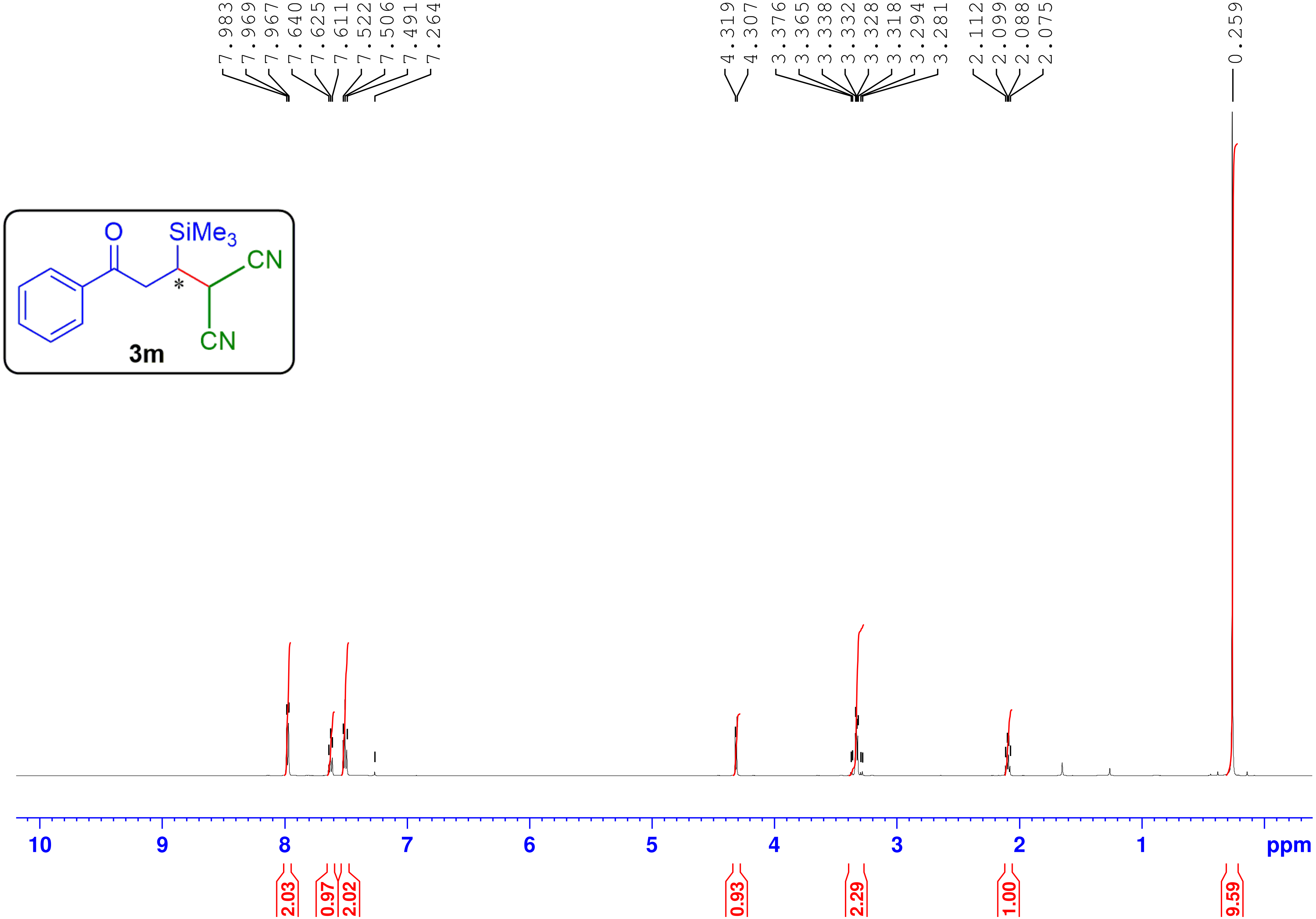
7.983
7.969
7.967
7.640
7.625
7.611
7.522
7.506
7.491
7.264
4.319
4.307
3.376
3.365
3.338
3.332
3.328
3.318
3.294
3.281
2.112
2.099
2.088
2.075
0.259
O
SiMe3
CN
CN
*
3m
10
9
8
7
6
5
4
3
2
1
ppm
2.03
0.97
2.02
0.93
2.29
1.00
9.59

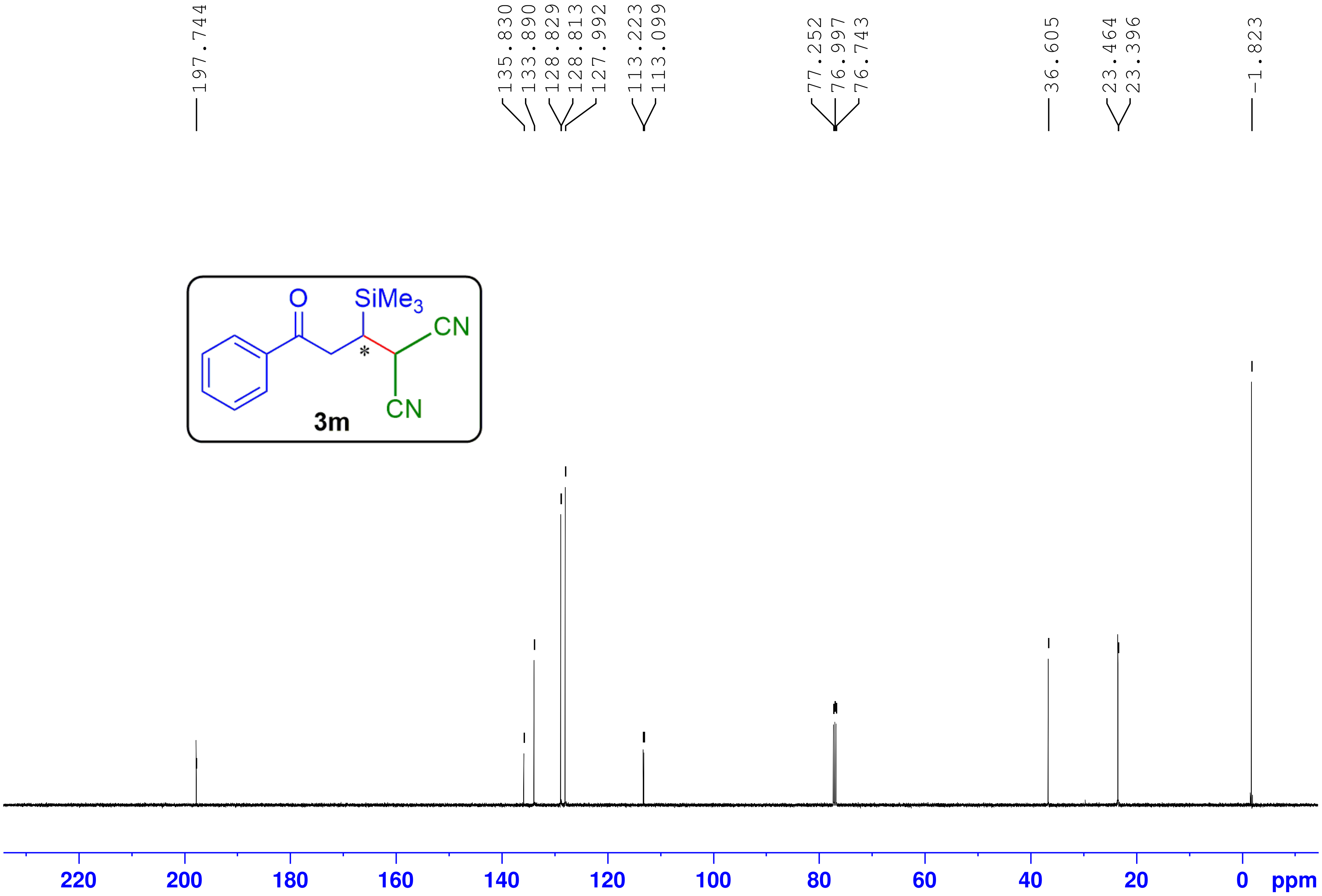
197.744
135.830
133.890
128.829
128.813
127.992
113.223
113.099
77.252
76.997
76.743
36.605
23.464
23.396
-1.823
O
SiMe3
CN
CN
*
3m
220
200
180
160
140
120
100
80
60
40
20
0
ppm

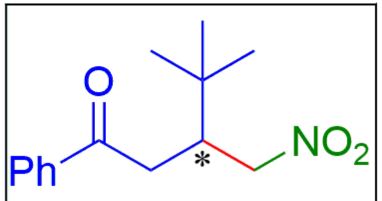

Chiral **4**

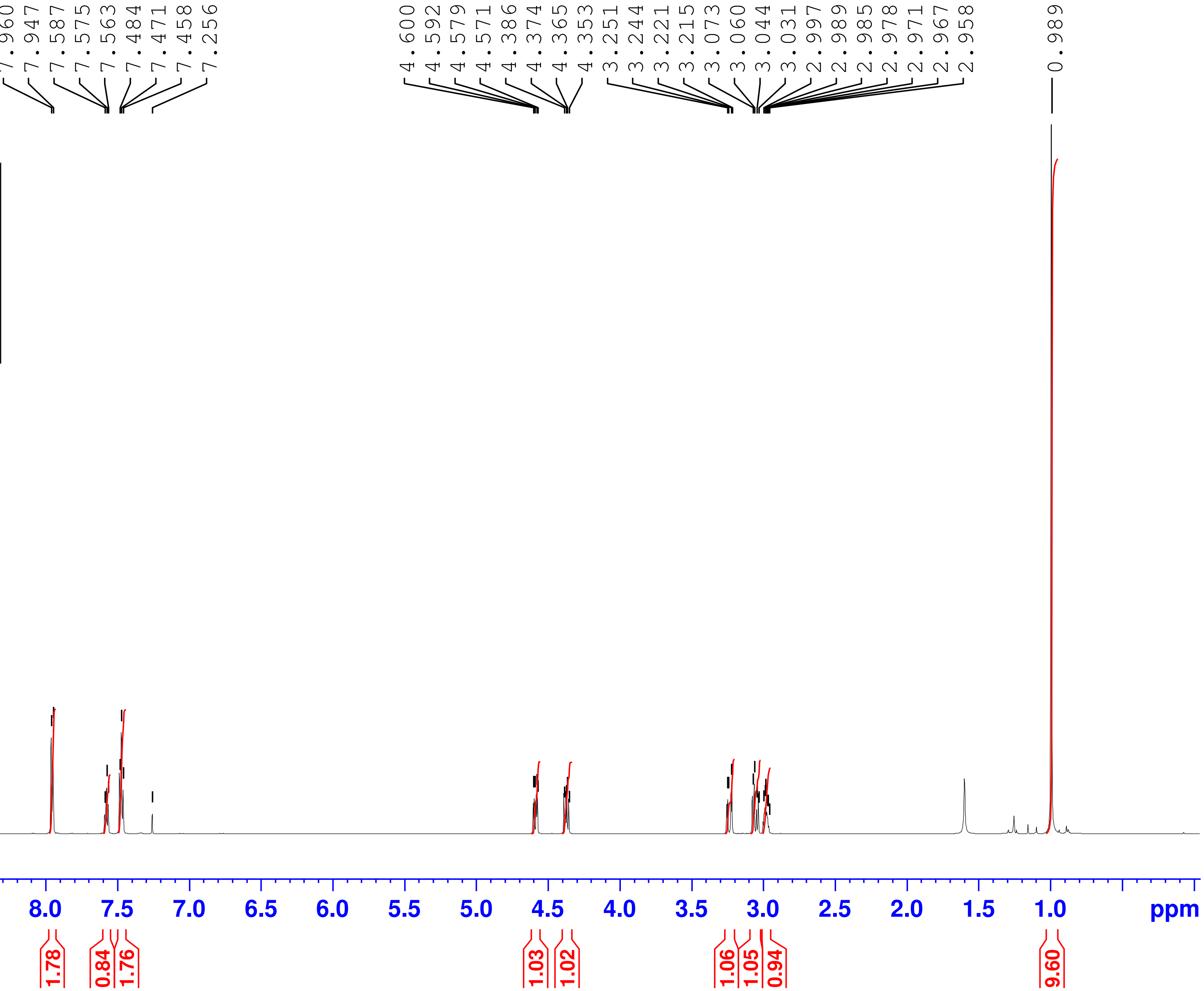
7.960
7.947
7.587
7.575
7.563
7.484
7.471
7.458
7.256
4.600
4.592
4.579
4.571
4.386
4.374
4.365
4.353
3.251
3.244
3.221
3.215
3.073
3.060
3.044
3.031
2.997
2.989
2.985
2.978
2.971
2.967
2.958
0.989
10.0
9.5
9.0
8.5
8.0
7.5
7.0
6.5
6.0
5.5
5.0
4.5
4.0
3.5
3.0
2.5
2.0
1.5
1.0
ppm
1.78
0.84
1.76
1.03
1.02
1.06
1.05
0.94
9.60

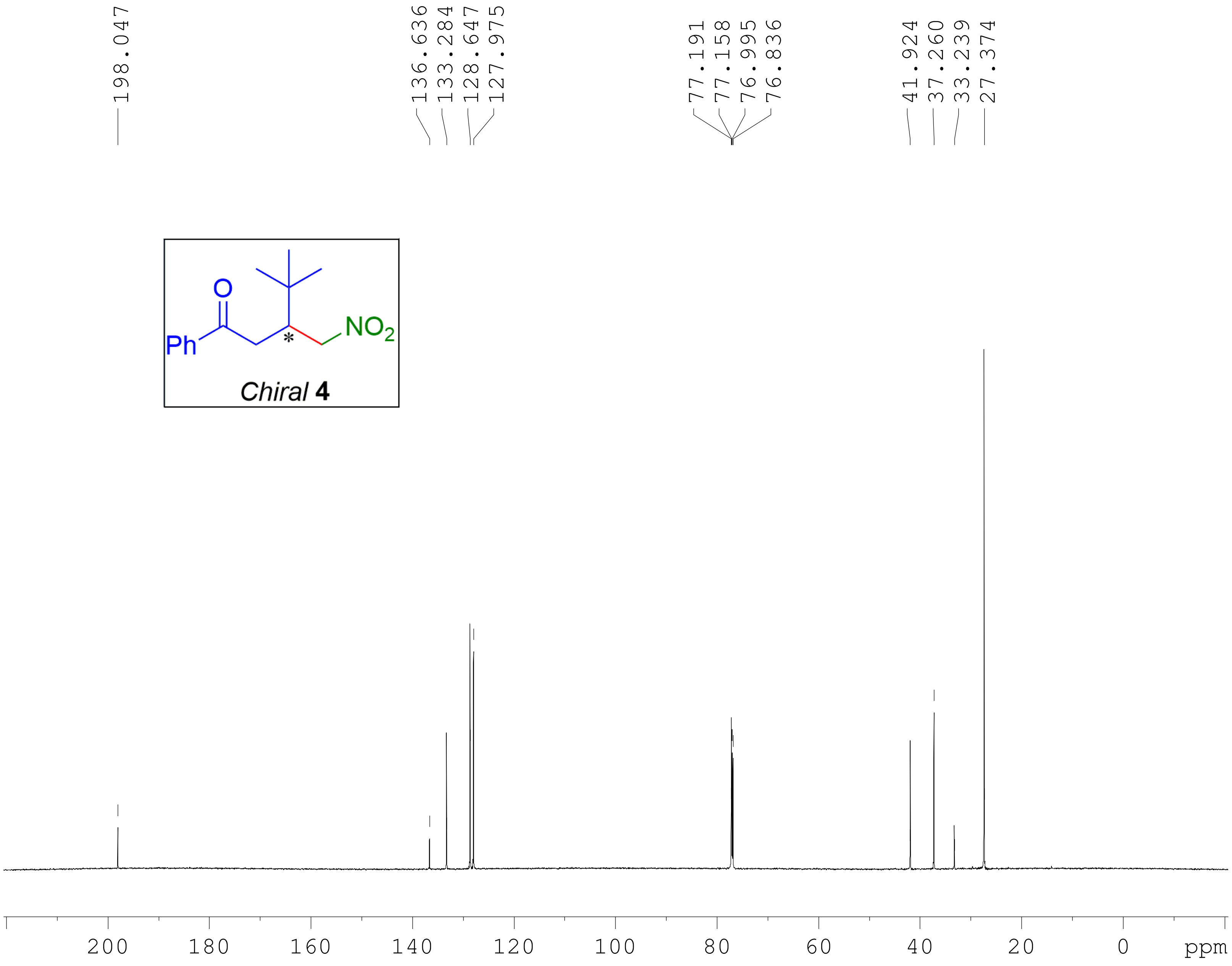

BRUKER

```
Current Data Parameters
NAME     barc-AKD412-13C-23Apr21
EXPNO                  1
PROCNO                 1

F2 - Acquisition Parameters
Date_           20210423
Time               16.24 h
INSTRUM            spect
PROBHD   Z44909_0011 (C
PULPROG             zgdc
TD                 16384
SOLVENT            CDCl3
NS                   537
DS                     4
SWH            48543.688 Hz
FIDRES          5.925743 Hz
AQ             0.1687552 sec
RG                  71.8
DW                10.300 usec
DE                 18.00 usec
TE                 298.0 K
D1            3.00000000 sec
D11           0.03000000 sec
TD0                    1
SFO1         201.1878208 MHz
NUC1                 13C
P1                 15.00 usec
PLW1        141.03999329 W
SFO2         800.0332001 MHz
NUC2                  1H
CPDPRG[2          waltz16
PCPD2              60.00 usec
PLW2         10.18999958 W
PLW12         0.16679481 W

F2 - Processing parameters
SI                 16384
SF           201.1677144 MHz
WDW                   EM
SSB      0
LB                  5.00 Hz
GB       0
PC                  1.40
```

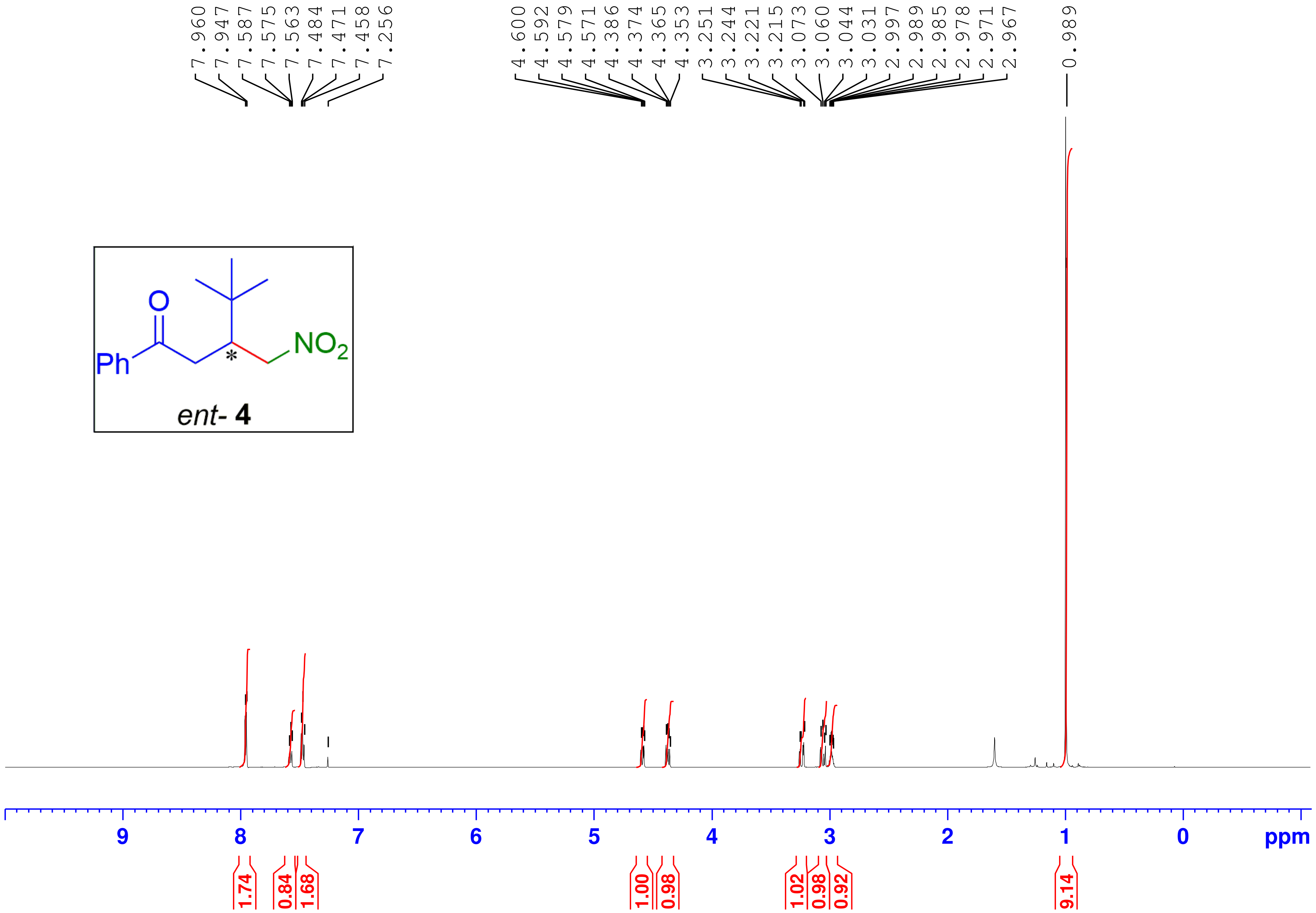
7.960
7.947
7.587
7.575
7.563
7.484
7.471
7.458
7.256
4.600
4.592
4.579
4.571
4.386
4.374
4.365
4.353
3.251
3.244
3.221
3.215
3.073
3.060
3.044
3.031
2.997
2.989
2.985
2.978
2.971
2.967
0.989
O
Ph
NO2
*
ent- 4
9
8
7
6
5
4
3
2
1
0
ppm
1.74
0.84
1.68
1.00
0.98
1.02
0.98
0.92
9.14

Sample Information

**Name**	BARC-AKD-359	**Data File Path**	D:\MassHunter\Data\JULY-21\BARC-AKD-359.d
**Sample ID**		**Acq. Time (Local)**	07-07-2021 4.13.44 PM (UTC+05:30)
**Instrument**	LCMS QTOF	**Method Path (Acq)**	D:\MassHunter\Methods\6545XT checkout\Methods\TRAINING\MS SCAN_AB_POS_100-1500_3500-300-120.m
**MS Type**	QTOF	**Version (Acq SW)**	6200 series TOF/6500 series Q-TOF B.09.00 (B9044.0)
**Inj. Vol. (ul)**	3	**IRM Status**	Success
**Position**	P2-A11	**Method Path (DA)**	D:\MassHunter\Report Templates\REPORT METHOD\HRMS.m
**Plate Pos.**		**Target Source Path**	
**Operator**		**Result Summary**	1 qualified (1 targets)

Sample Spectra

HRMS spectra of 3k

+ Scan (rt: 0.253-0.553 min) **Peak 1 from + TIC Scan**

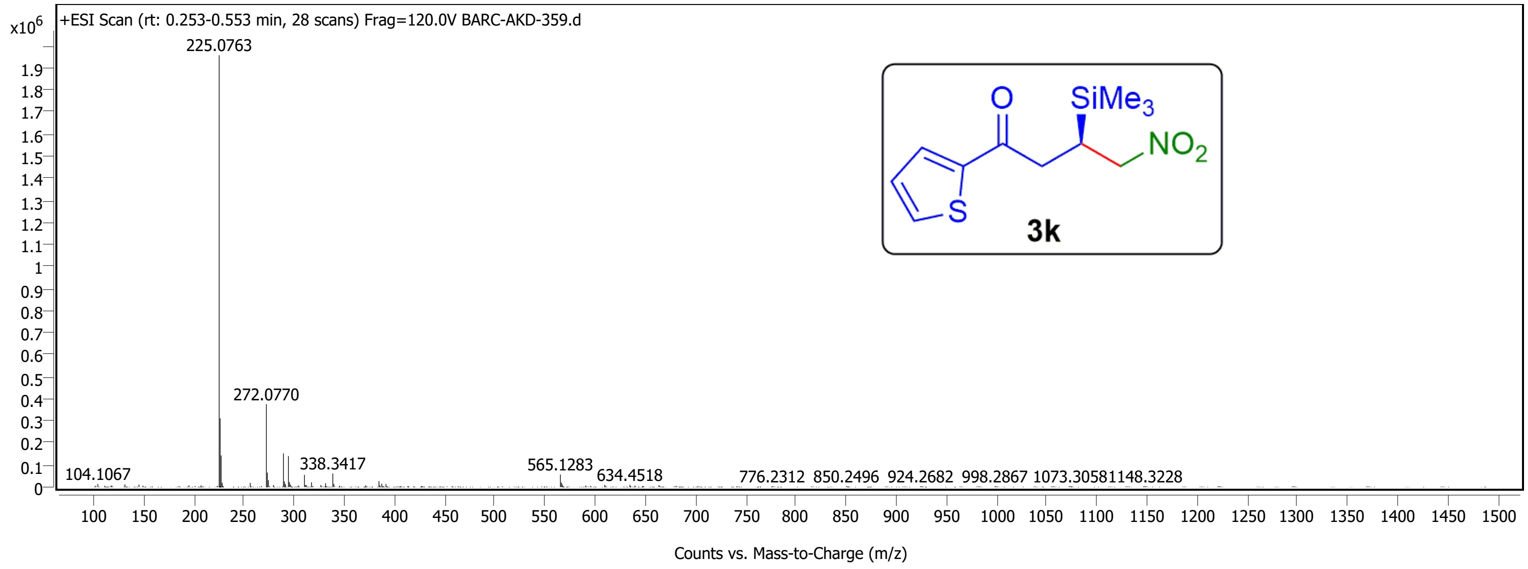

Compound Details

Cpd. 1: C11 H17 N O3 S Si

Formula	m/z	Observed M/Z	Difference Da	Difference PPM	Score
C11 H17 N O3 S Si	272.0770	272.077007144853	-0.101273064672114	-0.373605062201966	99.27

Compound Spectra (Zoomed)

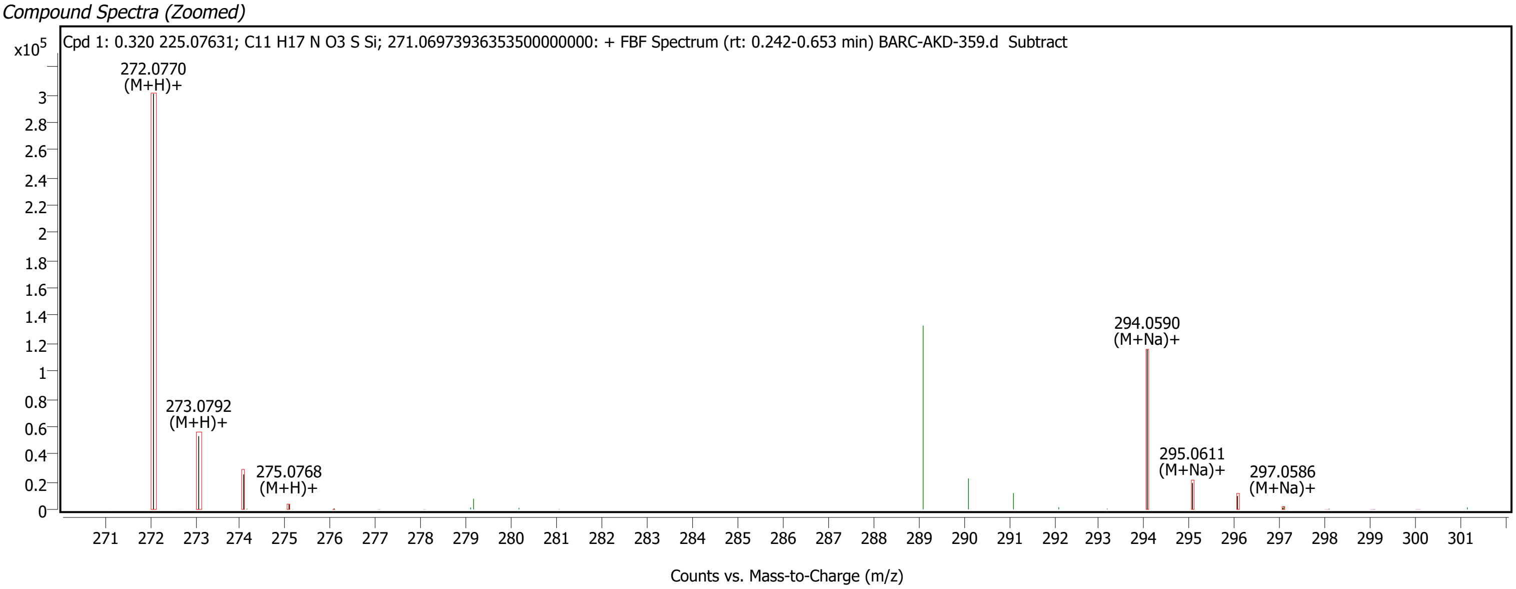

MassHunter Qual 10.0
(End of Report)

checkCIF/PLATON report

Structure factors have been supplied for datablock(s) barc-akd-294_mo

THIS REPORT IS FOR GUIDANCE ONLY. IF USED AS PART OF A REVIEW PROCEDURE FOR PUBLICATION, IT SHOULD NOT REPLACE THE EXPERTISE OF AN EXPERIENCED CRYSTALLOGRAPHIC REFEREE.

No syntax errors found. CIF dictionary Interpreting this report

Datablock: *ent*-3k

Bond precision: C-C = 0.0046 A Wavelength=0.71073

Cell:	a=6.4038(3)	b=10.0958(5)	c=21.3672(9)
	alpha=90	beta=90	gamma=90
Temperature:	293 K		

	Calculated	Reported
Volume	1381.42(11)	1381.42(11)
Space group	P 21 21 21	P 21 21 21
Hall group	P 2ac 2ab	P 2ac 2ab
Moiety formula	C11 H17 N O3 S Si	C11 H17 N O3 S Si
Sum formula	C11 H17 N O3 S Si	C11 H17 N O3 S Si
Mr	271.41	271.40
Dx,g cm-3	1.305	1.305
Z	4	4
Mu (mm-1)	0.317	0.317
F000	576.0	576.0
F000’	577.05	
h,k,lmax	7,12,25	7,12,25
Nref	2436[1438]	2435
Tmin,Tmax		0.435,1.000
Tmin’		

Correction method= # Reported T Limits: Tmin=0.435 Tmax=1.000
AbsCorr = MULTI-SCAN

Data completeness= 1.69/1.00 Theta(max)= 24.996

R(reflections)= 0.0377(2243) wR2(reflections)= 0.0792(2435)

S = 1.051 Npar= 157

The following ALERTS were generated. Each ALERT has the format
test-name_ALERT_alert-type_alert-level.
Click on the hyperlinks for more details of the test.

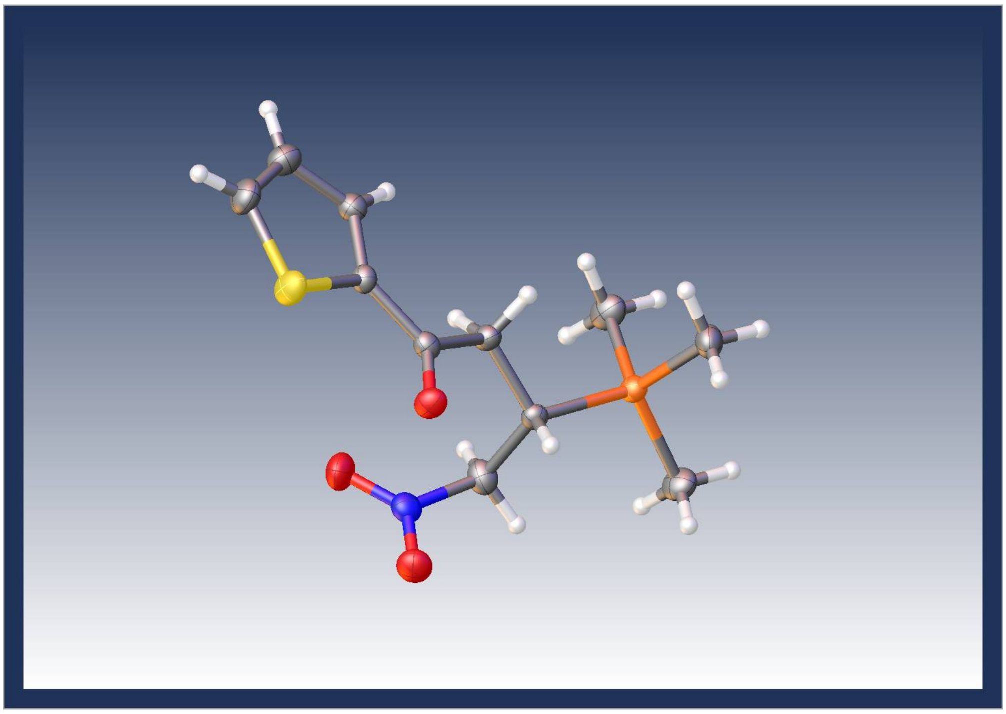

Table 1 Crystal data and structure refinement for *ent*-3k

Identification code	***ent*-3k**
Empirical formula	$C_{11}H_{17}NO_3SSi$
Formula weight	271.40
Temperature/K	293(2)
Crystal system	orthorhombic
Space group	$P2_12_12_1$
a/Å	6.4038(3)
b/Å	10.0958(5)
c/Å	21.3672(9)
α/°	90
β/°	90
γ/°	90
Volume/Å^3	1381.42(11)
Z	4
ρ_{calc}g/cm^3	1.305
μ/mm^{-1}	0.317
F(000)	576.0
Crystal size/mm^3	? × ? × ?
Radiation	MoKα (λ = 0.71073)
2Θ range for data collection/°	4.462 to 49.992
Index ranges	-7 ≤ h ≤ 7, -12 ≤ k ≤ 12, -25 ≤ l ≤ 25
Reflections collected	10751
Independent reflections	2435 [R_{int} = 0.0606, R_{sigma} = 0.0468]
Data/restraints/parameters	2435/0/157

Goodness-of-fit on F^2	1.051
Final R indexes [I>=2σ (I)]	R_1 = 0.0377, wR_2 = 0.0749
Final R indexes [all data]	R_1 = 0.0431, wR_2 = 0.0792
Largest diff. peak/hole / e $Å^{-3}$	0.22/-0.20
Flack parameter	-0.12(7)

Table 2 Fractional Atomic Coordinates (×10^4) and Equivalent Isotropic Displacement Parameters (Å^2×10^3) for BARC-AKD-294_Mo. U_{eq} is defined as 1/3 of of the trace of the orthogonalised U_{IJ} tensor.

Atom	*x*	*y*	*z*	U(eq)
S(1)	-3060.1(15)	-4157.8(10)	-4448.2(4)	32.0(3)
Si(1)	-3729.6(15)	-3540.9(9)	-1223.3(4)	20.6(2)
O(1)	-978(4)	-4141(2)	-3207.2(11)	27.1(6)
O(3)	1335(4)	-1726(2)	-2638.3(12)	33.8(6)
O(2)	-1492(4)	-984(3)	-3066.3(12)	42.0(7)
N(1)	-526(5)	-1468(3)	-2628.2(14)	26.5(7)
C(4)	-4187(5)	-3922(3)	-3723.7(15)	19.6(7)
C(5)	-2838(5)	-3905(3)	-3165.9(15)	20.4(7)
C(7)	-2415(5)	-3224(3)	-2009.9(14)	18.7(7)
C(3)	-6296(5)	-3764(3)	-3775.3(15)	23.1(7)
C(2)	-7002(6)	-3832(4)	-4397.0(17)	30.9(9)
C(8)	-1727(6)	-1779(3)	-2042.6(15)	27.2(9)
C(11)	-2211(6)	-2747(4)	-584.7(16)	33.0(9)
C(6)	-3869(5)	-3621(3)	-2543.6(14)	21.7(7)
C(10)	-3867(6)	-5360(3)	-1096.4(17)	30.7(9)
C(9)	-6421(6)	-2847(4)	-1238.4(17)	34.2(9)
C(1)	-5422(6)	-4034(4)	-4806.1(17)	35(1)

Table 3 Anisotropic Displacement Parameters (Å^2×10^3) for BARC-AKD-294_Mo. The Anisotropic displacement factor exponent takes the form: -2π^2 [h^{2}a*^{2}U$_{11}$+2hka*b*U$_{12}$+…].

Atom	U_{11}	U_{22}	U_{33}	U_{23}	U_{13}	U_{12}
S(1)	28.6(5)	48.3(6)	19.0(4)	-2.0(4)	4.3(4)	2.0(5)
Si(1)	20.5(5)	23.7(5)	17.5(5)	-2.1(4)	0.8(4)	0.2(4)
O(1)	20.1(13)	35.6(13)	25.4(13)	-1.8(11)	2.5(10)	3.6(12)
O(3)	24.0(15)	40.3(15)	37.0(15)	2.9(12)	3.0(13)	2.2(13)
O(2)	44.1(17)	43.2(16)	38.8(15)	17.7(13)	-10.0(14)	-3.1(15)
N(1)	28.5(18)	20.6(15)	30.4(17)	1.6(14)	-0.2(14)	-2.1(15)
C(4)	22.6(18)	19.2(17)	17.0(16)	-1.0(14)	2.2(14)	1.5(14)
C(5)	20.5(18)	18.4(16)	22.4(18)	0.5(14)	3.3(15)	0.6(15)
C(7)	15.7(17)	20.5(17)	20.0(17)	-0.5(14)	0.9(14)	2.3(14)
C(3)	23.4(18)	22.4(17)	23.5(18)	-1.1(15)	1.3(16)	-0.3(15)
C(2)	27.1(19)	33(2)	32(2)	-0.4(17)	-4.2(18)	1.8(17)
C(8)	33(2)	23.5(18)	25.1(19)	-1.6(15)	6.2(18)	2.1(17)
C(11)	36(2)	41(2)	22.9(19)	-7.0(17)	-1.9(19)	-3.2(18)
C(6)	19.2(17)	26.7(17)	19.2(17)	-1.3(14)	0.4(14)	-0.8(17)
C(10)	38(2)	29.7(19)	24.8(19)	5.1(15)	-1.3(18)	-3.4(18)
C(9)	29(2)	47(2)	26(2)	-4.7(18)	3.2(19)	4.5(18)
C(1)	40(2)	43(2)	22.6(19)	0.9(18)	-3.8(18)	0(2)

Table 4 Bond Lengths for BARC-AKD-294_Mo.

Atom	Atom	Length/Å
S(1)	C(4)	1.725(3)
S(1)	C(1)	1.699(4)
Si(1)	C(7)	1.907(3)
Si(1)	C(11)	1.857(4)
Si(1)	C(10)	1.859(3)
Si(1)	C(9)	1.861(4)
O(1)	C(5)	1.218(4)
O(3)	N(1)	1.220(4)
O(2)	N(1)	1.223(4)
N(1)	C(8)	1.502(4)
C(4)	C(5)	1.472(4)
C(4)	C(3)	1.364(5)
C(5)	C(6)	1.512(4)
C(7)	C(8)	1.526(4)
C(7)	C(6)	1.526(4)
C(3)	C(2)	1.405(5)
C(2)	C(1)	1.353(5)

Table 5 Bond Angles for BARC-AKD-294_Mo.

Atom	Atom	Atom	Angle/˚	Atom	Atom	Atom	Angle/˚
C(1)	S(1)	C(4)	91.23(17)	C(3)	C(4)	C(5)	130.2(3)
C(11)	Si(1)	C(7)	110.12(16)	O(1)	C(5)	C(4)	120.9(3)
C(11)	Si(1)	C(10)	110.13(17)	O(1)	C(5)	C(6)	121.9(3)
C(11)	Si(1)	C(9)	109.58(18)	C(4)	C(5)	C(6)	117.2(3)
C(10)	Si(1)	C(7)	108.37(16)	C(8)	C(7)	Si(1)	109.2(2)
C(10)	Si(1)	C(9)	109.31(19)	C(8)	C(7)	C(6)	113.1(3)
C(9)	Si(1)	C(7)	109.31(16)	C(6)	C(7)	Si(1)	110.2(2)
O(3)	N(1)	O(2)	124.4(3)	C(4)	C(3)	C(2)	112.9(3)
O(3)	N(1)	C(8)	118.1(3)	C(1)	C(2)	C(3)	112.2(3)
O(2)	N(1)	C(8)	117.5(3)	N(1)	C(8)	C(7)	112.7(3)
C(5)	C(4)	S(1)	118.9(2)	C(5)	C(6)	C(7)	116.1(3)
C(3)	C(4)	S(1)	111.0(3)	C(2)	C(1)	S(1)	112.7(3)

Table 6 Torsion Angles for BARC-AKD-294_Mo.

A	B	C	D	Angle/˚	A	B	C	D	Angle/˚
S(1)	C(4)	C(5)	O(1)	-4.8(4)	C(4)	C(3)	C(2)	C(1)	-0.3(5)
S(1)	C(4)	C(5)	C(6)	176.7(2)	C(5)	C(4)	C(3)	C(2)	179.4(3)
S(1)	C(4)	C(3)	C(2)	-0.1(4)	C(3)	C(4)	C(5)	O(1)	175.8(3)
Si(1)	C(7)	C(8)	N(1)	175.6(2)	C(3)	C(4)	C(5)	C(6)	-2.7(5)
Si(1)	C(7)	C(6)	C(5)	-158.5(2)	C(3)	C(2)	C(1)	S(1)	0.5(4)
O(1)	C(5)	C(6)	C(7)	18.2(5)	C(8)	C(7)	C(6)	C(5)	79.0(4)
O(3)	N(1)	C(8)	C(7)	-82.7(4)	C(6)	C(7)	C(8)	N(1)	-61.4(4)
O(2)	N(1)	C(8)	C(7)	96.5(4)	C(1)	S(1)	C(4)	C(5)	-179.2(3)
C(4)	S(1)	C(1)	C(2)	-0.4(3)	C(1)	S(1)	C(4)	C(3)	0.3(3)
C(4)	C(5)	C(6)	C(7)	-163.3(3)					

Table 7 Hydrogen Atom Coordinates (Å×10^4) and Isotropic Displacement Parameters (Å^2×10^3) for BARC-AKD-294_Mo.

Atom	*x*	*y*	*z*	U(eq)
H(7)	-1163	-3781	-2034	22
H(3)	-7174	-3627	-3435	28
H(2)	-8395	-3749	-4514	37
H(8A)	-2952	-1215	-2023	33
H(8B)	-863	-1580	-1682	33
H(11A)	-793	-3054	-598	49
H(11B)	-2819	-2973	-188	49
H(11C)	-2236	-1803	-638	49
H(6A)	-4637	-4404	-2416	26
H(6B)	-4877	-2915	-2604	26
H(10A)	-4714	-5755	-1417	46
H(10B)	-4472	-5537	-694	46
H(10C)	-2486	-5728	-1113	46
H(9A)	-6357	-1915	-1325	51
H(9B)	-7074	-2986	-839	51
H(9C)	-7219	-3282	-1558	51
H(1)	-5607	-4098	-5237	42

Experimental

A suitable crystal was selected and and single crystal X-ray diffraction data of ***ent*-3k** was collected on a on a **dtrek-CrysAlisPro-abstract goniometer imported rigaku-d*trek images** diffractometer. The crystal was kept at 293 (2) K during data collection. Using Olex2 [1], the structure was solved with the ShelXT [2] structure solution program using Direct Methods and refined with the ShelXL [3] refinement package using Least Squares minimisation.

1. Dolomanov, O.V., Bourhis, L.J., Gildea, R.J, Howard, J.A.K. & Puschmann, H. (2009), J. Appl. Cryst. 42, 339-341.
2. Sheldrick, G.M. (2015). Acta Cryst. A71, 3-8.
3. Sheldrick, G.M. (2008). Acta Cryst. A64, 112-122.

Crystal structure determination of *ent*-3k

Crystal Data for $C_{11}H_{17}NO_3SSi$ (M =271.40 g/mol): orthorhombic, space group $P2_12_12_1$ (no. 19), a = 6.4038 (3) Å, b = 10.0958(5) Å, c = 21.3672(9) Å, V = 1381.42(11) Å^3, Z = 4, T = 293(2) K, μ(MoKα) = 0.317 mm^{-1}, $Dcalc$ = 1.305 g/cm^3, 10751 reflections measured (4.462° ≤ 2Θ ≤ 49.992°), 2435 unique (R_{int} = 0.0606, R_{sigma} = 0.0468) which were used in all calculations. The final R_1 was 0.0377 (I > 2σ(I)) and wR_2 was 0.0792 (all data).

Refinement model description

Number of restraints - 0, number of constraints - unknown.

Details:

```
1. Fixed Uiso
 At 1.2 times of:
  All C(H) groups, All C(H,H) groups
 At 1.5 times of:
  All C(H,H,H) groups
2.a Ternary CH refined with riding coordinates:
 C7(H7)
2.b Secondary CH2 refined with riding coordinates:
 C8(H8A,H8B), C6(H6A,H6B)
2.c Aromatic/amide H refined with riding coordinates:
 C3(H3), C2(H2), C1(H1)
2.d Idealised Me refined as rotating group:
 C11(H11A,H11B,H11C), C10(H10A,H10B,H10C), C9(H9A,H9B,H9C)
```

This report has been created with Olex2, compiled on 2015.01.26 svn.r3150 for OlexSys. Please let us know if there are any errors or if you would like to have additional features.

Datablock barc-akd-294_mo - ellipsoid plot

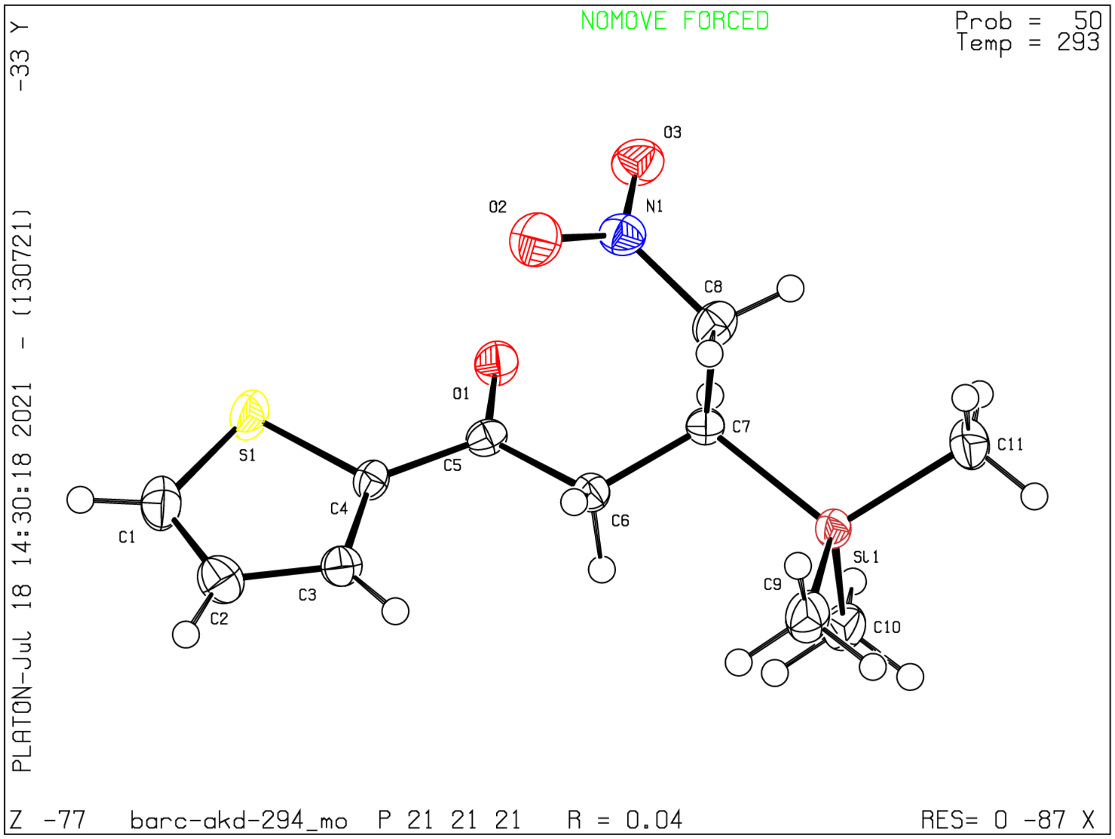

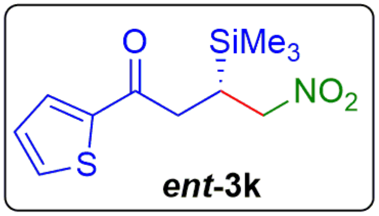

***ent*-3k**
